# Spending on Phased Clinical Development of Approved Drugs by the US National Institutes of Health Compared With Industry

**DOI:** 10.1001/jamahealthforum.2023.1921

**Published:** 2023-07-14

**Authors:** Edward W. Zhou, Matthew J. Jackson, Fred D. Ledley

**Affiliations:** 1 Center for Integration of Science and Industry, Bentley University, Waltham, Massachusetts; 2 Department of Natural and Applied Sciences, Bentley University, Waltham, Massachusetts; 3 Department of Management, Bentley University, Waltham, Massachusetts

## Abstract

**Importance:**

The launch of the Advanced Research Projects Agency for Health to advance new cures and address public concern regarding drug prices has raised questions about the roles of government and industry in drug development.

**Objectives:**

To compare National Institutes of Health (NIH) spending on phased clinical development of approved drugs with that by industry.

**Design:**

This cross-sectional study examined NIH funding for published research reporting the results of phased clinical trials of drugs approved between 2010 and 2019 and compared the findings with reported industry spending estimates. Data analysis was performed between May 2021 and August 2022 using PubMed data from January 1999 through October 2021 and NIH Research Portfolio Online Reporting Tools Expenditures and Results data from January 1999 through December 2020.

**Exposures:**

Drugs approved between 2010 and 2019.

**Main Outcome and Measures:**

National Institutes of Health funding for published research describing applied research on approved drugs, basic research on their biological targets, and phased clinical trials related to drugs approved between 2010 and 2019 were evaluated using Mann-Whitney *U* tests. All costs were inflation adjusted to 2018.

**Results:**

National Institutes of Health funding for basic or applied research related to 386 of 387 drugs approved between 2010 and 2019 totaled $247.3 billion. Of this amount, $8.1 billion (3.3%) was related to phased clinical development. This funding contributed to 12 340 publications on phased clinical trial results involving 240 of 387 (62.0%) drugs. Average NIH spending was $33.8 million per drug, including $13.9 million per drug for phase 1, $22.2 million per drug for phase 2, and $12.9 million per drug for phase 3 trials. Spending by NIH on phased development represented 9.8% to 10.7% of estimated industry spending, including 24.6% to 25.3% of estimated phase 1, 21.4% to 23.2% of phase 2, and 3.7% to 4.3% of phase 3 costs. Considering 60 products for which estimated industry costs were publicly available, NIH spending on clinical trials was significantly lower than estimated industry spending (sum of averages, $54.9 million per drug; mean difference, $326.0 million; 95% CI, $235.6-$416.4 million; 2-tailed paired *t* test *P* < .001). More than 90% of NIH funding came through cooperative agreements or program projects and centers, while 3.3% of NIH funding came through investigator-initiated research projects.

**Conclusions and Relevance:**

In this cross-sectional study, NIH funding for phased clinical development of drugs approved between 2010 and 2019 represented a small fraction of NIH spending on pharmaceutical innovation. This spending focused primarily on early-phase clinical trials and research capacity and was significantly less than estimated industry spending on clinical development. These results may inform the efficient allocation of government funding to advance pharmaceutical innovation.

## Key Points

**Question:**

How much does the US government contribute to phased clinical development of approved drugs compared with industry?

**Findings:**

In this cross-sectional study, phased clinical trials of 387 drugs approved between 2010 and 2019 were associated with $8.1 billion of National Institutes of Health (NIH) funding, primarily for clinical research. This amount represents 3.3% of total NIH funding for basic or applied research related to these products and 9.8% to 10.7% of estimated industry costs, including less than 26% of phase 1 or 2 trials and less than 5% of phase 3 trials.

**Meaning:**

The findings suggest that NIH spending on clinical development focuses on early-stage trials, representing a small fraction of estimated industry spending.

## Teaser

This cross-sectional study uses publicly available publications to compare National Institutes of Health with industry spending on phased clinical development of approved drugs.

## Introduction

The launch of the Advanced Research Projects Agency for Health to develop breakthroughs in order to prevent, detect, and treat diseases such as Alzheimer disease, diabetes, and cancer,^1,2^ coupled with public concern about drug prices, called new attention to the role of government in pharmaceutical innovation. The government has typically focused on funding basic research that leads to the development and commercialization of new products by industry^3,4,5,6,7^ and provides incentives or regulations required to redress market failures.^8,9^ The government has also contributed to applied research,^3,4,10,11,12,13,14^ drug-related patents,^10,12,15,16,17^ and the efficiency of product approvals.^14,18,19^ This analysis examined the US government contribution to development by analyzing National Institutes of Health (NIH) funding related to phased clinical trials of drug products approved between 2010 and 2019.

Previous studies have described NIH funding for basic and applied research related to recent drug approvals.^5,14^ Basic research is defined as “experimental or theoretical work undertaken primarily to acquire new knowledge…without any particular application or use in view,” while applied research is “original investigation undertaken to acquire new knowledge…directed towards a specific practical, aim or objective.”^20^ In contrast, development research is “directed to producing new products or processes or to improving existing products or processes.”^20^ Pharmaceutical development involves a highly structured pathway of preclinical and phased clinical studies designed to satisfy the regulatory requirements for marketing approval of a new product.

Drug development has primarily occurred in industry,^11,21,22^ and 99.4% of the products approved between 2010 and 2019 were sponsored by for-profit firms.^14^ The scale of industry spending on development has been described in various studies.^23,24^ For example, using industry-provided data, DiMasi et al^21^ estimated that average industry spending on development of 106 drugs approved between 1990 and 2010 was $1.5 billion, including the cost of clinical failures, or $2.8 billion after accounting for a 10.5% cost of capital (inflation adjusted to 2018). More recently, using audited financial data reported in accordance with US Generally Accepted Accounting Principles, Wouters et al^25^ estimated that average industry spending on 63 drugs approved between 2009 and 2018 was $374.1 million or $1.5 billion after accounting for clinical failures and a 10.5% cost of capital.

National Institutes of Health spending for published basic and applied science associated with 356 products approved between 2010 and 2019, excluding antimicrobials, has been described by Cleary et al.^5,14,26^ These analyses used a method that involved identifying publications related to the approved drugs or their biological targets in PubMed and the costs associated with NIH-funded projects cited as supporting this research. These studies identified $187 billion in NIH funding before first US Food and Drug Administration (FDA) approval, a value that does not include clinical failures or discount rates,^26^ with 13% representing applied research directly related to the drug and 87% representing basic research directly related to the target.^14^ In these studies, NIH spending on phased clinical trials was included with applied research.

The objective of this study was to characterize NIH funding for phased clinical development of pharmaceutical products. This NIH funding was compared with reported estimates of industry spending on phased clinical trials of approved products.^21,23,24,25^ The results of this analysis describe the scope of the NIH’s contribution to pharmaceutical development and the level of NIH spending on development relative to industry.

## Methods

### Study Design

This cross-sectional study analyzed NIH funding for phased clinical trials related to drugs approved between 2010 and 2019. The study was primarily conducted from May 2021 through August 2022 using publication data from January 1999 through October 2021 and NIH Research Portfolio Online Reporting Tools Expenditures and Results (RePORTER) data from January 1999 through December 2020. This study did not involve human participants per Code of Federal Regulations §46.102(d)(1) and was not subject to institutional review board review. The study was reported in accordance with the Strengthening the Reporting of Observational Studies in Epidemiology (*STROBE*) reporting guideline where applicable.

### Data Sources

Products approved by the FDA between 2010 and 2019 (New Drug Application or Therapeutic Biologics Application), including antimicrobial agents and excluding products derived from blood or tissue as well as diagnostic agents and vaccines, were identified from annual FDA reports.^27,28^ Drug targets were identified from the FDA product label, Therapeutic Target Database, or literature search.^5,14^ Drugs designated as first-in-class, orphan, accelerated, breakthrough, fast track, or priority were identified from FDA reports. Research publications were identified in PubMed. Grant funding data were identified in NIH RePORTER from 2000 through 2020.

### Statistical Analysis

Publications were identified through the National Center for Biotechnology Information PubMed access tool Entrez Molecular Sequence Database System^29^ using Python code.^30^ Publications were designated by PubMed Indexing Number (PMID) and publication year. Search terms were optimized using Boolean search terms after manual review (eTable 1 in Supplement 1). The PMIDs related to drugs were identified by searching for the brand name, active pharmaceutical ingredient, or company chemical identifiers. The PMIDs related to targets were identified by searching for the drug target names and appropriate Medical Subject Heading terms.^5,14^

The PMIDs describing phased clinical trials were identified using PubMed publication type metatags (clinical trial, phase 1, phase 2, phase 3, phase 4, not review) with the Medical Subject Heading term *human*. Publications with a National Clinical Trial designator were identified by automated text analysis of search results (titles, abstracts, and metadata), and the clinical phase described in these studies was determined from ClinicalTrials.gov (eFigure in Supplement 1). The PMIDs describing phase 1/2, phase 2/3, or multiple clinical trials were assigned to the highest clinical phase.

The sensitivity and specificity of identifying phased clinical trials was assessed by manual review of titles and abstracts generated by searching for drug names using a random validation set of 400 PMIDs (eTable 2 in Supplement 1). Assignments were made independently by 2 reviewers (E.W.Z. and M.J.J.), and interreader reliability, sensitivity, and specificity were calculated using standard methods.

The NIH projects and costs associated with PMIDs identified in RePORTER from January 1999 through December 2020, including costs incurred after first FDA approval, were identified using a modification of previous methods^5,14,26^ adapted for Python. Briefly, PMIDs were associated with NIH-funded projects using the RePORTER PMID/Project Link Table and assigned 1 fiscal year of funding corresponding to the year of publication (project year). The PMIDs published before the start date of the project were excluded. To account for publication lags, PMIDs published 1 to 4 years after the end date of the project were assigned to the costs of the last project year. Total PMIDs, project years, and NIH costs were determined after eliminating duplicates. Methods for identifying NIH funding related to approved products using PubMed and RePORTER data sets have been described in the eMethods of Cleary et al.^26^

Mean NIH costs were compared with mean industry costs described by DiMasi et al^21^ or Wouters et al^25^ without including cost of failure or cost of capital. A statistical comparison of NIH and industry costs was also performed for 60 products (eTable 3 in Supplement 1) included in both this study and the work of Wouters et al. All costs were inflation adjusted to 2018. Two-tailed Mann-Whitney *U* tests and 2-tailed paired *t* tests were performed using InStat, version 3.06 software (GraphPad Software LLC). Other analyses were performed using Python, version 3.9.7 (Python Software Foundation) or Excel, Microsoft Office version 2303 (Microsoft Corporation) software. All tests inferred statistical significance at *P* < .05.

## Results

### Published Research on Drugs Approved Between 2010 and 2019

**Figure 1.**
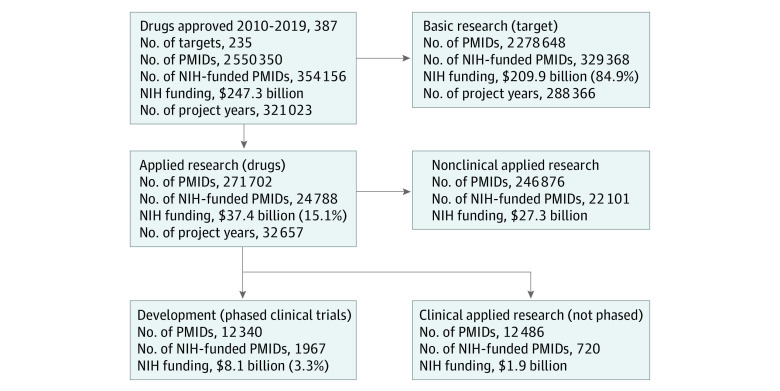
Schematic of National Institutes of Health (NIH) Funding for Basic or Applied Research and Phased Clinical Trials of Drugs Approved, 2010-2019 This analysis identified PubMed Indexing Numbers (PMIDs) related to 387 drugs approved by the US Food and Drug Administration between 2010 and 2019. National Institutes of Health project years and total costs are shown here for basic research on the drug’s biological targets, applied research on the drug as a known molecule, nonphase human clinical trials, and phased clinical trials representing development research. Percentages given are of total NIH investment for all drugs.

The FDA approved 387 drugs between 2010 and 2019 (eTable 1 in Supplement 1), excluding vaccines and biological products derived from natural sources. This data set incorporated 31 drugs not included in the work by Cleary et al,^14,26^ which excluded products with microbial targets. PubMed searches for the 387 drugs identified 271 702 PMIDs categorized as applied research. These drugs were associated with 235 known biological targets. PubMed searches for these targets identified 2 278 648 PMIDs after exclusion of those also identified in drug searches (Figure 1). These PMIDs are categorized as basic research.

### NIH Funding for Basic or Applied Research

Figure 1 shows a schematic of the analysis and results. National Institutes of Health funding was associated with 354 156 of 2 550 350 (13.9%) PMIDs, encompassing 234 of 235 (99.6%) biological targets categorized as basic research, and 355 of 387 (91.7%) drugs categorized as applied research. Combined, NIH funding contributed to basic or applied research for 386 of 387 (99.7%) products approved by the FDA between 2010 and 2019.

National Institutes of Health funding comprised 321 023 project years, including 32 657 (10.1%) supporting applied research on the drug and 288 366 (89.9%) supporting basic research on the biological target. Total NIH funding was $247.3 billion, with $37.4 billion (15.1%) spent on applied research and $209.9 billion (84.9%) billion spent on basic research (Figure 1). These totals are higher than those reported by Cleary et al,^14,26^ which excluded antimicrobial products and considered only NIH costs prior to first FDA approval.^14^

### NIH Funding for Phased Clinical Development

Of all applied (drug) research projects, 24 826 of 271 702 (9.1%) were identified as clinical trial PMIDs, of which 10.8% (2687 of 24 826) received NIH funding. Of these 24 826 clinical trial PMIDs, 12 340 (49.7%) were phased clinical trials, including 1967 (7.9%) PMIDs with NIH funding. Of these 1967 PMIDs, 66 (3.4%) could not be assigned to a clinical phase (Table 1). These included trials related to diet, surgery, diagnostics, or other topics with incidental mention of the drugs in this study.

**Table 1.** Research Publications (PMIDs) Describing Phased Clinical Trials and National Institutes of Health (NIH) Funding, 2010-2019

Development phase	PMIDs, No. (%)^a^	NIH-funded PMIDs, No. (%)^b^	Project years^c^	NIH costs, million $^d^	NIH-funded drugs per phase, No. (%)	Average NIH funding per drug by phase, million $^e^
I	1877 (15.2)	451 (22.9)	550	1460	105 (43.8)	13.9
II	3344 (27.1)	884 (44.9)	1376	3502	158 (65.8)	22.2
III	4278 (34.7)	500 (25.4)	664	2557	198 (82.5)	12.9
IV	450 (3.6)	66 (3.4)	124	427	55 (22.9)	7.8
NCT (others)^f^	2391 (19.4)	66 (3.4)	120	159	22 (9.2)	7.2
Total	12 340 (100)	1967 (100)	2834	8103	240 (100)	33.8

Abbreviations: NCT, National Clinical Trial; PMID, PubMed Indexing Number.

^a^ PMIDs identified by searching for a drug name with publication type phase 1, 2, 3, 4, or NCT number in the abstract and clinical phase identified in ClinicalTrials.gov. Data are based on 240 of 387 (62.0%) drugs approved.

^b^ PMIDs with associated NIH funding in Research Portfolio Online Reporting Tools Expenditures and Results.

^c^ Number of fiscal years of project funding corresponding to the year of publication.

^d^ National Institutes of Health costs for project years. Costs were inflation adjusted to 2018.

^e^ Per-drug spending reflects NIH average investment for funded project years only and does not account for 0 or gaps in spending data.

^f^ PMIDs on clinical trials with an NCT number indicated in the text but no phase designation in ClinicalTrials.gov after manual review.

The sensitivity and specificity of identifying PMIDs describing phased development research among those identified through drug searches was 93.4% and 92.1%, respectively (eTable 2 in Supplement 1). Sensitivity was not estimated for PMIDs identified through target searches due to the large sample size and low frequency.

Funding from the NIH supported phased clinical trials related to 62% (240 of 387) of the drugs, including 105 drugs (43.8%) for phase 1 trials, 158 (65.8%) for phase 2 trials, and 198 (82.5%) for phase 3 trials. National Institutes of Health funding for development comprised 2834 project years of support, with $8.1 billion in NIH costs, representing 21.7% of the NIH costs ($37.4 billion) for applied research or 3.3% ($247.3 billion) of total NIH costs. Of this total, $1.5 billion (18.5%) was associated with phase 1 trials, $3.5 billion (43.2%) with phase 2 trials, $2.6 billion (32.1%) with phase 3 trials, and $0.43 billion (5.3%) with phase 4 trials. These totals correspond to an average NIH spending of $33.8 million per drug, including $13.9 million per drug for phase 1, $22.2 million per drug for phase 2, and $12.9 million per drug for phase 3 trials (Table 1; Figure 1; eTable 4 in Supplement 1).

### Comparing NIH and Industry Spending on Phased Clinical Trials

Average NIH spending for phase 1 to 3 trials was $49.0 million per drug or 10.7% of an estimated $456.7 million per drug industry spending reported by Wouters et al^25^ and 9.8% of an estimated $498.9 million per drug industry spending reported by DiMasi et al^21^ (inflation adjusted to 2018). Average NIH spending for phase 1, phase 2, and phase 3 trials, respectively, was 25.3%, 21.4%, and 4.3% of the estimated industry spending reported by Wouters et al and 24.6%, 23.2%, and 3.7% of the estimated industry spending reported by DiMasi et al (eTable 3 in Supplement 1).

Sixty products described by Wouters et al^25^ were included in this study (Table 2). For these products, average NIH spending was $54.9 million per drug compared with $456.7 million per drug for industry and was significantly lower than industry spending (mean difference, $326.0 million; 95% CI, $235.6-$416.4 million; *P* < .001). National Institutes of Health spending was 19.1% of industry for phase 1 (mean [SD], $10.5 million [21.3 million]; mean difference, $42.6 million; 95% CI, $26.3-$58.9 million; *P* < .001), 28.9% of industry for phase 2 (mean [SD], $30.0 million [$69.2 million]; mean difference, $69.5 million; 95% CI, $32.1-$106.8 million; *P* < .001), and 4.8% of industry for phase 3 (mean [SD], $14.4 million [$44.0 million]; mean difference, $282.6 million; 95% CI, $204.8-$360.4 million; *P* < .001).

**Table 2.** Paired Comparison of National Institutes of Health (NIH) Costs for Development and Industry Costs Estimated by Wouters et al^25^ for 60 Drugs Approved, 2010-2019

Development phase	Mean (SD) NIH costs, million $^a^^,^^b^	Mean (SD) industry cost per drug (Wouters et al), million $^c^^,^^b^	NIH/Wouters et al, %	Mean difference, million $,^b^ (95% CI)	*P* value^d^
I	10.5 (21.3)	54.9 (56.9)	19.1	42.6 (26.3-58.9)	<.001
II	30.0 (69.2)	103.6 (110.1)	28.9	69.5 (32.1-106.8)	<.001
III	14.4 (40.0)	298.3 (272.2)	4.8	282.6 (204.8-360.4)	<.001
Total (phases I-III)	54.9	456.7	12.0	326.0 (235.6-416.4)	<.001

^a^ Paired data of the 60 drugs that were also described in Wouters et al as shown in eTable 5 in their online supplemental material.

^b^ All costs are inflation adjusted to 2018.

^c^ Estimates of industry funding include only estimated phase-specific costs per phase and not costs associated with clinical failures or cost of capital.

^d^ *P* value from 2-tailed paired *t* test.

### NIH Spending on Development of First-in-Class, Orphan, and Expedited Product Approvals

Funding from the NIH for phased clinical development was significantly higher for FDA-designated first-in-class products^31,32^ than for follow-on products by Mann-Whitney *U* test (median, $11.8 million [IQR, $37.5 million] per first-in-class drug vs $1.3 million [IQR, $11.0 million] per follow-on; *P* < .001). This funding was significantly higher for products with orphan (median, $3.2 million [IQR, $34.6 million] vs $1.5 million [IQR, $11.5 million]; *P* = .003), accelerated (median, $8.7 million [IQR, $41.5 million] vs $1.8 million [IQR, $17.0 million]; *P* = .003), fast-track (median, $5.0 million [IQR, $40.3 million] vs $1.5 million [IQR, $12.7 million]; *P* = .003), or priority (median, $4.4 million [IQR, $29.6 million] vs $0.9 million [IQR, $11.1 million]; *P* < .001) designations but not significantly different for products with breakthrough designation (median, $1.3 million [IQR, $11.3 million] vs $2.7 million [IQR, $25.5 million]; *P* = .10) (Table 3).

**Table 3.** National Institutes of Health (NIH) Funding for Products Approved for First-in-Class Products, Orphan Products, and Products Designated for Expedited Review, 2010-2019

Product type	No. of drugs	No. of drugs with NIH-funded applied research (% drugs)^a^	No. of drugs with NIH-funded project support (% drugs)^b^	NIH development costs for initial approval, million $^c^		*P* value^d^
				Mean (SD)	Median (IQR)	
All approvals	385^e^	353 (85)	238 (62)	26.5 (68.4)	27.1 (21.6)	NA
First-in-class	139	137 (99)	101 (73)	30.8 (56.2)	11.8 (37.5)	<.001
Not first-in-class^f^	246	216 (88)	137 (56)	24.1 (74.5)	1.3 (11.0)	
Orphan	166	159 (96)	126 (76)	33.5 (72.3)	3.2 (34.6)	.003
Not orphan	219	194 (89)	112 (51)	21.2 (65.0)	1.5 (11.5)	
Accelerated	49	49 (100)	44 (90)	30.0 (42.0)	8.7 (41.5)	.003
Not accelerated	336	304 (91)	194 (58)	26.0 (71.5)	1.8 (17.0)	
Breakthrough	78	75 (96)	61 (78)	11.1 (24.4)	1.3 (11.3)	.10
Not breakthrough	307	278 (91)	177 (58)	30.4 (75.2)	2.7 (25.5)	
Fast track	142	137 (96)	104 (73)	31.7 (57.3)	5.0 (40.3)	.003
Not fast track	243	216 (89)	134 (55)	23.5 (74.1)	1.5 (12.7)	
Priority	206	203 (99)	152 (74)	29.3 (60.2)	4.4 (29.6)	<.001
Not priority	179	150 (84)	86 (48)	23.3 (76.8)	0.9 (11.1)	
≥1 Designation	226	221 (98)	166 (74)	29.6 (58.8)	4.7 (31.2)	<.001
No expedited	159	132 (83)	72 (45)	22.2 (80.1)	0.5 (9.4)	

Abbreviation: NA, not applicable.

^a^ Drugs with at least 1 PMID identified in search for drug name associated with NIH-funded project in Research Portfolio Online Reporting Tools Expenditures and Results (RePORTER).

^b^ Drugs with at least 1 PubMed Indexing Number (PMID) with an NIH-funded project in RePORTER.

^c^ Of all costs for project-years corresponding to the year of PMID publication with NIH support.

^d^ Difference in NIH costs by 2-tailed Mann-Whitney *U* test.

^e^ A total of 387 drugs approved in 2010-2019. This analysis excludes angiotensin II and parathyroid hormone due to similarity in PMID output for their targets and contamination between applied and basic research project funding totals.

^f^ Also known as follow-on products.

### Categories of NIH-Funded Projects Contributing to Phased Development

Projects funded by the NIH were classified by activity codes that reflect the nature of supported activities. The largest fraction of NIH funding for phased trials came through cooperative agreements, which comprised 960 project years (33.9%) and 59.9% of NIH costs ($4850.0 million), including 47.7% of costs ($696.7 million) for phase 1 trials, 56.0% ($1962.3 million) for phase 2 trials, 68.1% (1741.2 million) for phase 3 trials, and 85.1% ($363.1 million) for phase 4 trials. Manual review of a random set of 300 cooperative agreements of which 264 grants had complete identifiable data in this study showed that 254 (96.2%) projects involved Clinical Translational Science Awards (CTSAs),^33^ clinical trial networks, centers, and consortia involved in clinical research (eTable 5 in Supplement 1).

**Figure 2.**
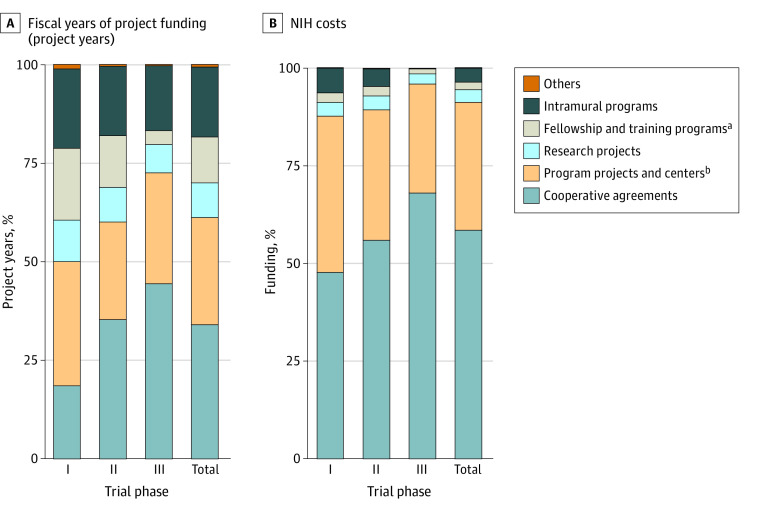
Categories of National Institutes of Health (NIH)-Funded Projects Contributing to Phased Clinical Development of Drugs Approved, 2010-2019 Projects are categorized by activity code. ^a^Includes research career programs, training programs, institutional training, and fellowship programs. ^b^Includes general clinical research centers funded prior to 2005.

Program projects and centers (including general clinical research centers prior to 2005) comprised 751 project years (26.5%) and 31.2% of NIH costs ($2531.3 million) (eTable 5 in Supplement 1). Together, cooperative agreements and program projects and centers comprised more than 60% of project years and more than 90% of NIH costs (Figure 2).

Investigator-initiated research projects only contributed to 281 project years (9.9%) and 3.3% of the NIH cost ($266.4 million) for development research. In contrast, research projects represented the majority of projects (207 897 project years [64.8%]) and 40.3% of NIH costs ($99 730.9 million) for basic and applied research (eTable 5 in Supplement 1). The average per-phase NIH investment on drugs with grant support only was consistent with spending identified in previous case studies^34,35^ as well as estimates from the Southwest Oncology Group^36^ (eTable 6 in Supplement 1).

## Discussion

The objective of this cross-sectional study was to characterize NIH funding for phased clinical trials of drugs approved by the FDA between 2010 and 2019. This analysis identified NIH funding associated with 62.0% of product approvals, totaling $8.1 billion in NIH costs. This total represents 3.3% of all NIH spending for basic or applied research related to these products and 9.8% to 10.7% of the estimated industry spending reported by DiMasi et al^21^ and Wouters et al.^25^ The findings of this study also show that NIH funding for development was proportionally greater for early-stage phase 1 or 2 trials (21.4%-25.3% of estimated industry costs) than for late-stage phase 3 trials (3.7%-4.3% of estimated industry costs) (Figure 1; Table 1; eTable 3 in Supplement 1).

The industry cost estimates by DiMasi et al^21^ and Wouters et al^25^ differ in several important aspects. DiMasi et al^21^ described data on 106 drugs approved between 1990 and 2010 using data provided by 10 large pharmaceutical companies and did not include data from smaller biotechnology firms. Wouters et al^25^ described data on 63 drugs approved between 2014 and 2018 using data extracted from audited financial reports. This method may overrepresent smaller public biotechnology firms for which development costs for individual products may be material to their valuation and itemized in their financial reports.

Our observations are consistent with evidence from previous studies based on case studies or analysis of NIH funding for FDA Orange Book patents that contribute to marketing exclusivity of new products. These studies have shown that as many as 55% of FDA-approved products through 2014 were first synthesized or purified in academic institutions^37^ and that 47.8% of approved products between 1988 and 2005 had patents based on prior art from the public sector.^10^ The fraction of products identified as having NIH funding for development in this study was higher than the 25% of products reported by Nayak et al^13,38^ as having public sector contributions to development. Average NIH spending for phased clinical trials identified in this work was consistent with spending identified in previous case studies,^34,35^ as well as estimates from SWOG^36^ (eTable 6 in Supplement 1).

These results extend the observations of Cleary et al^5,14,26^ that the NIH made substantive investments in basic and applied science related to products approved by the FDA between 2010 and 2019 by delineating the NIH contribution to phased development. Our study used the same methodology as Cleary et al^14,26^ (updated to run in Python) but considered a larger data set, including antimicrobial products, as well as NIH funding for fiscal years after first FDA approval. The larger data set we used included more drugs (387 vs 356) and greater total NIH funding ($247 billion vs $187 billion) than Cleary et al.^26^ While the study by Cleary et al^26^ showed that total NIH investment in basic or applied research related to new products was comparable to estimates of total industry investment in these products, our findings show that NIH spending on phased clinical development is a small fraction of estimated industry costs for this development phase.

This analysis recognized the distinct nature of basic or applied research and the clinical development process. Basic research is generally defined by its focus on the accumulation of new knowledge.^20^ While basic research may be use inspired,^39^ it does not focus on a specific pharmaceutical compound or application. Applied research is distinguished by having specific practical objectives. This research may involve characterization of 1 or more candidate compounds and may lead to drug-related patents that satisfy statutory requirements for product description, utility, and reduction to practice.^40^ In contrast, development is explicitly focused on generating the evidence necessary to achieve approval of a new product. For new drugs, approval requirements includes demonstrating that “the drug is safe and effective in its proposed use(s), and whether the benefits of the drug outweigh the risks; whether the drug’s proposed labeling (package insert) is appropriate, and what it should contain.”^41^ Our analysis confirms the distinct, complementary roles played by the public and private sectors in pharmaceutical innovation by showing that the NIH has made limited contributions to clinical development.

These data also suggest that the nature of the NIH contribution to clinical development has been different than its contribution to basic or applied science. While the largest fraction of NIH funding for basic and applied research was in the form of research projects, which typically fund investigator-initiated research,^14^ this analysis showed that less than 10% of NIH funding for phased clinical trials involved this funding mechanism. Instead, more than 90% of NIH costs for phased clinical development was provided by program projects and centers, which typically support core research capabilities, or collaborative agreements, which typically fund government-initiated research programs and include the CTSA program^33^ administered through the National Center for Advancing Translational Science (NCATS).^42^

The CTSA program and NCATS represent centerpieces in the NIH’s efforts to accelerate innovation by reengineering the clinical research enterprise.^43,44^ Both CTSAs and NCATS focus on advancing the practice of clinical and translational science by providing investigators with new paradigms and processes, a more efficient research infrastructure of translational research hubs, patient networks, clinical consortia, coordinating centers, and institutional review boards, as well as advanced training in clinical, translational, and regulatory science. These funding mechanisms may have contributed indirectly to clinical investigations by providing patient populations, centers for data or laboratory analysis, and training or salaries for clinical investigators but typically do not provide direct funding for investigator-initiated clinical or translational research. The prevalence of these funding mechanisms we observed is consistent with the NIH’s strategic focus on investments designed to streamline clinical and translational science rather than support academic clinical investigation^44,45^ and provides evidence that these mechanisms have contributed to phased clinical trials on the critical path to approval of innovative products. The initial Broad Agency Announcement of funding available from the Advanced Research Projects Agency for Health has extended this focus, describing strategies for achieving health science futures through investments in molecular platforms, biological engineering approaches, foundational advances in degenerative diseases and personalized medicine, artificial intelligence–enabled models, and clinical trial readiness but has not solicited proposals for investigator-initiated clinical trials.^46^

### Limitations

This study has several limitations. First, the analysis is limited by the sensitivity and specificity of the PubMed search methods; the fidelity of metadata used to identify PMIDs describing phased clinical trials; the specificity of associations between PMIDs and NIH-funded projects in RePORTER^47^; and the nonpublication rate for clinical trials, which was reported to be greater than 30% 2 years after FDA approval.^48^ Second, the method for calculating NIH costs included only those project years of project funding corresponding to the year of a PMID reporting study results. This method was consistent with the reported average of 5 publications for 5-year NIH projects^49^ but may underestimate NIH costs associated with clinical studies spanning multiple years. The method accounted for publication lags of 1 to 4 years after the end of project funding, consistent with previously reported lags,^47^ but did not account for lags within the period of project funding, which were not found to affect study results in control experiments.^5,26^ This analysis did not account for data censoring at the end of the study period and may not include NIH spending on unpublished clinical trials, which may lead to an underestimation of NIH costs.

Several factors may limit the comparison of estimated NIH and industry costs. First, the estimates of industry cost used in this analysis estimate actual spending and not the cost of failures or cost of capital typically included in total industry cost.^26^ Second, neither estimates of NIH nor industry costs included chemistry, manufacturing, and control, which could underestimate actual industry spending.^41^ Third, while certain preclinical studies were included in the estimates of industry costs by Wouters et al,^25^ preclinical studies were not included in estimates of industry spending by DiMasi et al^21^ or NIH spending. Fourth, industry costs in Wouters et al^25^ were described according to US Generally Accepted Accounting Principles. These estimates may underestimate industry research and development spending, since the costs of fixed assets, such as facilities, equipment, or technology licenses, are typically capitalized and expensed as depreciation rather than as research and development.

## Conclusions

In this cross-sectional study, NIH funding for phased clinical development of drugs approved between 2010 and 2019 represented a small fraction of the total NIH contribution to pharmaceutical innovation. National Institutes of Health spending focused primarily on early-phase clinical trials and research capacity and was significantly less than the estimated spending on development by the pharmaceutical industry. These results may inform the efficient allocation of government funding in policies designed to accelerate pharmaceutical innovation. Such policies must recognize the role of government as a lead investor in the basic and applied research that enables innovation, as well as the government’s relatively circumscribed contributions to clinical development. Further research is required to understand how the complementary roles of public and private sector investments are associated with the efficiency, costs, and timelines of development as well as with the returns on these investments.

## Notes

Supplement 1. **eFigure.** Analysis Flow Charts From Search Term Inputs to Development Research Funding Data **eTable 1.** Drug and Biological Target Search Terms Used in PMID Extraction **eTable 2.** Estimated Sensitivity and Specificity of Discovered Developmental and Clinical PMIDs **eTable 3.** Overall NIH Per-Phase Average Investment Comparison With Industry **eTable 4.** Total NIH Development Research Investment for Each Drug by Phase **eTable 5.** Total Grant Type Project Years and NIH Funding Distribution for Basic, Applied, and Development Research **eTable 6.** Average Per-Phase NIH Investment on Drugs With Grant Support Only, Similar to SWOG Analysis

Supplement 2.
Data Sharing Statement
