# Supplementary material for: Spending on Phased Clinical Development of Approved Drugs by the US National Institutes of Health Compared With Industry
Source: JAMA Health Forum. 2023 Jul 14;4(7):e231921. doi: 10.1001/jamahealthforum.2023.1921 (PMC10349341; doi:10.1001/jamahealthforum.2023.1921)
Supplement: Supplement 1. — eFigure. Analysis Flow Charts From Search Term Inputs to Development Research Funding Data eTable 1. Drug and Biological Target Search Terms Used in PMID Extraction eTable 2. Estimated Sensitivity and Specificity of Discovered Developmental and Clinical PMIDs eTable 3. Overall NIH Per-Phase Average Investment Comparison With Industry eTable 4. Total NIH Development Research Investment for Each Drug by Phase eTable 5. Total Grant Type Project Years and NIH Funding Distribution for Basic, Applied, and Development Research eTable 6. Average Per-Phase NIH Investment on Drugs With Grant Support Only, Similar to SWOG Analysis [file jamahealthforum-e231921-s001.pdf]

# Supplemental Online Content

Zhou EW, Jackson MJ, Ledley FD. Spending on phased clinical development of approved drugs by the US National Institutes of Health compared with industry. *JAMA Health Forum*. 2023;4(7):e231921. doi:10.1001/jamahealthforum.2023.1921

**eFigure.** Analysis Flow Charts From Search Term Inputs to Development Research Funding Data

**eTable 1.** Drug and Biological Target Search Terms Used in PMID Extraction

**eTable 2.** Estimated Sensitivity and Specificity of Discovered Developmental and Clinical PMIDs

**eTable 3.** Overall NIH Per-Phase Average Investment Comparison With Industry

**eTable 4.** Total NIH Development Research Investment for Each Drug by Phase

**eTable 5.** Total Grant Type Project Years and NIH Funding Distribution for Basic, Applied, and Development Research

**eTable 6.** Average Per-Phase NIH Investment on Drugs With Grant Support Only, Similar to SWOG Analysis

This supplemental material has been provided by the authors to give readers additional information about their work.

© 2023 Zhou EW et al. *JAMA Health Forum.*

**eFigure. Analysis Flow Charts From Search Term Inputs to Development Research Funding Data.** A. Flow chart for identification of PMIDs reporting basic research, applied research, and phased clinical development B. Flow chart for identification of NIH projects, project years, and costs contributing to phased clinical development.

**A.**

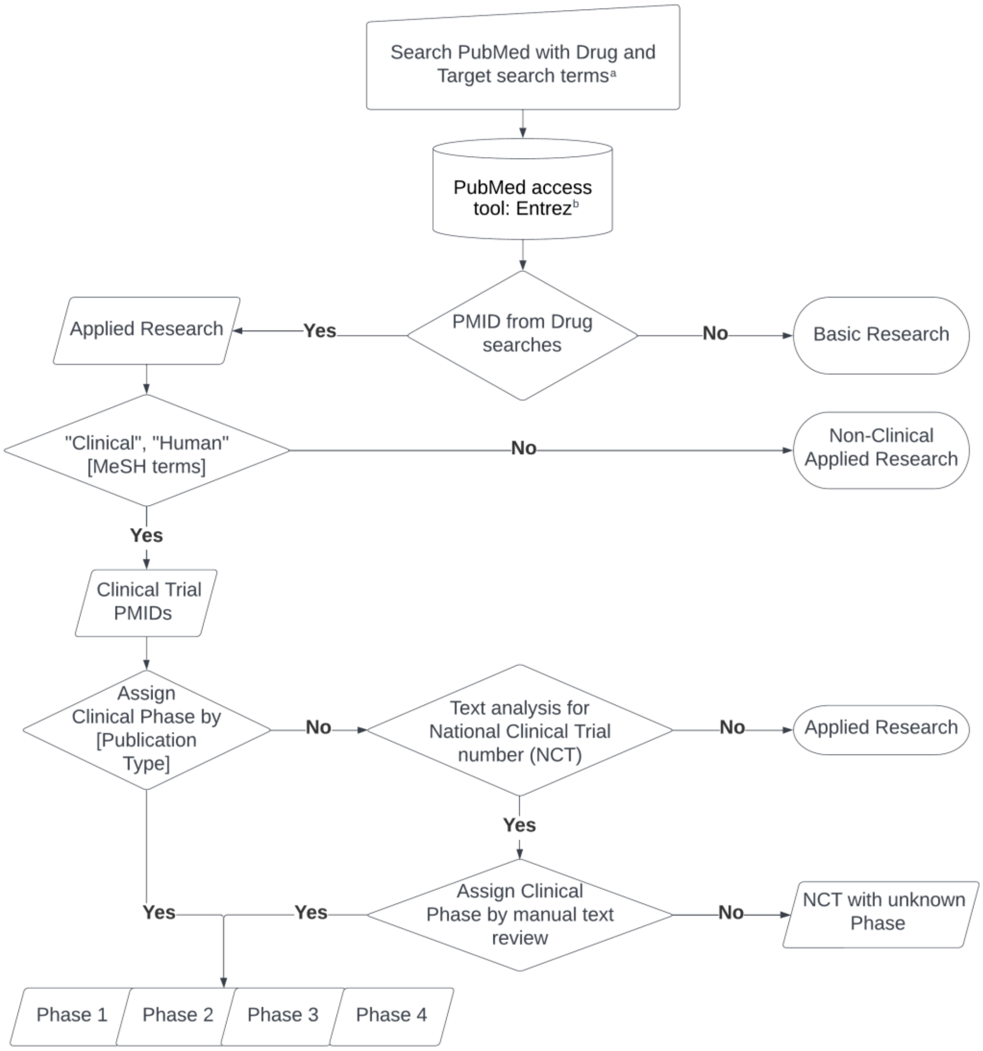


a - Full search terms are in eTable 1. b - National Center for Biotechnology Information (NCBI) PubMed access tool, Entrez Molecular Database System (https://www.ncbi.nlm.nih.gov/Web/Search/entrezfs.html)

© 2023 Zhou EW et al. *JAMA Health Forum.*

**B.**

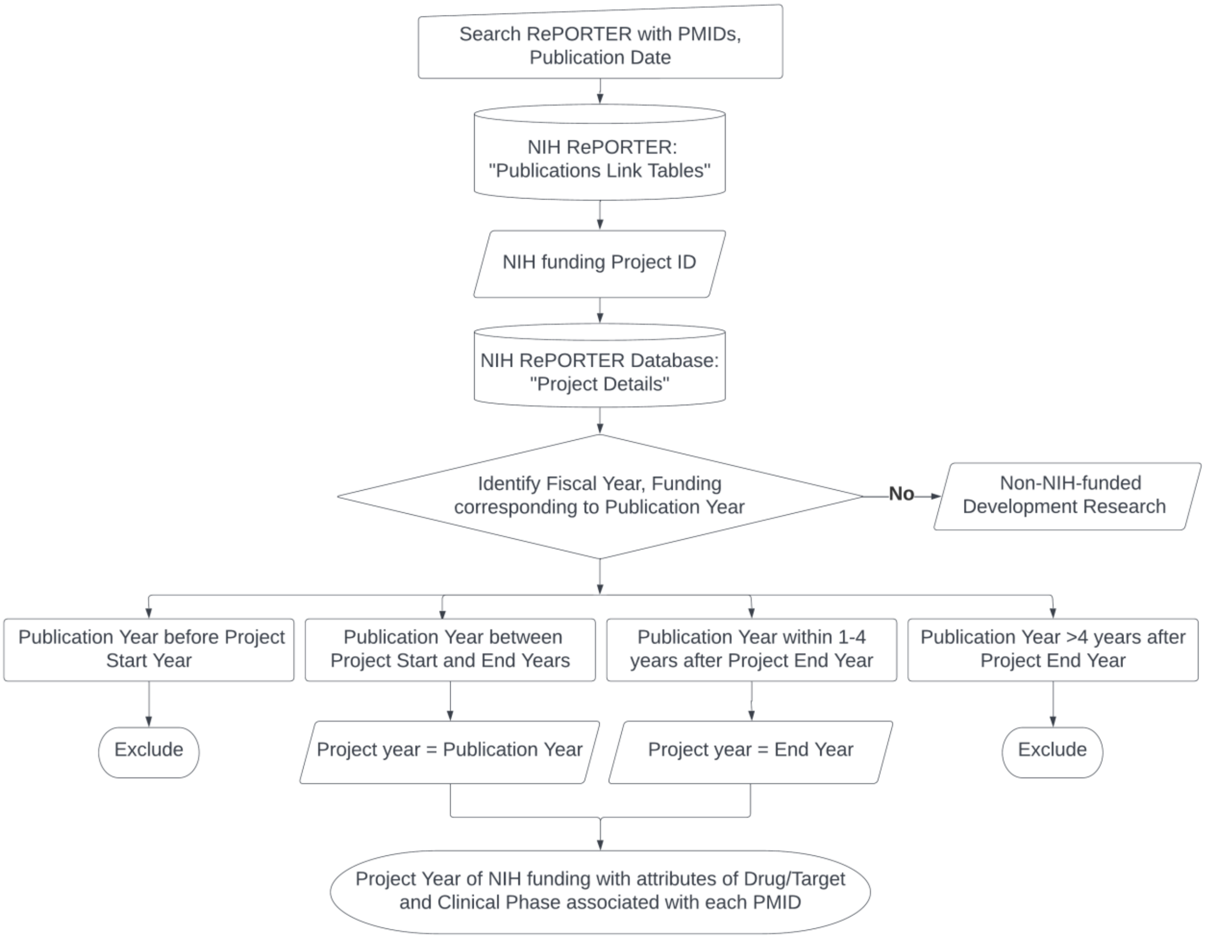


© 2023 Zhou EW et al. *JAMA Health Forum.*

**eTable 1. Drug and Biological Target Search Terms Used in PMID Extraction.** Total number of drugs (n=387).

| Drug ID | Drug PubMed Search Term | Target ID | Target PubMed Search Term | Brand Name | Active Ingredients | FDA Year of Approval |
|---|---|---|---|---|---|---|
| drug 1 | "abiraterone acetate"[MeSH Terms] OR ("abiraterone"[All Fields] AND "acetate"[All Fields]) OR "abiraterone acetate"[All Fields] OR "zytiga"[All Fields] OR "abiraterone"[Supplementary Concept] OR "abiraterone"[All Fields] OR ("abiraterone"[Supplementary Concept] OR "abiraterone"[All Fields]) OR ("abiraterone acetate"[MeSH Terms] OR ("abiraterone"[All Fields] AND "acetate"[All Fields]) OR "abiraterone acetate"[All Fields]) | 49 | (CYP17A1) AND (1980:2020 [pdat]) | Zytiga | Abiraterone | 2011 |
| drug10 | "alirocumab"[Supplementary Concept] OR "alirocumab"[All Fields] OR "praluent"[All Fields] OR "alirocumab"[Supplementary Concept] OR "alirocumab"[All Fields] OR "alirocumab"[Supplementary Concept] OR "alirocumab"[All Fields] | 117 | (proprotein convertase subtilisin kexin type 9) AND (1980:2020 [pdat]) | Praluent | Alirocumab | 2015 |
| drug100 | "ixazomib"[Supplementary Concept] OR "ixazomib"[All Fields] OR "ninlaro"[All Fields] OR ("ixazomib"[Supplementary Concept] OR "ixazomib"[All Fields]) OR (("ixazomib"[Supplementary Concept] OR "ixazomib"[All Fields]) AND ("citrate s"[All Fields] OR "citrates"[MeSH Terms] OR "citrates"[All Fields] OR "citric acid"[MeSH Terms] OR ("citric"[All Fields] AND "acid"[All Fields]) OR "citric acid"[All Fields] OR "citrate"[All Fields])) | 99 | (nf-kappa b) AND (1980:2020 [pdat]) | Ninlaro | Ixazomib | 2015 |

© 2023 Zhou EW et al. *JAMA Health Forum.*

| | | | | | | |
|---|---|---|---|---|---|---|
| drug101 | "ixekizumab"[Supplementary Concept] OR "ixekizumab"[All Fields] OR "taltz"[All Fields] OR "ixekizumab"[Supplementary Concept] OR "ixekizumab"[All Fields] OR "ixekizumab"[Supplementary Concept] OR "ixekizumab"[All Fields] | 82 | (Interleukin 17) AND (1980:2020 [pdat]) | Taltz | Ixekizumab | 2016 |
| drug102 | "ledipasvir"[Supplementary Concept] OR "ledipasvir"[All Fields] OR "ledipasvir sofosbuvir drug combination"[Supplementary Concept] OR "ledipasvir sofosbuvir drug combination"[All Fields] OR "harvoni"[All Fields] OR "sofosbuvir"[MeSH Terms] OR "sofosbuvir"[All Fields] OR (("ledipasvir"[Supplementary Concept] OR "ledipasvir"[All Fields]) AND ("sofosbuvir"[MeSH Terms] OR "sofosbuvir"[All Fields])) OR (("sofosbuvir"[MeSH Terms] OR "sofosbuvir"[All Fields]) AND ("ledipasvir"[Supplementary Concept] OR "ledipasvir"[All Fields])) | 74 | (hcv ns5a) AND (1980:2020 [pdat]) | Harvoni | Ledipasvir plus sofosbuvir | 2014 |
| drug103 | "lenvatinib"[Supplementary Concept] OR "lenvatinib"[All Fields] OR "lenvima"[All Fields] OR "lenvatinib"[Supplementary Concept] OR "lenvatinib"[All Fields] OR "lenvatinib"[Supplementary Concept] OR "lenvatinib"[All Fields] | 123 | (receptor tyrosine kinase) AND (1980:2020 [pdat]) | Lenvima | Lenvatinib | 2015 |
| drug104 | "lesinurad"[Supplementary Concept] OR "lesinurad"[All Fields] OR "zurampic"[All Fields] OR "lesinurad"[Supplementary Concept] OR "lesinurad"[All Fields] OR "lesinurad"[Supplementary Concept] OR "lesinurad"[All Fields] | 147 | (urate transporter OR SLC22A12 OR URAT1) AND (1980:2020 [pdat]) | Zurampic | Lesinurad | 2015 |

© 2023 Zhou EW et al. *JAMA Health Forum.*

| | | | | | | |
|---|---|---|---|---|---|---|
| drug105 | "levomilnacipran"[MeSH Terms] OR "levomilnacipran"[All Fields] OR "fetzima"[All Fields] OR "levomilnacipran"[MeSH Terms] OR "levomilnacipran"[All Fields] OR "levomilnacipran"[MeSH Terms] OR "levomilnacipran"[All Fields] | 7 | ((serotonin reuptake transporter) OR bace1) AND (1980:2020 [pdat]) | Fetzima | levomilnacipran | 2013 |
| drug106 | "lifitegrast"[Supplementary Concept] OR "lifitegrast"[All Fields] OR "xiidra"[All Fields] OR "lifitegrast"[Supplementary Concept] OR "lifitegrast"[All Fields] OR "lifitegrast"[Supplementary Concept] OR "lifitegrast"[All Fields] | 88 | (lymphocyte function-associated antigen-1, LFA-1) AND (1980:2020 [pdat]) | Xiidra | Lifitegrast | 2016 |
| drug107 | "linaclotide"[Supplementary Concept] OR "linaclotide"[All Fields] OR "linzess"[All Fields] OR "linaclotide"[Supplementary Concept] OR "linaclotide"[All Fields] OR "linaclotide"[Supplementary Concept] OR "linaclotide"[All Fields] OR "linaclotide acetate"[All Fields] | 69 | (guanylyl cyclase c) AND (1980:2020 [pdat]) | Linzess | Linaclotide | 2012 |
| drug108 | "linagliptin"[MeSH Terms] OR "linagliptin"[All Fields] OR "tradjenta"[All Fields] OR ("linagliptin"[MeSH Terms] OR "linagliptin"[All Fields]) OR (("linagliptin"[MeSH Terms] OR "linagliptin"[All Fields] OR "bi 1356 bs"[All Fields]) AND "iv"[All Fields]) | 53 | (dipeptidyl-peptidase 4) AND (1980:2020 [pdat]) | Tradjenta | Linagliptin | 2011 |
| drug109 | "liraglutid"[All Fields] OR "liraglutide"[MeSH Terms] OR "liraglutide"[All Fields] OR "victoza"[All Fields] OR "liraglutide s"[All Fields] OR "liraglutid"[All Fields] OR "liraglutide"[MeSH Terms] OR "liraglutide"[All Fields] OR "liraglutide s"[All Fields] OR "liraglutid"[All Fields] OR "liraglutide"[MeSH Terms] OR "liraglutide"[All Fields] OR "liraglutide s"[All Fields] | 67 | (glucagon-like peptide 1) AND (1980:2020 [pdat]) | Victoza | Liraglutide | 2010 |

© 2023 Zhou EW et al. *JAMA Health Forum.*

| | | | | | | |
|---|---|---|---|---|---|---|
| drug11 | "alogliptin"[Supplementary Concept] OR "alogliptin"[All Fields] OR "nesina"[All Fields] OR ("alogliptin"[Supplementary Concept] OR "alogliptin"[All Fields]) OR (("alogliptin"[Supplementary Concept] OR "alogliptin"[All Fields]) AND ("benzoates"[MeSH Terms] OR "benzoates"[All Fields] OR "benzoate"[All Fields])) | 53 | (dipeptidyl-peptidase 4) AND (1980:2020 [pdat]) | Nesina | Alogliptin | 2013 |
| drug110 | "lixisenatide"[Supplementary Concept] OR "lixisenatide"[All Fields] OR "adlyxin"[All Fields] OR "lixisenatide"[Supplementary Concept] OR "lixisenatide"[All Fields] OR "lixisenatide"[Supplementary Concept] OR "lixisenatide"[All Fields] | 67 | (glucagon-like peptide 1) AND (1980:2020 [pdat]) | Adlyxin | Lixisenatide | 2016 |
| drug111 | "bms201038"[Supplementary Concept] OR "bms201038"[All Fields] OR "juxtapid"[All Fields] OR "lomitapide"[All Fields] OR "bms201038"[Supplementary Concept] OR "bms201038"[All Fields] OR "lomitapide"[All Fields] OR "bms201038"[Supplementary Concept] OR "bms201038"[All Fields] OR "lomitapide"[All Fields] | 93 | (Microsomal triglyceride transfer protein) AND (1980:2020 [pdat]) | Juxtapid | Lomitapide | 2012 |
| drug112 | "lorcaserin"[Supplementary Concept] OR "lorcaserin"[All Fields] OR "belviq"[All Fields] OR ("lorcaserin"[Supplementary Concept] OR "lorcaserin"[All Fields]) OR (("lorcaserin"[Supplementary Concept] OR "lorcaserin"[All Fields]) AND ("hydrochlorid"[All Fields] OR "hydrochloride"[All Fields] OR "hydrochlorides"[All Fields])) | 10 | (5-HT2C receptor) AND (1980:2020 [pdat]) | Belviq | Lorcaserin | 2012 |
| drug113 | "lucinactant"[Supplementary Concept] OR "lucinactant"[All Fields] OR "surfaxin"[All Fields] OR "lucinactant"[Supplementary Concept] OR "lucinactant"[All Fields] OR "sinapultide"[Supplementary Concept] OR "sinapultide"[All Fields] | 140 | (surfactant protein B) AND (1980:2020 [pdat]) | Surfaxin | Lucinactant | 2012 |

© 2023 Zhou EW et al. *JAMA Health Forum.*

| | | | | | | |
|---|---|---|---|---|---|---|
| drug114 | "tocilizumab"[Supplementary Concept] OR "tocilizumab"[All Fields] OR "actemra"[All Fields] OR "tocilizumab"[Supplementary Concept] OR "tocilizumab"[All Fields] OR "tocilizumab"[Supplementary Concept] OR "tocilizumab"[All Fields] | 236 | (sterol 14-demethylase) AND (1980:2020 [pdat]) | Luzu | Luliconazole | 2013 |
| drug115 | "ivacaftor"[Supplementary Concept] OR "ivacaftor"[All Fields] OR "lumacaftor"[Supplementary Concept] OR "lumacaftor"[All Fields] OR "lumacaftor ivacaftor drug combination"[Supplementary Concept] OR "lumacaftor ivacaftor drug combination"[All Fields] OR "orkambi"[All Fields] OR (("ivacaftor"[Supplementary Concept] OR "ivacaftor"[All Fields]) AND ("lumacaftor"[Supplementary Concept] OR "lumacaftor"[All Fields])) OR (("lumacaftor"[Supplementary Concept] OR "lumacaftor"[All Fields]) AND ("ivacaftor"[Supplementary Concept] OR "ivacaftor"[All Fields])) | 44 | (CFTR) AND (1980:2020 [pdat]) | Orkambi | Ivacaftor plus lumacaftor | 2015 |
| drug116 | "lurasidone hydrochloride"[MeSH Terms] OR ("lurasidone"[All Fields] AND "hydrochloride"[All Fields]) OR "lurasidone hydrochloride"[All Fields] OR "latuda"[All Fields] OR "lurasidone"[All Fields] OR "lurasidone s"[All Fields] OR ("lurasidone hydrochloride"[MeSH Terms] OR ("lurasidone"[All Fields] AND "hydrochloride"[All Fields]) OR "lurasidone hydrochloride"[All Fields] OR "lurasidone"[All Fields] OR "lurasidone s"[All Fields]) OR ("lurasidone hydrochloride"[MeSH Terms] OR ("lurasidone"[All Fields] AND "hydrochloride"[All Fields]) OR "lurasidone hydrochloride"[All Fields]) | 3 | ((dopamine receptor) OR serotonin receptor) AND (1980:2020 [pdat]) | Latuda | Lurasidone | 2010 |

© 2023 Zhou EW et al. *JAMA Health Forum.*

| | | | | | | |
|---|---|---|---|---|---|---|
| drug117 | "macitentan"[Supplementary Concept] OR "macitentan"[All Fields] OR "opsumit"[All Fields] OR "macitentan"[Supplementary Concept] OR "macitentan"[All Fields] OR "macitentan"[Supplementary Concept] OR "macitentan"[All Fields] | 58 | (endothelin receptor) AND (1980:2020 [pdat]) | Opsumit | Macitentan | 2013 |
| drug118 | "mepolizumab"[Supplementary Concept] OR "mepolizumab"[All Fields] OR "nucala"[All Fields] OR "mepolizumab"[Supplementary Concept] OR "mepolizumab"[All Fields] OR "mepolizumab"[Supplementary Concept] OR "mepolizumab"[All Fields] | 83 | (Interleukin 5) AND (1980:2020 [pdat]) | Nucala | Mepolizumab | 2015 |
| drug119 | "metreleptin"[Supplementary Concept] OR "metreleptin"[All Fields] OR "myalept"[All Fields] OR "metreleptin"[Supplementary Concept] OR "metreleptin"[All Fields] OR "metreleptin"[Supplementary Concept] OR "metreleptin"[All Fields] | 86 | (Leptin) AND (1980:2020 [pdat]) | Myalept | Metreleptin | 2014 |
| drug12 | "apixaban"[Supplementary Concept] OR "apixaban"[All Fields] OR "eliquis"[All Fields] OR "apixaban s"[All Fields] OR "apixaban"[Supplementary Concept] OR "apixaban"[All Fields] OR "apixaban s"[All Fields] OR "apixaban"[Supplementary Concept] OR "apixaban"[All Fields] OR "apixaban s"[All Fields] | 63 | (factor Xa) AND (1980:2020 [pdat]) | Eliquis | Apixaban | 2012 |
| drug120 | "miltefosin"[All Fields] OR "miltefosine"[Supplementary Concept] OR "miltefosine"[All Fields] OR "impavido"[All Fields] OR "miltefosin"[All Fields] OR "miltefosine"[Supplementary Concept] OR "miltefosine"[All Fields] OR "miltefosin"[All Fields] OR "miltefosine"[Supplementary Concept] OR "miltefosine"[All Fields] | 0 | | Impavido | Miltefosine | 2014 |

© 2023 Zhou EW et al. *JAMA Health Forum.*

| | | | | | | |
|---|---|---|---|---|---|---|
| drug121 | "mipomersen"[Supplementary Concept] OR "mipomersen"[All Fields] OR "kynamro"[All Fields] OR "mipomersen"[Supplementary Concept] OR "mipomersen"[All Fields] OR "mipomersen"[Supplementary Concept] OR "mipomersen"[All Fields] OR "mipomersen sodium"[All Fields] | 20 | (APOB OR apolipoprotein B) AND (1980:2020 [pdat]) | Kynamro | Mipomersen | 2013 |
| drug122 | "mirabegron"[Supplementary Concept] OR "mirabegron"[All Fields] OR "myrbetriq"[All Fields] OR "mirabegron"[Supplementary Concept] OR "mirabegron"[All Fields] OR "mirabegron"[Supplementary Concept] OR "mirabegron"[All Fields] | 25 | ("beta 3" AND adrenergic receptor) AND (1980:2020 [pdat]) | Myrbetriq | Mirabegron | 2012 |
| drug123 | "naloxegol"[Supplementary Concept] OR "naloxegol"[All Fields] OR "movantik"[All Fields] OR "naloxegol"[Supplementary Concept] OR "naloxegol"[All Fields] OR "naloxegol"[Supplementary Concept] OR "naloxegol"[All Fields] | 127 | (receptors, opioid, mu[MeSH Terms]) AND (1980:2020 [pdat]) | Movantik | Naloxegol | 2014 |
| drug124 | "necitumumab"[Supplementary Concept] OR "necitumumab"[All Fields] OR "portrazza"[All Fields] OR "necitumumab"[Supplementary Concept] OR "necitumumab"[All Fields] OR "necitumumab"[Supplementary Concept] OR "necitumumab"[All Fields] | 57 | (EGFR) AND (1980:2020 [pdat]) | Portrazza | Necitumumab | 2015 |
| drug125 | "netupitant"[Supplementary Concept] OR "netupitant"[All Fields] OR "netupitant palosentron drug combination"[Supplementary Concept] OR "netupitant palosentron drug combination"[All Fields] OR "akynzeo"[All Fields] OR "palonosetron"[MeSH Terms] OR "palonosetron"[All Fields] OR "palonosetron s"[All Fields] OR (("netupitant"[Supplementary Concept] OR "netupitant"[All Fields]) AND ("palonosetron"[MeSH Terms] OR "palonosetron"[All Fields] OR "palonosetron s"[All Fields])) OR (("netupitant"[Supplementary Concept] OR "netupitant"[All Fields]) AND ("palonosetron"[MeSH Terms] OR | 8 | ((substance p receptor) OR tachykinin receptor 1) AND (1980:2020 [pdat]) | Akynzeo | Netupitant, palonosetron | 2014 |

© 2023 Zhou EW et al. *JAMA Health Forum.*

| | | | | | | |
|---|---|---|---|---|---|---|
| | "palonosetron"[All Fields] OR ("palonosetron"[All Fields] AND "hydrochloride"[All Fields]) OR "palonosetron hydrochloride"[All Fields]) AND "Helsinn"[All Fields]) | | | | | |
| drug126 | "nintedanib"[Supplementary Concept] OR "nintedanib"[All Fields] OR "ofev"[All Fields] OR "nintedanib"[Supplementary Concept] OR "nintedanib"[All Fields] OR "nintedanib"[Supplementary Concept] OR "nintedanib"[All Fields] | 123 | (receptor tyrosine kinase) AND (1980:2020 [pdat]) | Ofev | Nintedanib | 2014 |
| drug127 | "nivolumab"[MeSH Terms] OR "nivolumab"[All Fields] OR "opdivo"[All Fields] OR "nivolumab s"[All Fields] OR "nivolumab"[MeSH Terms] OR "nivolumab"[All Fields] OR "nivolumab s"[All Fields] OR "nivolumab"[MeSH Terms] OR "nivolumab"[All Fields] OR "nivolumab s"[All Fields] | 115 | ("programmed cell death 1") AND (1980:2020 [pdat]) | Opdivo | Nivolumab | 2014 |
| drug128 | "nusinersen"[Supplementary Concept] OR "nusinersen"[All Fields] OR "spinraza"[All Fields] OR "nusinersen"[Supplementary Concept] OR "nusinersen"[All Fields] OR "nusinersen"[Supplementary Concept] OR "nusinersen"[All Fields] | 2 | ((((survival motor neuron protein) OR smn) OR smn1 smn2) OR survival of motor neuron 2 protein[MeSH Terms]) AND (1980:2020 [pdat]) | Spinraza | Nusinersen | 2016 |

© 2023 Zhou EW et al. *JAMA Health Forum.*

| | | | | | | |
|---|---|---|---|---|---|---|
| drug129 | "obeticholic acid"[Supplementary Concept] OR "obeticholic acid"[All Fields] OR "ocaliva"[All Fields] OR "obeticholic acid"[Supplementary Concept] OR "obeticholic acid"[All Fields] OR "obeticholic acid"[Supplementary Concept] OR "obeticholic acid"[All Fields] | 64 | (farnesoid x receptor) AND (1980:2020 [pdat]) | Ocaliva | Obeticholic acid | 2016 |
| drug13 | "apremilast"[Supplementary Concept] OR "apremilast"[All Fields] OR "otezla"[All Fields] OR "apremilast"[Supplementary Concept] OR "apremilast"[All Fields] OR "apremilast"[Supplementary Concept] OR "apremilast"[All Fields] | 107 | (phosphodiesterase 4) AND (1980:2020 [pdat]) | Otezla | Apremilast | 2014 |
| drug130 | "obiltoxaximab"[Supplementary Concept] OR "obiltoxaximab"[All Fields] OR "anthim"[All Fields] OR "obiltoxaximab"[Supplementary Concept] OR "obiltoxaximab"[All Fields] OR "obiltoxaximab"[Supplementary Concept] OR "obiltoxaximab"[All Fields] | 121 | (protective antigen anthrax) AND (1980:2020 [pdat]) | Anthim | Obiltoxaximab | 2016 |
| drug131 | "obinutuzumab"[Supplementary Concept] OR "obinutuzumab"[All Fields] OR "gazyva"[All Fields] OR "obinutuzumab"[Supplementary Concept] OR "obinutuzumab"[All Fields] OR "obinutuzumab"[Supplementary Concept] OR "obinutuzumab"[All Fields] | 37 | (CD20) AND (1980:2020 [pdat]) | Gazyva | Obinutuzumab | 2013 |
| drug132 | "jetrea"[All Fields] OR "microplasmin"[Supplementary Concept] OR "microplasmin"[All Fields] OR "ocriplasmin"[All Fields] OR "microplasmin"[Supplementary Concept] OR "microplasmin"[All Fields] OR "ocriplasmin"[All Fields] OR "microplasmin"[Supplementary Concept] OR "microplasmin"[All Fields] OR "ocriplasmin"[All Fields] | 110 | (plasmin) AND (1980:2020 [pdat]) | Jetrea | Ocriplasmin | 2012 |
| drug133 | "olaparib"[Supplementary Concept] OR "olaparib"[All Fields] OR "lynparza"[All Fields] OR "olaparib"[Supplementary Concept] OR "olaparib"[All Fields] OR "olaparib"[Supplementary Concept] OR "olaparib"[All Fields] | 105 | (PARP) AND (1980:2020 [pdat]) | Lynparza | Olaparib | 2014 |

© 2023 Zhou EW et al. *JAMA Health Forum.*

| | | | | | | |
|---|---|---|---|---|---|---|
| drug134 | "olaratumab"[Supplementary Concept] OR "olaratumab"[All Fields] OR "lartruvo"[All Fields] OR "olaratumab"[Supplementary Concept] OR "olaratumab"[All Fields] OR "olaratumab"[Supplementary Concept] OR "olaratumab"[All Fields] | 129 | (receptors, platelet-derived growth factor[MeSH Terms]) AND (1980:2020 [pdat]) | Lartruvo | Olaratumab | 2016 |
| drug135 | "olodaterol"[Supplementary Concept] OR "olodaterol"[All Fields] OR "striverdi respimat"[All Fields] OR "olodaterol"[Supplementary Concept] OR "olodaterol"[All Fields] OR "olodaterol"[Supplementary Concept] OR "olodaterol"[All Fields] | 28 | (beta-2-adrenergic receptor) AND (1980:2020 [pdat]) | Striverdi Respimat | Olodaterol | 2014 |
| drug136 | "tedizolid phosphate"[Supplementary Concept] OR "tedizolid phosphate"[All Fields] OR "sivextro"[All Fields] OR "tedizolid"[Supplementary Concept] OR "tedizolid"[All Fields] OR "tedizolid"[Supplementary Concept] OR "tedizolid"[All Fields] OR "tedizolid"[Supplementary Concept] OR "tedizolid"[All Fields] | 0 | | Synribo | Omacetaxine mepesuccinate | 2012 |
| drug137 | "viekira pak"[Supplementary Concept] OR "viekira pak"[All Fields] OR "viekira pak"[All Fields] OR ("Ombitasvir"[All Fields] AND ("paritaprevir"[Supplementary Concept] OR "paritaprevir"[All Fields]) AND ("dasabuvir"[Supplementary Concept] OR "dasabuvir"[All Fields]) AND ("ritonavir"[MeSH Terms] OR "ritonavir"[All Fields])) OR ("Ombitasvir"[All Fields] AND ("paritaprevir"[Supplementary Concept] OR "paritaprevir"[All Fields]) AND ("ritonavir"[MeSH Terms] OR "ritonavir"[All Fields]) AND ("dasabuvir"[Supplementary Concept] OR "dasabuvir"[All Fields])) | 73 | (hcv ns5a OR hcv NS3 OR HCV NS5B) AND (1980:2020 [pdat]) | Viekira Pak | Ombitasvir, paritaprevir, dasabuvir, ritonavir | 2014 |

© 2023 Zhou EW et al. *JAMA Health Forum.*

| | | | | | | |
|---|---|---|---|---|---|---|
| drug138 | "secnidazole"[Supplementary Concept] OR "secnidazole"[All Fields] OR "solosec"[All Fields] OR ("secnidazole"[Supplementary Concept] OR "secnidazole"[All Fields]) OR (("secnidazole"[Supplementary Concept] OR "secnidazole"[All Fields]) AND "Symbiomix"[All Fields] AND ("therapeutical"[All Fields] OR "therapeutically"[All Fields] OR "therapeuticals"[All Fields] OR "therapeutics"[MeSH Terms] OR "therapeutics"[All Fields] OR "therapeutic"[All Fields])) | 245 | (Cell wall synthesis) AND (1980:2020 [pdat]) | Orbactive | Oritavancin | 2014 |
| drug139 | "osimertinib"[Supplementary Concept] OR "osimertinib"[All Fields] OR "tagrisso"[All Fields] OR "osimertinib"[Supplementary Concept] OR "osimertinib"[All Fields] OR "osimertinib"[Supplementary Concept] OR "osimertinib"[All Fields] | 57 | (EGFR) AND (1980:2020 [pdat]) | Tagrisso | Osimertinib | 2015 |
| drug14 | "aripiprazole lauroxil"[Supplementary Concept] OR "aripiprazole lauroxil"[All Fields] OR "aristada"[All Fields] OR "aripiprazole lauroxil"[Supplementary Concept] OR "aripiprazole lauroxil"[All Fields] OR "aripiprazole lauroxil"[Supplementary Concept] OR "aripiprazole lauroxil"[All Fields] | 3 | ((dopamine receptor) OR serotonin receptor) AND (1980:2020 [pdat]) | Aristada | Aripiprazole lauroxil | 2015 |
| drug140 | "ospemifene"[Supplementary Concept] OR "ospemifene"[All Fields] OR "ospemifene"[All Fields] OR "osphena"[All Fields] OR "ospemifene"[Supplementary Concept] OR "ospemifene"[All Fields] OR "ospemifene"[All Fields] OR "ospemifene"[Supplementary Concept] OR "ospemifene"[All Fields] OR "ospemifene"[All Fields] | 62 | (estrogen receptor) AND (1980:2020 [pdat]) | Osphena | Ospemifene | 2013 |

© 2023 Zhou EW et al. *JAMA Health Forum.*

| | | | | | | |
|---|---|---|---|---|---|---|
| drug141 | "palbociclib"[Supplementary Concept] OR "palbociclib"[All Fields] OR "ibrance"[All Fields] OR "palbociclib"[Supplementary Concept] OR "palbociclib"[All Fields] OR "palbociclib"[Supplementary Concept] OR "palbociclib"[All Fields] | 41 | (CDK4 or CDK6) AND (1980:2020 [pdat]) | Ibrance | Palbociclib | 2015 |
| drug142 | "panobinostat"[MeSH Terms] OR "panobinostat"[All Fields] OR "farydak"[All Fields] OR "panobinostat"[MeSH Terms] OR "panobinostat"[All Fields] OR "panobinostat"[MeSH Terms] OR "panobinostat"[All Fields] | 78 | (histone deacetylases[MeSH Terms]) AND (1980:2020 [pdat]) | Farydak | Panobinostat | 2015 |
| drug143 | "parathyroid hormone"[MeSH Terms] OR ("parathyroid"[All Fields] AND "hormone"[All Fields]) OR "parathyroid hormone"[All Fields] OR "natpara"[All Fields] OR ("parathyroid hormone"[MeSH Terms] OR ("parathyroid"[All Fields] AND "hormone"[All Fields]) OR "parathyroid hormone"[All Fields]) OR (("parathyroid hormone"[MeSH Terms] OR ("parathyroid"[All Fields] AND "hormone"[All Fields]) OR "parathyroid hormone"[All Fields]) AND "NPS"[All Fields]) | 104 | (parathyroid hormone receptor) AND (1980:2020 [pdat]) | Natpara | Parathyroid hormone | 2015 |
| drug144 | "pasireotide"[Supplementary Concept] OR "pasireotide"[All Fields] OR "signifor"[All Fields] OR "pasireotide"[Supplementary Concept] OR "pasireotide"[All Fields] OR "pasireotide"[Supplementary Concept] OR "pasireotide"[All Fields] | 137 | (somatostatin) AND (1980:2020 [pdat]) | Signifor | Pasireotide | 2012 |
| drug145 | "patiromer"[Supplementary Concept] OR "patiromer"[All Fields] OR "veltassa"[All Fields] OR "patiromer"[Supplementary Concept] OR "patiromer"[All Fields] OR "patiromer"[Supplementary Concept] OR "patiromer"[All Fields] | 0 | | Veltassa | Patiromer | 2015 |

© 2023 Zhou EW et al. *JAMA Health Forum.*

| | | | | | | |
|---|---|---|---|---|---|---|
| drug146 | "Omontys"[All Fields] OR "peginesatide"[Supplementary Concept] OR "peginesatide"[All Fields] OR "peginesatide"[Supplementary Concept] OR "peginesatide"[All Fields] | 60 | (erythropoietin) AND (1980:2020 [pdat]) | Omontys | Peginesatide | 2012 |
| drug147 | "peginterferon beta 1a"[Supplementary Concept] OR "peginterferon beta 1a"[All Fields] OR "peginterferon beta 1a"[All Fields] OR "plegridy"[All Fields] OR ("peginterferon beta 1a"[Supplementary Concept] OR "peginterferon beta 1a"[All Fields] OR "peginterferon beta 1a"[All Fields]) OR ("PEG-interferon"[All Fields] AND "Biogen"[All Fields]) | 81 | (interferon beta 1) AND (1980:2020 [pdat]) | Plegridy | Peginterferon beta-1A | 2014 |
| drug148 | "pegloticase"[Supplementary Concept] OR "pegloticase"[All Fields] OR "krystexxa"[All Fields] OR "pegloticase"[All Fields] OR "pegloticase"[Supplementary Concept] OR "pegloticase"[All Fields] OR "pegloticase"[All Fields] OR "pegloticase"[Supplementary Concept] OR "pegloticase"[All Fields] OR "pegloticase"[All Fields] | 146 | (urate oxidase[MeSH Terms]) AND (1980:2020 [pdat]) | Krystexxa | Pegloticase | 2010 |
| drug149 | "pembrolizumab"[Supplementary Concept] OR "pembrolizumab"[All Fields] OR "keytruda"[All Fields] OR "pembrolizumab"[Supplementary Concept] OR "pembrolizumab"[All Fields] OR "pembrolizumab"[Supplementary Concept] OR "pembrolizumab"[All Fields] | 115 | ("programmed cell death 1") AND (1980:2020 [pdat]) | Keytruda | Pembrolizumab | 2014 |
| drug15 | "asfotase alfa"[Supplementary Concept] OR "asfotase alfa"[All Fields] OR "strensiq"[All Fields] OR "asfotase alfa"[Supplementary Concept] OR "asfotase alfa"[All Fields] OR "asfotase alfa"[Supplementary Concept] OR "asfotase alfa"[All Fields] | 143 | (tissue nonspecific alkaline phosphatase) AND (1980:2020 [pdat]) | Strensiq | Asfotase alfa | 2015 |
| drug150 | "peramivir"[Supplementary Concept] OR "peramivir"[All Fields] OR "rapivab"[All Fields] OR "peramivir"[Supplementary Concept] OR "peramivir"[All Fields] OR "peramivir"[Supplementary Concept] OR "peramivir"[All Fields] | 150 | (viral neuraminidase) AND (1980:2020 [pdat]) | Rapivab | Peramivir | 2014 |

© 2023 Zhou EW et al. *JAMA Health Forum.*

| | | | | | | |
|---|---|---|---|---|---|---|
| drug151 | "fycompa"[All Fields] OR "perampanel"[Supplementary Concept] OR "perampanel"[All Fields] OR "perampanel"[Supplementary Concept] OR "perampanel"[All Fields] OR "perampanel"[Supplementary Concept] OR "perampanel"[All Fields] | 15 | (AMPA 1) AND (1980:2020 [pdat]) | Fycompa | Perampanel | 2012 |
| drug152 | "pertuzumab"[Supplementary Concept] OR "pertuzumab"[All Fields] OR "perjeta"[All Fields] OR "pertuzumab"[Supplementary Concept] OR "pertuzumab"[All Fields] OR "pertuzumab"[Supplementary Concept] OR "pertuzumab"[All Fields] | 77 | (HER2 OR eErb2 OR p185) AND (1980:2020 [pdat]) | Perjeta | Pertuzumab | 2012 |
| drug153 | "citric acid"[MeSH Terms] OR ("citric"[All Fields] AND "acid"[All Fields]) OR "citric acid"[All Fields] OR "magnesium oxide"[MeSH Terms] OR ("magnesium"[All Fields] AND "oxide"[All Fields]) OR "magnesium oxide"[All Fields] OR "picosulfate sodium"[Supplementary Concept] OR "picosulfate sodium"[All Fields] OR "prepopik"[All Fields] OR ("picosulfate"[All Fields] OR "picosulphate"[All Fields]) OR ("picosulfate sodium"[Supplementary Concept] OR "picosulfate sodium"[All Fields] OR "picoprep"[All Fields]) | 0 | | Prepopik | Picosulfate | 2012 |
| drug154 | "pimavanserin"[Supplementary Concept] OR "pimavanserin"[All Fields] OR "nuplazid"[All Fields] OR "pimavanserin"[Supplementary Concept] OR "pimavanserin"[All Fields] OR "pimavanserin"[Supplementary Concept] OR "pimavanserin"[All Fields] OR "pimavanserin tartrate"[All Fields] | 131 | (serotonin receptor 2a) AND (1980:2020 [pdat]) | Nuplazid | Pimavanserin | 2016 |

© 2023 Zhou EW et al. *JAMA Health Forum.*

| | | | | | | |
|---|---|---|---|---|---|---|
| drug155 | "pirfenidone"[Supplementary Concept] OR "pirfenidone"[All Fields] OR "esbriet"[All Fields] OR "pirfenidone"[Supplementary Concept] OR "pirfenidone"[All Fields] OR "pirfenidone"[Supplementary Concept] OR "pirfenidone"[All Fields] | 0 | | Esbriet | Pirfenidone | 2014 |
| drug156 | "asclera"[All Fields] OR "polidocanol"[MeSH Terms] OR "polidocanol"[All Fields] OR "polidocanols"[All Fields] OR "polidocanol"[MeSH Terms] OR "polidocanol"[All Fields] OR "polidocanols"[All Fields] OR "polidocanol"[MeSH Terms] OR "polidocanol"[All Fields] OR "polidocanols"[All Fields] | 0 | | Asclera | Polidocanol | 2010 |
| drug157 | "pomalidomide"[Supplementary Concept] OR "pomalidomide"[All Fields] OR "pomalyst"[All Fields] OR "pomalidomide"[Supplementary Concept] OR "pomalidomide"[All Fields] OR "pomalidomide"[Supplementary Concept] OR "pomalidomide"[All Fields] | 0 | | Pomalyst | Pomalidomide | 2013 |
| drug158 | "ponatinib"[Supplementary Concept] OR "ponatinib"[All Fields] OR "iclusig"[All Fields] OR "ponatinib"[Supplementary Concept] OR "ponatinib"[All Fields] OR "ponatinib"[Supplementary Concept] OR "ponatinib"[All Fields] | 24 | (bcr-abl) AND (1980:2020 [pdat]) | Iclusig | Ponatinib | 2012 |
| drug159 | "ramucirumab"[Supplementary Concept] OR "ramucirumab"[All Fields] OR "cyramza"[All Fields] OR "ramucirumab"[Supplementary Concept] OR "ramucirumab"[All Fields] OR "ramucirumab"[Supplementary Concept] OR "ramucirumab"[All Fields] | 149 | (vascular endothelial growth factor receptor-2[MeSH Terms]) AND (1980:2020 [pdat]) | Cyramza | Ramucirumab | 2014 |
| drug16 | "asparaginase"[MeSH Terms] OR "asparaginase"[All Fields] OR "asparaginases"[All Fields] OR "crisantaspase"[All Fields] OR "erwinaze"[All Fields] OR "erwinia asparaginase"[All Fields] OR (("asparaginase"[MeSH Terms] OR "asparaginase"[All Fields] OR "asparaginases"[All Fields] OR | 21 | (asparaginase[MeSH Terms]) AND (1980:2020 [pdat]) | Erwinaze | Asparaginase Erwinia chrysanthemi | 2011 |

© 2023 Zhou EW et al. *JAMA Health Forum.*

| | | | | | | |
|---|---|---|---|---|---|---|
| | "crisantaspase"[All Fields]) AND ("dickeya chrysanthemi"[MeSH Terms] OR ("dickeya"[All Fields] AND "chrysanthemi"[All Fields]) OR "dickeya chrysanthemi"[All Fields] OR ("erwinia"[All Fields] AND "chrysanthemi"[All Fields]) OR "erwinia chrysanthemi"[All Fields])) OR ("asparaginase"[MeSH Terms] OR "asparaginase"[All Fields] OR "asparaginases"[All Fields] OR "crisantaspase"[All Fields]) | | | | | |
| drug160 | "avibactam"[Supplementary Concept] OR "avibactam"[All Fields] OR "avibactam ceftazidime drug combination"[Supplementary Concept] OR "avibactam ceftazidime drug combination"[All Fields] OR "avycaz"[All Fields] OR "ceftazidime"[MeSH Terms] OR "ceftazidime"[All Fields] OR "ceftazidim"[All Fields] OR (("avibactam"[Supplementary Concept] OR "avibactam"[All Fields]) AND ("ceftazidime"[MeSH Terms] OR "ceftazidime"[All Fields] OR "ceftazidim"[All Fields])) OR (("avibactam"[Supplementary Concept] OR "avibactam"[All Fields]) AND ("ceftazidime"[MeSH Terms] OR "ceftazidime"[All Fields] OR "ceftazidim"[All Fields])) | 248 | (anthrax toxin receptor) AND (1980:2020 [pdat]) | Raxibacumab | Raxibacumab | 2012 |
| drug161 | "regorafenib"[Supplementary Concept] OR "regorafenib"[All Fields] OR "stivarga"[All Fields] OR "regorafenib"[Supplementary Concept] OR "regorafenib"[All Fields] OR "regorafenib"[Supplementary Concept] OR "regorafenib"[All Fields] | 148 | (urea AND kinase inhibitor) AND (1980:2020 [pdat]) | Stivarga | Regorafenib | 2012 |

© 2023 Zhou EW et al. *JAMA Health Forum.*

| | | | | | | |
|---|---|---|---|---|---|---|
| drug162 | "reslizumab"[Supplementary Concept] OR "reslizumab"[All Fields] OR "cinqair"[All Fields] OR "reslizumab"[Supplementary Concept] OR "reslizumab"[All Fields] OR "reslizumab"[Supplementary Concept] OR "reslizumab"[All Fields] | 83 | (Interleukin 5) AND (1980:2020 [pdat]) | Cinqair | Reslizumab | 2016 |
| drug163 | "edurant"[All Fields] OR "rilpivirine"[MeSH Terms] OR "rilpivirine"[All Fields] OR "rilpivirine"[MeSH Terms] OR "rilpivirine"[All Fields] OR "rilpivirine"[MeSH Terms] OR "rilpivirine"[All Fields] | 66 | (HIV reverse transcriptase) AND (1980:2020 [pdat]) | Edurant | Rilpivirine | 2011 |
| drug164 | "riociguat"[Supplementary Concept] OR "riociguat"[All Fields] OR "adempas"[All Fields] OR "riociguat"[Supplementary Concept] OR "riociguat"[All Fields] OR "riociguat"[Supplementary Concept] OR "riociguat"[All Fields] | 136 | ("soluble guanylate cyclase") AND (1980:2020 [pdat]) | Adempas | Riociguat | 2013 |
| drug165 | "rivaroxaban"[MeSH Terms] OR "rivaroxaban"[All Fields] OR "xarelto"[All Fields] OR "rivaroxaban"[MeSH Terms] OR "rivaroxaban"[All Fields] OR "rivaroxaban"[MeSH Terms] OR "rivaroxaban"[All Fields] | 48 | (coagulation factor X) AND (1980:2020 [pdat]) | Xarelto | Rivaroxaban | 2011 |
| drug166 | "roflumilast"[Supplementary Concept] OR "roflumilast"[All Fields] OR "daliresp"[All Fields] OR "roflumilast"[All Fields] OR "roflumilast"[Supplementary Concept] OR "roflumilast"[All Fields] OR "roflumilast"[All Fields] OR "roflumilast"[Supplementary Concept] OR "roflumilast"[All Fields] OR "roflumilast"[All Fields] | 107 | (phosphodiesterase 4) AND (1980:2020 [pdat]) | Daliresp | Roflumilast | 2011 |
| drug167 | "rolapitant"[Supplementary Concept] OR "rolapitant"[All Fields] OR "varubi"[All Fields] OR "rolapitant"[Supplementary Concept] OR "rolapitant"[All Fields] OR "rolapitant"[Supplementary Concept] OR "rolapitant"[All Fields] | 8 | ((substance p receptor) OR tachykinin receptor 1) AND (1980:2020 [pdat]) | Varubi | Rolapitant | 2015 |

© 2023 Zhou EW et al. *JAMA Health Forum.*

| | | | | | | |
|---|---|---|---|---|---|---|
| drug168 | "rucaparib"[Supplementary Concept] OR "rucaparib"[All Fields] OR "rubraca"[All Fields] OR "rucaparib"[Supplementary Concept] OR "rucaparib"[All Fields] OR "rucaparib"[Supplementary Concept] OR "rucaparib"[All Fields] | 111 | (poly adp ribose polymerase) AND (1980:2020 [pdat]) | Rubraca | Rucaparib | 2016 |
| drug169 | "incb018424"[Supplementary Concept] OR "incb018424"[All Fields] OR "ruxolitinib"[All Fields] OR "jakafi"[All Fields] OR "incb018424"[Supplementary Concept] OR "incb018424"[All Fields] OR "ruxolitinib"[All Fields] OR "incb018424"[Supplementary Concept] OR "incb018424"[All Fields] OR "ruxolitinib"[All Fields] | 85 | (janus kinases[MeSH Terms]) AND (1980:2020 [pdat]) | Jakafi | Ruxolitinib | 2011 |
| drug17 | "atezolizumab"[Supplementary Concept] OR "atezolizumab"[All Fields] OR "tecentriq"[All Fields] OR "atezolizumab"[Supplementary Concept] OR "atezolizumab"[All Fields] OR "atezolizumab"[Supplementary Concept] OR "atezolizumab"[All Fields] | 116 | (programmed cell death-ligand 1 OR PDL1) AND (1980:2020 [pdat]) | Tecentriq | Atezolizumab | 2016 |
| drug170 | "sacubitril and valsartan sodium hydrate drug combination"[Supplementary Concept] OR "sacubitril and valsartan sodium hydrate drug combination"[All Fields] OR "entresto"[All Fields] OR "sacubitril"[Supplementary Concept] OR "sacubitril"[All Fields] OR "valsartan"[MeSH Terms] OR "valsartan"[All Fields] OR (("sacubitril"[Supplementary Concept] OR "sacubitril"[All Fields]) AND ("valsartan"[MeSH Terms] OR "valsartan"[All Fields])) OR (("sacubitril"[Supplementary Concept] OR "sacubitril"[All Fields]) AND ("valsartan"[MeSH Terms] OR "valsartan"[All Fields])) | 98 | (Neutral Endopeptidase) AND (1980:2020 [pdat]) | Entresto | Sacubitril, valsartan | 2015 |

© 2023 Zhou EW et al. *JAMA Health Forum.*

| | | | | | | |
|---|---|---|---|---|---|---|
| drug171 | "sebelipase alfa"[Supplementary Concept] OR "sebelipase alfa"[All Fields] OR "kanuma"[All Fields] OR "sebelipase alfa"[All Fields] OR "sebelipase alfa"[Supplementary Concept] OR "sebelipase alfa"[All Fields] OR "sebelipase alfa"[All Fields] OR "sebelipase alfa"[Supplementary Concept] OR "sebelipase alfa"[All Fields] OR "sebelipase alfa"[All Fields] | 89 | (lysosomal acid lipase) AND (1980:2020 [pdat]) | Kanuma | Sebelipase alfa | 2015 |
| drug172 | "secukinumab"[Supplementary Concept] OR "secukinumab"[All Fields] OR "cosentyx"[All Fields] OR "secukinumab"[Supplementary Concept] OR "secukinumab"[All Fields] OR "secukinumab"[Supplementary Concept] OR "secukinumab"[All Fields] | 82 | (Interleukin 17) AND (1980:2020 [pdat]) | Cosentyx | Secukinumab | 2015 |
| drug173 | "selexipag"[Supplementary Concept] OR "selexipag"[All Fields] OR "uptravi"[All Fields] OR "selexipag"[Supplementary Concept] OR "selexipag"[All Fields] OR "selexipag"[Supplementary Concept] OR "selexipag"[All Fields] | 125 | (receptors, epoprostenol[MeSH Terms]) AND (1980:2020 [pdat]) | Uptravi | Selexipag | 2015 |
| drug174 | "siltuximab"[Supplementary Concept] OR "siltuximab"[All Fields] OR "sylvant"[All Fields] OR "siltuximab"[Supplementary Concept] OR "siltuximab"[All Fields] OR "siltuximab"[Supplementary Concept] OR "siltuximab"[All Fields] | 84 | (interleukin 6 receptor) AND (1980:2020 [pdat]) | Sylvant | Siltuximab | 2014 |
| drug175 | "simeprevir"[MeSH Terms] OR "simeprevir"[All Fields] OR "olysio"[All Fields] OR "simeprevir"[MeSH Terms] OR "simeprevir"[All Fields] OR "simeprevir"[MeSH Terms] OR "simeprevir"[All Fields] | 72 | (HCV NS3) AND (1980:2020 [pdat]) | Olysio | Simeprevir | 2013 |
| drug176 | "sofosbuvir"[MeSH Terms] OR "sofosbuvir"[All Fields] OR "sovaldi"[All Fields] OR "sofosbuvir"[MeSH Terms] OR "sofosbuvir"[All Fields] OR "sofosbuvir"[MeSH Terms] OR "sofosbuvir"[All Fields] | 75 | (HCV NS5B) AND (1980:2020 [pdat]) | Sovaldi | Sofosbuvir | 2013 |

© 2023 Zhou EW et al. *JAMA Health Forum.*

| | | | | | | |
|---|---|---|---|---|---|---|
| drug177 | "sonidegib"[Supplementary Concept] OR "sonidegib"[All Fields] OR "odomzo"[All Fields] OR "sonidegib"[Supplementary Concept] OR "sonidegib"[All Fields] OR "sonidegib"[Supplementary Concept] OR "sonidegib"[All Fields] | 134 | (smoothened) AND (1980:2020 [pdat]) | Odomzo | Sonidegib | 2015 |
| drug178 | "natroba"[All Fields] OR "spinosad"[Supplementary Concept] OR "spinosad"[All Fields] OR "spinosad"[Supplementary Concept] OR "spinosad"[All Fields] OR "spinosad"[Supplementary Concept] OR "spinosad"[All Fields] | 124 | (receptors, cholinergic[MeSH Terms]) AND (1980:2020 [pdat]) | Natroba | Spinosad | 2011 |
| drug179 | "sugammadex"[MeSH Terms] OR "sugammadex"[All Fields] OR "bridion"[All Fields] OR ("sugammadex"[MeSH Terms] OR "sugammadex"[All Fields]) OR ("sugammadex"[MeSH Terms] OR "sugammadex"[All Fields] OR ("sugammadex"[All Fields] AND "sodium"[All Fields]) OR "sugammadex sodium"[All Fields]) | 130 | (Rocuronium OR Vecuronium) AND (1980:2020 [pdat]) | Bridion | Sugammadex | 2015 |
| drug18 | "avanafil"[Supplementary Concept] OR "avanafil"[All Fields] OR "stendra"[All Fields] OR "avanafil"[Supplementary Concept] OR "avanafil"[All Fields] OR "avanafil"[Supplementary Concept] OR "avanafil"[All Fields] | 108 | (phosphodiesterase 5) AND (1980:2020 [pdat]) | Stendra | Avanafil | 2012 |
| drug180 | "suvorexant"[Supplementary Concept] OR "suvorexant"[All Fields] OR "belsomra"[All Fields] OR "suvorexant"[Supplementary Concept] OR "suvorexant"[All Fields] OR "suvorexant"[Supplementary Concept] OR "suvorexant"[All Fields] | 101 | (orexin receptor OR hypocretin receptor) AND (1980:2020 [pdat]) | Belsomra | Suvorexant | 2014 |
| drug181 | "tafluprost"[Supplementary Concept] OR "tafluprost"[All Fields] OR "zioptan"[All Fields] OR "tafluprost"[Supplementary Concept] OR "tafluprost"[All Fields] OR "tafluprost"[Supplementary Concept] OR "tafluprost"[All Fields] | 118 | (Prostaglandin F receptor) AND (1980:2020 [pdat]) | Zioptan | Tafluprost | 2012 |

© 2023 Zhou EW et al. *JAMA Health Forum.*

| | | | | | | |
|---|---|---|---|---|---|---|
| drug182 | "taliglucerase alfa"[Supplementary Concept] OR "taliglucerase alfa"[All Fields] OR "elelyso"[All Fields] OR "taliglucerase alfa"[Supplementary Concept] OR "taliglucerase alfa"[All Fields] OR "taliglucerase alfa"[Supplementary Concept] OR "taliglucerase alfa"[All Fields] | 26 | (beta glucocerebrosidase) AND (1980:2020 [pdat]) | Elelyso | Taliglucerase alfa | 2012 |
| drug183 | "hetlioz"[All Fields] OR "tasimelteon"[Supplementary Concept] OR "tasimelteon"[All Fields] OR "tasimelteon"[Supplementary Concept] OR "tasimelteon"[All Fields] OR "tasimelteon"[Supplementary Concept] OR "tasimelteon"[All Fields] | 91 | (melatonin receptor) AND (1980:2020 [pdat]) | Hetlioz | Tasimelteon | 2014 |
| drug184 | "meropenem and vaborbactam"[Supplementary Concept] OR "meropenem and vaborbactam"[All Fields] OR "vabomere"[All Fields] OR "meropenem"[MeSH Terms] OR "meropenem"[All Fields] OR "vaborbactam"[Supplementary Concept] OR "vaborbactam"[All Fields] OR ("meropenem and vaborbactam"[Supplementary Concept] OR "meropenem and vaborbactam"[All Fields] OR "meropenem vaborbactam"[All Fields]) OR (("meropenem and vaborbactam"[Supplementary Concept] OR "meropenem and vaborbactam"[All Fields]) AND "Melinta"[All Fields] AND ("therapeutical"[All Fields] OR "therapeutically"[All Fields] OR "therapeuticals"[All Fields] OR "therapeutics"[MeSH Terms] OR "therapeutics"[All Fields] OR "therapeutic"[All Fields])) | 241 | (Leucyl-tRNA synthetase) AND (1980:2020 [pdat]) | Kerydin | Tavaborole | 2014 |

© 2023 Zhou EW et al. *JAMA Health Forum.*

| | | | | | | |
|---|---|---|---|---|---|---|
| drug185 | "ozenoxacin"[Supplementary Concept] OR "ozenoxacin"[All Fields] OR "xepi"[All Fields] OR "ozenoxacin"[Supplementary Concept] OR "ozenoxacin"[All Fields] OR "ozenoxacin"[Supplementary Concept] OR "ozenoxacin"[All Fields] | 240 | (P site at the ribosomal 50S subunit) AND (1980:2020 [pdat]) | Sivextro | Tedizolid | 2014 |
| drug186 | "gattex"[All Fields] OR "teduglutide"[Supplementary Concept] OR "teduglutide"[All Fields] OR "teduglutide"[Supplementary Concept] OR "teduglutide"[All Fields] OR "teduglutide"[Supplementary Concept] OR "teduglutide"[All Fields] | 5 | ((glucagon-like peptide 2 or glp2 or glp-2)) AND (1980:2020 [pdat]) | Gattex | Teduglutide | 2012 |
| drug187 | "telaprevir"[Supplementary Concept] OR "telaprevir"[All Fields] OR "incivek"[All Fields] OR "telaprevir"[Supplementary Concept] OR "telaprevir"[All Fields] OR "telaprevir"[Supplementary Concept] OR "telaprevir"[All Fields] | 72 | (HCV NS3) AND (1980:2020 [pdat]) | Incivek | Telaprevir | 2011 |
| drug188 | "teriflunomide"[Supplementary Concept] OR "teriflunomide"[All Fields] OR "aubagio"[All Fields] OR "teriflunomide"[Supplementary Concept] OR "teriflunomide"[All Fields] OR "teriflunomide"[Supplementary Concept] OR "teriflunomide"[All Fields] | 52 | (dihydroorotate dehydrogenase) AND (1980:2020 [pdat]) | Aubagio | Teriflunomide | 2012 |
| drug189 | "tesamorelin"[Supplementary Concept] OR "tesamorelin"[All Fields] OR "egrifta"[All Fields] OR ("tesamorelin"[Supplementary Concept] OR "tesamorelin"[All Fields]) OR (("tesamorelin"[Supplementary Concept] OR "tesamorelin"[All Fields]) AND ("acetalization"[All Fields] OR | 68 | (growth hormone-releasing hormone[MeSH Terms]) AND (1980:2020 [pdat]) | Egrifta | Tesamorelin | 2010 |

© 2023 Zhou EW et al. *JAMA Health Forum.*

| | | | | | | |
|---|---|---|---|---|---|---|
| | "acetalizations"[All Fields] OR "acetalized"[All Fields] OR "acetals"[MeSH Terms] OR "acetals"[All Fields] OR "acetal"[All Fields] OR "acetates"[MeSH Terms] OR "acetates"[All Fields] OR "acetate"[All Fields] OR "acetic"[All Fields])) | | | | | |
| drug19 | "baricitinib"[Supplementary Concept] OR "baricitinib"[All Fields] OR "olumiant"[All Fields] OR "baricitinib"[Supplementary Concept] OR "baricitinib"[All Fields] OR "baricitinib"[Supplementary Concept] OR "baricitinib"[All Fields] | 246 | (beta lactamases) AND (1980:2020 [pdat]) | Avycaz | Avibactam plus ceftazidime | 2015 |
| drug190 | "ticagrelor"[MeSH Terms] OR "ticagrelor"[All Fields] OR "brilinta"[All Fields] OR "ticagrelor"[MeSH Terms] OR "ticagrelor"[All Fields] OR "ticagrelor"[MeSH Terms] OR "ticagrelor"[All Fields] | 103 | (p2y receptor) AND (1980:2020 [pdat]) | Brilinta | Ticagrelor | 2011 |
| drug191 | "tipiracil"[Supplementary Concept] OR "tipiracil"[All Fields] OR "trifluridine tipiracil drug combination"[Supplementary Concept] OR "trifluridine tipiracil drug combination"[All Fields] OR "lonsurf"[All Fields] OR "trifluridine"[MeSH Terms] OR "trifluridine"[All Fields] OR (("tipiracil"[Supplementary Concept] OR "tipiracil"[All Fields]) AND ("trifluridine"[MeSH Terms] OR "trifluridine"[All Fields])) OR (("trifluridine tipiracil drug combination"[Supplementary Concept] OR "trifluridine tipiracil drug combination"[All Fields] OR "trifluridine and tipiracil"[All Fields]) AND ("hydrochlorid"[All Fields] OR "hydrochloride"[All Fields] OR "hydrochlorides"[All Fields]) AND "Servier"[All Fields]) | 142 | (thymidine phosphorylase) AND (1980:2020 [pdat]) | Lonsurf | Tipiracil plus trifluridine | 2015 |

© 2023 Zhou EW et al. *JAMA Health Forum.*

| | | | | | | |
|---|---|---|---|---|---|---|
| drug192 | "luliconazole"[Supplementary Concept] OR "luliconazole"[All Fields] OR "luzu"[All Fields] OR "luliconazole"[Supplementary Concept] OR "luliconazole"[All Fields] OR "luliconazole"[Supplementary Concept] OR "luliconazole"[All Fields] | 84 | (interleukin 6 receptor) AND (1980:2020 [pdat]) | Actemra | Tocilizumab | 2010 |
| drug193 | "tofacitinib"[Supplementary Concept] OR "tofacitinib"[All Fields] OR "xeljanz"[All Fields] OR "tofacitinib s"[All Fields] OR "tofacitinib"[Supplementary Concept] OR "tofacitinib"[All Fields] OR "tofacitinib s"[All Fields] OR "tofacitinib"[Supplementary Concept] OR "tofacitinib"[All Fields] OR "tofacitinib s"[All Fields] | 85 | (janus kinases[MeSH Terms]) AND (1980:2020 [pdat]) | Xeljanz | Tofacitinib | 2012 |
| drug194 | "trabectedin"[MeSH Terms] OR "trabectedin"[All Fields] OR "yondelis"[All Fields] OR "trabectedin"[MeSH Terms] OR "trabectedin"[All Fields] OR "trabectedin"[MeSH Terms] OR "trabectedin"[All Fields] | 0 | | Yondelis | Trabectedin | 2015 |
| drug195 | "mekinist"[All Fields] OR "trametinib"[Supplementary Concept] OR "trametinib"[All Fields] OR "trametinib"[Supplementary Concept] OR "trametinib"[All Fields] OR "trametinib"[Supplementary Concept] OR "trametinib"[All Fields] | 96 | (mitogen-activated protein kinase kinases[MeSH Terms]) AND (1980:2020 [pdat]) | Mekinist | Trametinib | 2013 |
| drug196 | "ella"[All Fields] OR "ulipristal"[Supplementary Concept] OR "ulipristal"[All Fields] OR "ulipristal"[Supplementary Concept] OR "ulipristal"[All Fields] OR "ulipristal acetate"[Supplementary Concept] OR "ulipristal acetate"[All Fields] | 114 | (progesterone receptor[MeSH Terms]) AND (1980:2020 [pdat]) | Ella | Ulipristal | 2010 |

© 2023 Zhou EW et al. *JAMA Health Forum.*

| | | | | | | |
|---|---|---|---|---|---|---|
| drug197 | (("gsk573719"[Supplementary Concept] OR "gsk573719"[All Fields] OR "umeclidinium"[All Fields] OR "anoro"[All Fields] OR "vilanterol"[Supplementary Concept] OR "vilanterol"[All Fields]) AND "Ellipta"[All Fields]) OR (("gsk573719"[Supplementary Concept] OR "gsk573719"[All Fields] OR "umeclidinium"[All Fields]) AND ("vilanterol"[Supplementary Concept] OR "vilanterol"[All Fields])) OR (("gsk573719"[Supplementary Concept] OR "gsk573719"[All Fields] OR "umeclidinium"[All Fields]) AND ("bromid"[All Fields] OR "bromide s"[All Fields] OR "bromides"[MeSH Terms] OR "bromides"[All Fields] OR "bromide"[All Fields]) AND ("vilanterol"[Supplementary Concept] OR "vilanterol"[All Fields])) | 28 | (beta-2-adrenergic receptor) AND (1980:2020 [pdat]) | Anoro Ellipta | Umeclidinium, vilanterol | 2013 |
| drug198 | "uridine triacetate"[Supplementary Concept] OR "uridine triacetate"[All Fields] OR "xuriden"[All Fields] OR "uridine triacetate"[Supplementary Concept] OR "uridine triacetate"[All Fields] OR "uridine triacetate"[Supplementary Concept] OR "uridine triacetate"[All Fields] | 0 | (("fluorouracil"[MeSH Terms] OR ("capecitabine"[MeSH Terms]) AND (1980:2020 [pdat]) | Xuriden | Uridine triacetate | 2015 |
| drug199 | "n 4 bromo 2 fluorophenyl 6 methoxy 7 1 methylpiperidin 4 yl methoxy quinazolin 4 amine"[Supplementary Concept] OR "n 4 bromo 2 fluorophenyl 6 methoxy 7 1 methylpiperidin 4 yl methoxy quinazolin 4 amine"[All Fields] OR "caprelsa"[All Fields] OR "vandetanib"[All Fields] OR "n 4 bromo 2 fluorophenyl 6 methoxy 7 1 methylpiperidin 4 yl methoxy quinazolin 4 amine"[Supplementary Concept] OR "n 4 bromo 2 fluorophenyl 6 | 149 | (vascular endothelial growth factor receptor-2[MeSH Terms]) AND (1980:2020 [pdat]) | Caprelsa | Vandetanib | 2011 |

© 2023 Zhou EW et al. *JAMA Health Forum.*

| | | | | | | |
|---|---|---|---|---|---|---|
| | methoxy 7 1 methylpiperidin 4 yl methoxy quinazolin 4 amine"[All Fields] OR "vandetanib"[All Fields] OR "n 4 bromo 2 fluorophenyl 6 methoxy 7 1 methylpiperidin 4 yl methoxy quinazolin 4 amine"[Supplementary Concept] OR "n 4 bromo 2 fluorophenyl 6 methoxy 7 1 methylpiperidin 4 yl methoxy quinazolin 4 amine"[All Fields] OR "vandetanib"[All Fields] | | | | | |
| drug2 | (("aclidinium"[All Fields] OR "tudorza"[All Fields]) AND "Pressair"[All Fields]) OR ("aclidinium bromide"[Supplementary Concept] OR "aclidinium bromide"[All Fields]) OR ("aclidinium bromide"[Supplementary Concept] OR "aclidinium bromide"[All Fields]) | 90 | (M3 muscarinic receptor) AND (1980:2020 [pdat]) | Tudorza Pressair | Aclidinium bromide | 2012 |
| drug20 | "axitinib"[MeSH Terms] OR "axitinib"[All Fields] OR "inlyta"[All Fields] OR "axitinib"[MeSH Terms] OR "axitinib"[All Fields] OR "axitinib"[MeSH Terms] OR "axitinib"[All Fields] | 153 | (receptors, vascular endothelial growth factor[MeSH Terms]) AND (1980:2020 [pdat]) | Inlyta | Axitinib | 2012 |
| drug200 | "vedolizumab"[Supplementary Concept] OR "vedolizumab"[All Fields] OR "entyvio"[All Fields] OR "vedolizumab"[Supplementary Concept] OR "vedolizumab"[All Fields] OR "vedolizumab"[Supplementary Concept] OR "vedolizumab"[All Fields] | 14 | (alpha4beta7 integrin) AND (1980:2020 [pdat]) | Entyvio | Vedolizumab | 2014 |
| drug201 | "velaglucerase alfa"[All Fields] OR "vpriv"[All Fields] OR ("Velaglucerase"[All Fields] AND "alfa"[All Fields]) OR ("Velaglucerase"[All Fields] AND "alfa"[All Fields]) | 29 | ("beta-glucosidase"[MeSH Terms] OR "beta-glucosidase"[All | Vpriv | Velaglucerase alfa | 2010 |

© 2023 Zhou EW et al. *JAMA Health Forum.*

| | | | | | | |
|---|---|---|---|---|---|---|
| | | | Fields]) AND (1980:2020 [pdat]) | | | |
| drug202 | "sofosbuvir"[MeSH Terms] OR "sofosbuvir"[All Fields] OR "sofosbuvir velpatasvir drug combination"[Supplementary Concept] OR "sofosbuvir velpatasvir drug combination"[All Fields] OR "epclusa"[All Fields] OR "velpatasvir"[Supplementary Concept] OR "velpatasvir"[All Fields] OR (("sofosbuvir"[MeSH Terms] OR "sofosbuvir"[All Fields]) AND ("velpatasvir"[Supplementary Concept] OR "velpatasvir"[All Fields])) OR (("sofosbuvir"[MeSH Terms] OR "sofosbuvir"[All Fields]) AND ("velpatasvir"[Supplementary Concept] OR "velpatasvir"[All Fields])) | 74 | (hcv ns5a) AND (1980:2020 [pdat]) | Epclusa | Sofosbuvir plus velpatasvir | 2016 |
| drug203 | "vemurafenib"[MeSH Terms] OR "vemurafenib"[All Fields] OR "zelboraf"[All Fields] OR "vemurafenib"[MeSH Terms] OR "vemurafenib"[All Fields] OR "vemurafenib"[MeSH Terms] OR "vemurafenib"[All Fields] | 32 | (braf) AND (1980:2020 [pdat]) | Zelboraf | Vemurafenib | 2011 |
| drug204 | "venetoclax"[Supplementary Concept] OR "venetoclax"[All Fields] OR "venclexta"[All Fields] OR "venetoclax"[Supplementary Concept] OR "venetoclax"[All Fields] OR "venetoclax"[Supplementary Concept] OR "venetoclax"[All Fields] | 23 | (Bcl-2) AND (1980:2020 [pdat]) | Venclexta | Venetoclax | 2016 |

© 2023 Zhou EW et al. *JAMA Health Forum.*

| | | | | | | |
|---|---|---|---|---|---|---|
| drug205 | (("breo"[All Fields] OR "fluticasone"[MeSH Terms] OR "fluticasone"[All Fields] OR "fluticason"[All Fields] OR "vilanterol"[Supplementary Concept] OR "vilanterol"[All Fields]) AND "Ellipta"[All Fields]) OR (("fluticasone"[MeSH Terms] OR "fluticasone"[All Fields] OR "fluticason"[All Fields]) AND ("vilanterol"[Supplementary Concept] OR "vilanterol"[All Fields])) OR (("fluticasone furoate"[Supplementary Concept] OR "fluticasone furoate"[All Fields]) AND ("vilanterol"[Supplementary Concept] OR "vilanterol"[All Fields])) | 28 | (beta-2-adrenergic receptor) AND (1980:2020 [pdat]) | Breo Ellipta | Fluticasone, vilanterol | 2013 |
| drug206 | "vilazodone hydrochloride"[MeSH Terms] OR ("vilazodone"[All Fields] AND "hydrochloride"[All Fields]) OR "vilazodone hydrochloride"[All Fields] OR "viibryd"[All Fields] OR "vilazodone"[All Fields] OR ("vilazodone hydrochloride"[MeSH Terms] OR ("vilazodone"[All Fields] AND "hydrochloride"[All Fields]) OR "vilazodone hydrochloride"[All Fields] OR "vilazodone"[All Fields]) OR ("vilazodone hydrochloride"[MeSH Terms] OR ("vilazodone"[All Fields] AND "hydrochloride"[All Fields]) OR "vilazodone hydrochloride"[All Fields] OR "vilazodone"[All Fields]) | 9 | (5-ht1a receptors OR serotonin reuptake) AND (1980:2020 [pdat]) | Viibryd | Vilazodone | 2011 |
| drug207 | "hhantag691"[Supplementary Concept] OR "hhantag691"[All Fields] OR "erivedge"[All Fields] OR "vismodegib"[All Fields] OR "hhantag691"[Supplementary Concept] OR "hhantag691"[All Fields] OR "vismodegib"[All Fields] OR "hhantag691"[Supplementary Concept] OR "hhantag691"[All Fields] OR "vismodegib"[All Fields] | 76 | (hedgehog signaling OR (hedgehog AND Drosophila)) AND (1980:2020 [pdat]) | Erivedge | Vismodegib | 2012 |

© 2023 Zhou EW et al. *JAMA Health Forum.*

| | | | | | | |
|---|---|---|---|---|---|---|
| drug208 | "vorapaxar"[Supplementary Concept] OR "vorapaxar"[All Fields] OR "zontivity"[All Fields] OR "vorapaxar s"[All Fields] OR "vorapaxar"[Supplementary Concept] OR "vorapaxar"[All Fields] OR "vorapaxar s"[All Fields] OR "vorapaxar"[Supplementary Concept] OR "vorapaxar"[All Fields] OR "vorapaxar s"[All Fields] | 119 | ("protease activated receptor") AND (1980:2020 [pdat]) | Zontivity | Vorapaxar | 2014 |
| drug209 | (("vortioxetine"[MeSH Terms] OR "vortioxetine"[All Fields] OR "brintellix"[All Fields]) AND ("trintellix"[All Fields] OR "vortioxetine"[MeSH Terms] OR "vortioxetine"[All Fields] OR "vortioxetine s"[All Fields])) OR ("vortioxetine"[MeSH Terms] OR "vortioxetine"[All Fields] OR "vortioxetine s"[All Fields]) OR ("vortioxetine"[MeSH Terms] OR "vortioxetine"[All Fields] OR "vortioxetine s"[All Fields]) | 132 | (serotonin receptor) AND (1980:2020 [pdat]) | Trintellix | Vortioxetine | 2013 |
| drug21 | "azilsartan"[Supplementary Concept] OR "azilsartan"[All Fields] OR "edarbi"[All Fields] OR "azilsartan"[Supplementary Concept] OR "azilsartan"[All Fields] OR "azilsartan medoxomil"[Supplementary Concept] OR "azilsartan medoxomil"[All Fields] | 18 | (angiotensin ii type 1 receptor) AND (1980:2020 [pdat]) | Edarbi | Azilsartan | 2011 |
| drug210 | "aflibercept"[Supplementary Concept] OR "aflibercept"[All Fields] OR "zaltrap"[All Fields] OR "ziv aflibercept"[All Fields] OR "aflibercept"[Supplementary Concept] OR "aflibercept"[All Fields] OR "ziv aflibercept"[All Fields] OR "aflibercept"[Supplementary Concept] OR "aflibercept"[All Fields] | 153 | (receptors, vascular endothelial growth factor[MeSH Terms]) AND (1980:2020 [pdat]) | Zaltrap | Ziv-aflibercept | 2012 |
| drug211 | "deferiprone"[MeSH Terms] OR "deferiprone"[All Fields] OR "ferriprox"[All Fields] OR ("deferiprone"[MeSH Terms] OR "deferiprone"[All Fields]) OR (("deferiprone"[MeSH Terms] OR "deferiprone"[All Fields]) AND "BTG"[All Fields]) | 0 | | Ferriprox | Deferiprone | 2011 |

© 2023 Zhou EW et al. *JAMA Health Forum.*

| | | | | | | |
|---|---|---|---|---|---|---|
| drug212 | "clobazam"[MeSH Terms] OR "clobazam"[All Fields] OR "onfi"[All Fields] OR ("clobazam"[MeSH Terms] OR "clobazam"[All Fields]) OR (("clobazam"[MeSH Terms] OR "clobazam"[All Fields]) AND "Lundbeck"[All Fields]) | 190 | (GABA-A receptor) AND (1980:2020 [pdat]) | Onfi | Clobazam | 2011 |
| drug213 | "filgrastim"[MeSH Terms] OR "filgrastim"[All Fields] OR ("tbo"[All Fields] AND "filgrastim"[All Fields]) OR "tbo filgrastim"[All Fields] OR (("filgrastim"[MeSH Terms] OR "filgrastim"[All Fields] OR "filgrastims"[All Fields]) AND "Teva"[All Fields]) | 220 | (Granulocyte-macrophage colony-stimulating factor) AND (1980:2020 [pdat]) | Neutroval | Tbo-Filgrastim | 2012 |
| drug214 | (("radium ra 223 dichloride"[Supplementary Concept] OR "radium ra 223 dichloride"[All Fields] OR "radium chloride ra 223"[All Fields] OR "xofigo"[All Fields]) AND "formerly"[All Fields] AND "Alpharadin"[All Fields]) OR ("radium ra 223 dichloride"[Supplementary Concept] OR "radium ra 223 dichloride"[All Fields] OR "radium ra 223 dichloride"[All Fields]) OR (("radium 223"[Supplementary Concept] OR "radium 223"[All Fields] OR "radium 223"[All Fields]) AND ("dichloride"[All Fields] OR "dichlorides"[All Fields])) | 242 | (hydroxyapatite) AND (1980:2020 [pdat]) | Xofigo | Radium Ra 223 dichloride | 2013 |
| drug215 | "daclizumab"[MeSH Terms] OR "daclizumab"[All Fields] OR "zinbryta"[All Fields] OR "daclizumab"[MeSH Terms] OR "daclizumab"[All Fields] OR "daclizumab hyp"[Supplementary Concept] OR "daclizumab hyp"[All Fields] OR "daclizumab high yield process"[All Fields] | 155 | (interleukin 2 receptor) AND (1980:2020 [pdat]) | Zinbryta | Daclizumab | 2016 |
| drug216 | "copanlisib"[Supplementary Concept] OR "copanlisib"[All Fields] OR "aliqopa"[All Fields] OR (("copanlisib"[Supplementary Concept] OR "copanlisib"[All Fields]) AND ("bisulfate"[All Fields] OR "bisulphate"[All Fields] OR "dihydrochloride"[All Fields] OR | 109 | (Phosphoinositide 3-kinase OR PI-3 kinase) AND (1980:2020 [pdat]) | Aliqopa | Copanlisib dihydrochloride | 2017 |

© 2023 Zhou EW et al. *JAMA Health Forum.*

| | | | | | | |
|---|---|---|---|---|---|---|
| | "dihydrochlorides"[All Fields] OR "sulfatated"[All Fields] OR "sulfatation"[All Fields] OR "sulfate s"[All Fields] OR "sulfated"[All Fields] OR "sulfates"[MeSH Terms] OR "sulfates"[All Fields] OR "sulfate"[All Fields] OR "sulphate"[All Fields] OR "sulfating"[All Fields] OR "sulfation"[All Fields] OR "sulfations"[All Fields] OR "sulphated"[All Fields] OR "sulphates"[All Fields] OR "sulphation"[All Fields])) OR ("copanlisib"[Supplementary Concept] OR "copanlisib"[All Fields]) | | | | | |
| drug217 | "brigatinib"[Supplementary Concept] OR "brigatinib"[All Fields] OR "alunbrig"[All Fields] OR "brigatinib"[Supplementary Concept] OR "brigatinib"[All Fields] OR "brigatinib"[Supplementary Concept] OR "brigatinib"[All Fields] | 123 | (receptor tyrosine kinase) AND (1980:2020 [pdat]) | Alunbrig | Brigatinib | 2017 |
| drug218 | "deutetrabenazine"[Supplementary Concept] OR "deutetrabenazine"[All Fields] OR "austedo"[All Fields] OR ("deutetrabenazine"[Supplementary Concept] OR "deutetrabenazine"[All Fields]) OR (("deuterate"[All Fields] OR "deuterated"[All Fields] OR "deuterating"[All Fields] OR "deuteration"[All Fields] OR "deuterations"[All Fields]) AND ("tetrabenazine"[MeSH Terms] OR "tetrabenazine"[All Fields]) AND "Auspex"[All Fields] AND ("biopharmaceutics"[MeSH Terms] OR "biopharmaceutics"[All Fields] OR "pharmaceutic"[All Fields] OR "pharmaceutics"[All Fields] OR | 165 | (monoamine transporter) AND (1980:2020 [pdat]) | Austedo | Deutetrabenazine | 2017 |

© 2023 Zhou EW et al. *JAMA Health Forum.*

| | | | | | | |
|---|---|---|---|---|---|---|
| | "pharmaceutical preparations"[MeSH Terms] OR ("pharmaceutical"[All Fields] AND "preparations"[All Fields]) OR "pharmaceutical preparations"[All Fields] OR "pharmaceutical"[All Fields] OR "pharmaceuticals"[All Fields] OR "pharmaceutical s"[All Fields] OR "pharmaceutically"[All Fields])) | | | | | |
| drug219 | "avelumab"[Supplementary Concept] OR "avelumab"[All Fields] OR "bavencio"[All Fields] OR "avelumab"[Supplementary Concept] OR "avelumab"[All Fields] OR "avelumab"[Supplementary Concept] OR "avelumab"[All Fields] | 116 | (programmed cell death-ligand 1 OR PDL1) AND (1980:2020 [pdat]) | Bavencio | Avelumab | 2017 |
| drug22 | "bazedoxifene"[Supplementary Concept] OR "bazedoxifene"[All Fields] OR "duavee"[All Fields] OR "estrogens conjugated usp"[All Fields] OR (("bazedoxifene"[Supplementary Concept] OR "bazedoxifene"[All Fields] OR "bazedoxifene acetate"[All Fields]) AND ("estrogen s"[All Fields] OR "estrogene"[All Fields] OR "estrogenes"[All Fields] OR "estrogenic"[All Fields] OR "estrogenically"[All Fields] OR "estrogenicities"[All Fields] OR "estrogenicity"[All Fields] OR "estrogenization"[All Fields] OR "estrogenized"[All Fields] OR "oestrogen"[All Fields] OR "estrogens"[Pharmacological Action] OR "estrogens"[MeSH Terms] OR "estrogens"[All Fields] OR "estrogen"[All | 62 | (estrogen receptor) AND (1980:2020 [pdat]) | Duavee | Bazedoxifene acetate plus oestrogens | 2013 |

© 2023 Zhou EW et al. *JAMA Health Forum.*

| | | | | | | |
|---|---|---|---|---|---|---|
| | Fields] OR "oestrogen s"[All Fields] OR "oestrogenic"[All Fields] OR "oestrogenically"[All Fields] OR "oestrogenicity"[All Fields] OR "oestrogenization"[All Fields] OR "oestrogens"[All Fields])) OR ("bazedoxifene"[Supplementary Concept] OR "bazedoxifene"[All Fields]) | | | | | |
| drug220 | "plazomicin"[Supplementary Concept] OR "plazomicin"[All Fields] OR "zemdri"[All Fields] OR "plazomicin"[Supplementary Concept] OR "plazomicin"[All Fields] OR "plazomicin"[Supplementary Concept] OR "plazomicin"[All Fields] | 243 | (DNA gyrase OR "topoisomerase IV") AND (1980:2020 [pdat]) | Baxdela | Delafloxacin | 2017 |
| drug221 | "triclabendazole"[MeSH Terms] OR "triclabendazole"[All Fields] OR "egaten"[All Fields] OR "triclabendazole"[MeSH Terms] OR "triclabendazole"[All Fields] OR "triclabendazole"[MeSH Terms] OR "triclabendazole"[All Fields] | 235 | (Trypanosma cruzi infection) AND (1980:2020 [pdat]) | Benznidazole | Benznidazole | 2017 |
| drug222 | "inotuzumab ozogamicin"[MeSH Terms] OR ("inotuzumab"[All Fields] AND "ozogamicin"[All Fields]) OR "inotuzumab ozogamicin"[All Fields] OR "besponsa"[All Fields] OR ("inotuzumab ozogamicin"[MeSH Terms] OR ("inotuzumab"[All Fields] AND "ozogamicin"[All Fields]) OR "inotuzumab ozogamicin"[All Fields]) OR ("inotuzumab ozogamicin"[MeSH Terms] OR ("inotuzumab"[All Fields] AND | 156 | (CD22) AND (1980:2020 [pdat]) | Besponsa | Inotuzumab ozogamicin | 2017 |

© 2023 Zhou EW et al. *JAMA Health Forum.*

| | | | | | | |
|---|---|---|---|---|---|---|
| | "ozogamicin"[All Fields]) OR "inotuzumab ozogamicin"[All Fields]) | | | | | |
| drug223 | "betrixaban"[Supplementary Concept] OR "betrixaban"[All Fields] OR "bevyxxa"[All Fields] OR "betrixaban"[Supplementary Concept] OR "betrixaban"[All Fields] OR "betrixaban"[Supplementary Concept] OR "betrixaban"[All Fields] | 63 | (factor Xa) AND (1980:2020 [pdat]) | Bevyxxa | Betrixaban | 2017 |
| drug224 | "cerliponase alfa"[Supplementary Concept] OR "cerliponase alfa"[All Fields] OR "brineura"[All Fields] OR "cerliponase"[All Fields] OR "cerliponase alfa"[Supplementary Concept] OR "cerliponase alfa"[All Fields] OR "cerliponase alfa"[Supplementary Concept] OR "cerliponase alfa"[All Fields] | 157 | (Tripeptidyl-peptidase) AND (1980:2020 [pdat]) | Brineura | Cerliponase alfa | 2017 |
| drug225 | "acalabrutinib"[Supplementary Concept] OR "acalabrutinib"[All Fields] OR "calquence"[All Fields] OR "acalabrutinib"[Supplementary Concept] OR "acalabrutinib"[All Fields] OR "acalabrutinib"[Supplementary Concept] OR "acalabrutinib"[All Fields] | 33 | (bruton's tyrosine kinase) AND (1980:2020 [pdat]) | Calquence | Acalabrutinib | 2017 |
| drug226 | "dupilumab"[Supplementary Concept] OR "dupilumab"[All Fields] OR "dupixent"[All Fields] OR "dupilumab"[Supplementary Concept] OR "dupilumab"[All Fields] OR "dupilumab"[Supplementary Concept] OR "dupilumab"[All Fields] | 227 | (interleukin 4 receptor) AND (1980:2020 [pdat]) | Dupixent | Dupilumab | 2017 |
| drug227 | "deflazacort"[Supplementary Concept] OR "deflazacort"[All Fields] OR "emflaza"[All Fields] OR ("deflazacort"[Supplementary Concept] OR "deflazacort"[All Fields]) OR (("deflazacort"[Supplementary Concept] OR "deflazacort"[All Fields]) AND ("marathon running"[MeSH Terms] OR ("marathon"[All Fields] AND "running"[All Fields]) OR | 169 | (glucocorticoid receptor OR NR3C1) AND (1980:2020 [pdat]) | Emflaza | Deflazacort | 2017 |

© 2023 Zhou EW et al. *JAMA Health Forum.*

| | | | | | | |
|---|---|---|---|---|---|---|
| | "marathon running"[All Fields] OR "marathon"[All Fields] OR "marathons"[All Fields] OR "marathoner"[All Fields] OR "marathoners"[All Fields]) AND ("biopharmaceutics"[MeSH Terms] OR "biopharmaceutics"[All Fields] OR "pharmaceutic"[All Fields] OR "pharmaceutics"[All Fields] OR "pharmaceutical preparations"[MeSH Terms] OR ("pharmaceutical"[All Fields] AND "preparations"[All Fields]) OR "pharmaceutical preparations"[All Fields] OR "pharmaceutical"[All Fields] OR "pharmaceuticals"[All Fields] OR "pharmaceutical s"[All Fields] OR "pharmaceutically"[All Fields])) | | | | | |
| drug228 | "benralizumab"[Supplementary Concept] OR "benralizumab"[All Fields] OR "fasenra"[All Fields] OR "benralizumab"[Supplementary Concept] OR "benralizumab"[All Fields] OR "benralizumab"[Supplementary Concept] OR "benralizumab"[All Fields] | 160 | (interleukin 5 receptor) AND (1980:2020 [pdat]) | Fasenra | Benralizumab | 2017 |
| drug229 | "giapreza"[Supplementary Concept] OR "giapreza"[All Fields] OR "giapreza"[All Fields] OR "angiotensin ii"[MeSH Terms] OR ("angiotensin"[All Fields] AND "ii"[All Fields]) OR "angiotensin ii"[All Fields] OR ("angiotensin ii"[MeSH Terms] OR ("angiotensin"[All Fields] | 18 | (angiotensin ii type 1 receptor) AND (1980:2020 [pdat]) | Giapreza | Angiotensin II, LFPC-501 | 2017 |

© 2023 Zhou EW et al. *JAMA Health Forum.*

| | | | | | | |
|---|---|---|---|---|---|---|
| | AND "ii"[All Fields]) OR "angiotensin ii"[All Fields]) OR "LJPC-501"[All Fields] | | | | | |
| drug23 | "bedaquiline"[Supplementary Concept] OR "bedaquiline"[All Fields] OR "sirturo"[All Fields] OR "bedaquiline"[Supplementary Concept] OR "bedaquiline"[All Fields] OR "bedaquiline"[Supplementary Concept] OR "bedaquiline"[All Fields] | 61 | (Escherichia coli ATP synthase) AND (1980:2020 [pdat]) | Sirturo | Bedaquiline | 2012 |
| drug230 | "emicizumab"[Supplementary Concept] OR "emicizumab"[All Fields] OR "hemlibra"[All Fields] OR "emicizumab"[Supplementary Concept] OR "emicizumab"[All Fields] OR "emicizumab"[Supplementary Concept] OR "emicizumab"[All Fields] | 230 | (Coagulation factor IX OR Coagulation factor X) AND (1980:2020 [pdat]) | Hemlibra | Emicizumab | 2017 |
| drug231 | "enasidenib"[Supplementary Concept] OR "enasidenib"[All Fields] OR "idhifa"[All Fields] OR (("enasidenib"[Supplementary Concept] OR "enasidenib"[All Fields]) AND ("mesyl"[All Fields] OR "mesylated"[All Fields] OR "mesylates"[MeSH Terms] OR "mesylates"[All Fields] OR "mesilate"[All Fields] OR "mesylate"[All Fields] OR "methanesulfonates"[All Fields] OR "mesylation"[All Fields] OR "methanesulfonic acid"[Supplementary Concept] OR "methanesulfonic acid"[All Fields] OR "methanesulfonate"[All Fields] OR "methanesulphonate"[All Fields] OR "methanesulphonates"[All Fields])) OR ("enasidenib"[Supplementary Concept] OR "enasidenib"[All Fields]) | 163 | (isocitrate dehydrogenase) AND (1980:2020 [pdat]) | Idhifa | Enasidenib mesylate | 2017 |

© 2023 Zhou EW et al. *JAMA Health Forum.*

| | | | | | | |
|---|---|---|---|---|---|---|
| drug232 | "durvalumab"[Supplementary Concept] OR "durvalumab"[All Fields] OR "imfinzi"[All Fields] OR "durvalumab"[Supplementary Concept] OR "durvalumab"[All Fields] OR "durvalumab"[Supplementary Concept] OR "durvalumab"[All Fields] | 116 | (programmed cell death-ligand 1 OR PDL1) AND (1980:2020 [pdat]) | Imfinzi | Durvalumab | 2017 |
| drug233 | "valbenazine"[Supplementary Concept] OR "valbenazine"[All Fields] OR "ingrezza"[All Fields] OR (("valbenazine"[Supplementary Concept] OR "valbenazine"[All Fields]) AND ("tosyl"[All Fields] OR "tosylate"[All Fields] OR "tosylated"[All Fields] OR "tosylates"[All Fields] OR "tosylation"[All Fields])) OR ("valbenazine"[Supplementary Concept] OR "valbenazine"[All Fields]) | 165 | (monoamine transporter) AND (1980:2020 [pdat]) | Ingrezza | Valbenazine tosylate | 2017 |
| drug234 | "sarilumab"[Supplementary Concept] OR "sarilumab"[All Fields] OR "kevzara"[All Fields] OR "sarilumab"[Supplementary Concept] OR "sarilumab"[All Fields] OR "sarilumab"[Supplementary Concept] OR "sarilumab"[All Fields] | 84 | (interleukin 6 receptor) AND (1980:2020 [pdat]) | Kevzara | Sarilumab | 2017 |
| drug235 | "ribociclib"[Supplementary Concept] OR "ribociclib"[All Fields] OR "kisqali"[All Fields] OR (("ribociclib"[Supplementary Concept] OR "ribociclib"[All Fields]) AND ("succinates"[MeSH Terms] OR "succinates"[All Fields] OR "succination"[All Fields] OR "succinic acid"[MeSH Terms] OR ("succinic"[All Fields] AND "acid"[All Fields]) OR "succinic acid"[All Fields] OR "succinate"[All Fields] OR "succinic"[All | 164 | ("cyclin-dependent kinase") AND (1980:2020 [pdat]) | Kisqali | Ribociclib succinate | 2017 |

© 2023 Zhou EW et al. *JAMA Health Forum.*

| | | | | | | |
|---|---|---|---|---|---|---|
| | Fields])) OR ("ribociclib"[Supplementary Concept] OR "ribociclib"[All Fields]) | | | | | |
| drug236 | "glecaprevir and pibrentasvir"[Supplementary Concept] OR "glecaprevir and pibrentasvir"[All Fields] OR "mavyret"[All Fields] OR "glecaprevir"[Supplementary Concept] OR "glecaprevir"[All Fields] OR "pibrentasvir"[Supplementary Concept] OR "pibrentasvir"[All Fields] OR "glecaprevir and pibrentasvir"[Supplementary Concept] OR "glecaprevir and pibrentasvir"[All Fields] OR "glecaprevir and pibrentasvir"[Supplementary Concept] OR "glecaprevir and pibrentasvir"[All Fields] | 225 | (NS3 protease OR Nonstructural protein 5A) AND (1980:2020 [pdat]) | Mavyret | Glecaprevir, pibrentasvir | 2017 |
| drug237 | "vestronidase alfa"[Supplementary Concept] OR "vestronidase alfa"[All Fields] OR "mepsevii"[All Fields] OR "vestronidase alfa"[Supplementary Concept] OR "vestronidase alfa"[All Fields] OR "vestronidase alfa vjbk"[All Fields] OR "vestronidase alfa"[Supplementary Concept] OR "vestronidase alfa"[All Fields] | 159 | (glucuronidase) AND (1980:2020 [pdat]) | Mepsevii | Vestronidase alfa-vjbk | 2017 |
| drug238 | "neratinib"[Supplementary Concept] OR "neratinib"[All Fields] OR "nerlynx"[All Fields] OR "neratinib"[Supplementary Concept] OR "neratinib"[All Fields] OR "neratinib maleate"[All Fields] OR "neratinib"[Supplementary Concept] OR "neratinib"[All Fields] | 187 | (Epidermal growth factor receptor) AND (1980:2020 [pdat]) | Nerlynx | Neratinib maleate | 2017 |
| drug239 | "ocrelizumab"[Supplementary Concept] OR "ocrelizumab"[All Fields] OR "ocrevus"[All Fields] OR "ocrelizumab"[Supplementary Concept] OR "ocrelizumab"[All Fields] OR "ocrelizumab"[Supplementary Concept] OR "ocrelizumab"[All Fields] | 37 | (CD20) AND (1980:2020 [pdat]) | Ocrevus | Ocrelizumab | 2017 |

© 2023 Zhou EW et al. *JAMA Health Forum.*

| | | | | | | |
|---|---|---|---|---|---|---|
| drug24 | "abatacept"[MeSH Terms] OR "abatacept"[All Fields] OR "belatacept"[All Fields] OR "nulojix"[All Fields] OR "abatacept"[MeSH Terms] OR "abatacept"[All Fields] OR "belatacept"[All Fields] OR "abatacept"[MeSH Terms] OR "abatacept"[All Fields] OR "belatacept"[All Fields] | 231 | (CD80 OR CD86) AND (1980:2020 [pdat]) | Nulojix | Belatacept | 2011 |
| drug240 | "semaglutide"[Supplementary Concept] OR "semaglutide"[All Fields] OR "ozempic"[All Fields] OR "semaglutide"[Supplementary Concept] OR "semaglutide"[All Fields] OR "semaglutide"[Supplementary Concept] OR "semaglutide"[All Fields] | 67 | (glucagon-like peptide 1) AND (1980:2020 [pdat]) | Ozempic | Semaglutide | 2017 |
| drug241 | "etelcalcetide hydrochloride"[Supplementary Concept] OR "etelcalcetide hydrochloride"[All Fields] OR "etelcalcetide"[All Fields] OR "parsabiv"[All Fields] OR "etelcalcetide hydrochloride"[Supplementary Concept] OR "etelcalcetide hydrochloride"[All Fields] OR "etelcalcetide"[All Fields] OR "etelcalcetide hydrochloride"[Supplementary Concept] OR "etelcalcetide hydrochloride"[All Fields] OR "velcalcetide"[All Fields] | 168 | ("calcium sensing receptor") AND (1980:2020 [pdat]) | Parsabiv | Etelcalcetide | 2017 |
| drug242 | "letermovir"[Supplementary Concept] OR "letermovir"[All Fields] OR "prevymis"[All Fields] OR "letermovir"[Supplementary Concept] OR "letermovir"[All Fields] OR "letermovir"[Supplementary Concept] OR "letermovir"[All Fields] | 0 | | Prevymis | Letermovir | 2017 |
| drug243 | "edaravone"[MeSH Terms] OR "edaravone"[All Fields] OR "radicava"[All Fields] OR "edaravone"[MeSH Terms] OR "edaravone"[All Fields] OR "edaravone"[MeSH Terms] OR "edaravone"[All Fields] | 0 | | Radicava | Edaravone | 2017 |

© 2023 Zhou EW et al. *JAMA Health Forum.*

| | | | | | | |
|---|---|---|---|---|---|---|
| drug244 | "netarsudil"[Supplementary Concept] OR "netarsudil"[All Fields] OR "rhopressa"[All Fields] OR "netarsudil"[Supplementary Concept] OR "netarsudil"[All Fields] OR "netarsudil"[Supplementary Concept] OR "netarsudil"[All Fields] | 170 | (Rho-associated protein kinase) AND (1980:2020 [pdat]) | Rhopressa | Netarsudil | 2017 |
| drug245 | "midostaurin"[Supplementary Concept] OR "midostaurin"[All Fields] OR "rydapt"[All Fields] OR "midostaurin s"[All Fields] OR "midostaurin"[Supplementary Concept] OR "midostaurin"[All Fields] OR "midostaurin s"[All Fields] OR "midostaurin"[Supplementary Concept] OR "midostaurin"[All Fields] OR "midostaurin s"[All Fields] | 123 | (receptor tyrosine kinase) AND (1980:2020 [pdat]) | Rydapt | Midostaurin | 2017 |
| drug246 | "brodalumab"[Supplementary Concept] OR "brodalumab"[All Fields] OR "siliq"[All Fields] OR "brodalumab"[Supplementary Concept] OR "brodalumab"[All Fields] OR "brodalumab"[Supplementary Concept] OR "brodalumab"[All Fields] | 82 | (interleukin 17 receptor) AND (1980:2020 [pdat]) | Siliq | Brodalumab | 2017 |
| drug247 | "lefamulin"[Supplementary Concept] OR "lefamulin"[All Fields] OR "xenleta"[All Fields] OR "lefamulin"[Supplementary Concept] OR "lefamulin"[All Fields] OR "lefamulin"[Supplementary Concept] OR "lefamulin"[All Fields] | 0 | | Solosec | Secnidazole | 2017 |
| drug248 | "ertugliflozin"[Supplementary Concept] OR "ertugliflozin"[All Fields] OR "steglatro"[All Fields] OR "ertugliflozin"[Supplementary Concept] OR "ertugliflozin"[All Fields] OR "ertugliflozin"[Supplementary Concept] OR "ertugliflozin"[All Fields] | 135 | (sodium glucose transporter) AND (1980:2020 [pdat]) | Steglatro | Ertugliflozin | 2017 |
| drug249 | "naldemedine"[Supplementary Concept] OR "naldemedine"[All Fields] OR "symproic"[All Fields] OR "naldemedine"[Supplementary Concept] OR "naldemedine"[All Fields] OR "naldemedine"[Supplementary Concept] OR "naldemedine"[All Fields] | 128 | (receptors, opioid[MeSH Terms]) AND (1980:2020 [pdat]) | Symproic | Naldemedine | 2017 |

© 2023 Zhou EW et al. *JAMA Health Forum.*

| | | | | | | |
|---|---|---|---|---|---|---|
| drug25 | "belimumab"[Supplementary Concept] OR "belimumab"[All Fields] OR "benlysta"[All Fields] OR "belimumab"[Supplementary Concept] OR "belimumab"[All Fields] OR "belimumab"[Supplementary Concept] OR "belimumab"[All Fields] | 145 | (TNFSF13B) AND (1980:2020 [pdat]) | Benlysta | Belimumab | 2011 |
| drug250 | "guselkumab"[Supplementary Concept] OR "guselkumab"[All Fields] OR "tremfya"[All Fields] OR "guselkumab"[Supplementary Concept] OR "guselkumab"[All Fields] OR "guselkumab"[Supplementary Concept] OR "guselkumab"[All Fields] | 158 | (interleukin 23) AND (1980:2020 [pdat]) | Tremfya | Guselkumab | 2017 |
| drug251 | "plecanatide"[Supplementary Concept] OR "plecanatide"[All Fields] OR "trulance"[All Fields] OR "plecanatide"[Supplementary Concept] OR "plecanatide"[All Fields] OR "plecanatide"[Supplementary Concept] OR "plecanatide"[All Fields] | 69 | (guanylyl cyclase c) AND (1980:2020 [pdat]) | Trulance | Plecanatide | 2017 |
| drug252 | "abaloparatide"[Supplementary Concept] OR "abaloparatide"[All Fields] OR "tymlos"[All Fields] OR "abaloparatide"[Supplementary Concept] OR "abaloparatide"[All Fields] OR "abaloparatide"[Supplementary Concept] OR "abaloparatide"[All Fields] | 104 | (parathyroid hormone receptor) AND (1980:2020 [pdat]) | Tymlos | Abaloparatide | 2017 |
| drug253 | "cefiderocol"[Supplementary Concept] OR "cefiderocol"[All Fields] OR "fetroja"[All Fields] OR (("cefiderocol"[Supplementary Concept] OR "cefiderocol"[All Fields]) AND ("bisulfate"[All Fields] OR "bisulphate"[All Fields] OR "dihydrochloride"[All Fields] OR "dihydrochlorides"[All Fields] OR "sulfatated"[All Fields] OR "sulfatation"[All Fields] OR "sulfate s"[All Fields] OR "sulfated"[All Fields] OR "sulfates"[MeSH Terms] OR "sulfates"[All Fields] OR "sulfate"[All Fields] OR "sulphate"[All Fields] OR "sulfating"[All Fields] OR "sulfation"[All Fields] OR "sulfations"[All Fields] OR "sulphated"[All Fields] OR "sulphates"[All Fields] OR "sulphation"[All Fields]) AND | 246 | (beta lactamases) AND (1980:2020 [pdat]) | Vabomere | Meropenem; vaborbactam | 2017 |

© 2023 Zhou EW et al. *JAMA Health Forum.*

| | | | | | | |
|---|---|---|---|---|---|---|
| | ("tosyl"[All Fields] OR "tosylate"[All Fields] OR "tosylated"[All Fields] OR "tosylates"[All Fields] OR "tosylation"[All Fields])) OR ("cefiderocol"[Supplementary Concept] OR "cefiderocol"[All Fields]) | | | | | |
| drug254 | "abemaciclib"[Supplementary Concept] OR "abemaciclib"[All Fields] OR "verzenio"[All Fields] OR "abemaciclib"[Supplementary Concept] OR "abemaciclib"[All Fields] OR "abemaciclib"[Supplementary Concept] OR "abemaciclib"[All Fields] | 164 | ("cyclin-dependent kinase") AND (1980:2020 [pdat]) | Verzenio | Abemaciclib | 2017 |
| drug255 | "sofosbuvir velpatasvir voxilaprevir drug combination"[Supplementary Concept] OR "sofosbuvir velpatasvir voxilaprevir drug combination"[All Fields] OR "vosevi"[All Fields] OR "sofosbuvir"[MeSH Terms] OR "sofosbuvir"[All Fields] OR "velpatasvir"[Supplementary Concept] OR "velpatasvir"[All Fields] OR "voxilaprevir"[Supplementary Concept] OR "voxilaprevir"[All Fields] OR (("sofosbuvir"[MeSH Terms] OR "sofosbuvir"[All Fields]) AND ("velpatasvir"[Supplementary Concept] OR "velpatasvir"[All Fields]) AND ("voxilaprevir"[Supplementary Concept] OR "voxilaprevir"[All Fields])) OR (("sofosbuvir"[MeSH Terms] OR "sofosbuvir"[All Fields]) AND ("velpatasvir"[Supplementary Concept] OR | 161 | (Hepatitis C virus protease) AND (1980:2020 [pdat]) | Vosevi | Sofosbuvir, velpatasvir, voxilaprevir | 2017 |

© 2023 Zhou EW et al. *JAMA Health Forum.*

| | | | | | | |
|---|---|---|---|---|---|---|
| | "velpatasvir"[All Fields]) AND ("voxilaprevir"[Supplementary Concept] OR "voxilaprevir"[All Fields])) | | | | | |
| drug256 | "bol 303259 x"[Supplementary Concept] OR "bol 303259 x"[All Fields] OR "latanoprostene bunod"[All Fields] OR "vyzulta"[All Fields] OR "bol 303259 x"[Supplementary Concept] OR "bol 303259 x"[All Fields] OR "latanoprostene bunod"[All Fields] OR "bol 303259 x"[Supplementary Concept] OR "bol 303259 x"[All Fields] OR "latanoprostene bunod"[All Fields] | 118 | (Prostaglandin F receptor) AND (1980:2020 [pdat]) | Vyzulta | Latanoprostene bunod | 2017 |
| drug257 | "safinamide"[Supplementary Concept] OR "safinamide"[All Fields] OR "xadago"[All Fields] OR ("safinamide"[Supplementary Concept] OR "safinamide"[All Fields]) OR (("safinamide"[Supplementary Concept] OR "safinamide"[All Fields]) AND ("mesyl"[All Fields] OR "mesylated"[All Fields] OR "mesylates"[MeSH Terms] OR "mesylates"[All Fields] OR "mesilate"[All Fields] OR "mesylate"[All Fields] OR "methanesulfonates"[All Fields] OR "mesylation"[All Fields] OR "methanesulfonic acid"[Supplementary Concept] OR "methanesulfonic acid"[All Fields] OR "methanesulfonate"[All Fields] OR "methanesulphonate"[All Fields] OR "methanesulphonates"[All Fields])) | 166 | (monoamine oxidase B) AND (1980:2020 [pdat]) | Xadago | Safinamide | 2017 |

© 2023 Zhou EW et al. *JAMA Health Forum.*

| | | | | | | |
|---|---|---|---|---|---|---|
| drug258 | "cilastatin"[MeSH Terms] OR "cilastatin"[All Fields] OR "imipenem"[MeSH Terms] OR "imipenem"[All Fields] OR "imipenem s"[All Fields] OR "imipenem cilastatin and relebactam"[Supplementary Concept] OR "imipenem cilastatin and relebactam"[All Fields] OR "recarbrio"[All Fields] OR "imipeneme"[All Fields] OR "relebactam"[Supplementary Concept] OR "relebactam"[All Fields] OR (("cilastatin"[MeSH Terms] OR "cilastatin"[All Fields] OR ("cilastatin"[All Fields] AND "sodium"[All Fields]) OR "cilastatin sodium"[All Fields]) AND ("imipenem"[MeSH Terms] OR "imipenem"[All Fields] OR "imipenem s"[All Fields] OR "imipeneme"[All Fields]) AND ("relebactam"[Supplementary Concept] OR "relebactam"[All Fields])) OR (("cilastatin"[MeSH Terms] OR "cilastatin"[All Fields]) AND ("imipenem"[MeSH Terms] OR "imipenem"[All Fields] OR "imipenem s"[All Fields] OR "imipeneme"[All Fields]) AND ("relebactam"[Supplementary Concept] OR "relebactam"[All Fields])) | 243 | (DNA gyrase OR "topoisomerase IV") AND (1980:2020 [pdat]) | Xepi | Ozenoxacin | 2017 |

© 2023 Zhou EW et al. *JAMA Health Forum.*

| | | | | | | |
|---|---|---|---|---|---|---|
| drug259 | "telotristat ethyl"[Supplementary Concept] OR "telotristat ethyl"[All Fields] OR "xermelo"[All Fields] OR "telotristat"[Supplementary Concept] OR "telotristat"[All Fields] OR "telotristat etiprate"[All Fields] OR "telotristat"[Supplementary Concept] OR "telotristat"[All Fields] | 167 | (Tryptophan hydroxylase) AND (1980:2020 [pdat]) | Xermelo | Telotristat etiprate | 2017 |
| drug26 | "beleodaq"[All Fields] OR "belinostat"[Supplementary Concept] OR "belinostat"[All Fields] OR "belinostat"[Supplementary Concept] OR "belinostat"[All Fields] OR "belinostat"[Supplementary Concept] OR "belinostat"[All Fields] | 78 | (histone deacetylases[MeSH Terms]) AND (1980:2020 [pdat]) | Beleodaq | Belinostat | 2014 |
| drug260 | "niraparib"[Supplementary Concept] OR "niraparib"[All Fields] OR "zejula"[All Fields] OR "niraparib"[Supplementary Concept] OR "niraparib"[All Fields] OR "niraparib"[Supplementary Concept] OR "niraparib"[All Fields] | 105 | (PARP) AND (1980:2020 [pdat]) | Zejula | Niraparib | 2017 |
| drug261 | "botulinum toxins, type a"[MeSH Terms] OR "type a botulinum toxins"[All Fields] OR "botulinum toxin type a"[All Fields] OR "prabotulinumtoxin a"[Supplementary Concept] OR "prabotulinumtoxin a"[All Fields] OR "jeuveau"[All Fields] OR ("prabotulinumtoxin a"[Supplementary Concept] OR "prabotulinumtoxin a"[All Fields] OR "prabotulinumtoxina xvfs"[All Fields]) OR | 247 | (bacterial rna polymerase) AND (1980:2020 [pdat]) | Aemcolo | Rifamycin | 2018 |

© 2023 Zhou EW et al. *JAMA Health Forum.*

| | | | | | | |
|---|---|---|---|---|---|---|
| | ("Prabotulinum"[All Fields] AND ("toxin s"[All Fields] OR "toxine"[All Fields] OR "toxins, biological"[MeSH Terms] OR ("toxins"[All Fields] AND "biological"[All Fields]) OR "biological toxins"[All Fields] OR "toxin"[All Fields] OR "toxins"[All Fields])) | | | | | |
| drug262 | "erenumab"[Supplementary Concept] OR "erenumab"[All Fields] OR "aimovig"[All Fields] OR "erenumab"[Supplementary Concept] OR "erenumab"[All Fields] OR "erenumab"[Supplementary Concept] OR "erenumab"[All Fields] | 177 | (calcitonin gene-related peptide receptor) AND (1980:2020 [pdat]) | Aimovig | Erenumab | 2018 |
| drug263 | "erenumab"[Supplementary Concept] OR "erenumab"[All Fields] OR "ajovy"[All Fields] OR "fremanezumab"[Supplementary Concept] OR "fremanezumab"[All Fields] OR "fremanezumab"[Supplementary Concept] OR "fremanezumab"[All Fields] OR "fremanezumab"[Supplementary Concept] OR "fremanezumab"[All Fields] | 178 | (calcitonin gene-related peptide) AND (1980:2020 [pdat]) | Ajovy | Fremanezumab | 2018 |
| drug264 | "factor xiii"[MeSH Terms] OR ("factor"[All Fields] AND "xiii"[All Fields]) OR "factor xiii"[All Fields] OR "tretten"[All Fields] OR ((("factor xiii"[MeSH Terms] OR ("factor"[All Fields] AND "xiii"[All Fields]) OR "factor xiii"[All Fields] OR ("coagulation"[All Fields] AND "factor"[All Fields] AND "xiii"[All Fields]) OR "coagulation factor xiii"[All Fields]) AND ("protein subunits"[MeSH Terms] OR ("protein"[All Fields] AND "subunits"[All Fields]) OR "protein subunits"[All Fields] OR "subunit"[All Fields] OR "subunit s"[All Fields] OR "subunits"[All Fields])) AND ("f8 protein human"[Supplementary Concept] OR "f8 protein human"[All Fields] OR | 8 | ((substance p receptor) OR tachykinin receptor 1) AND (1980:2020 [pdat]) | Akynzeo IV | Palonosetron, fosnetupitant | 2018 |

© 2023 Zhou EW et al. *JAMA Health Forum.*

| | | | | | | |
|---|---|---|---|---|---|---|
| | "recombinate"[All Fields] OR "recombinant"[All Fields] OR "recombinants"[All Fields] OR "recombinated"[All Fields] OR "recombinates"[All Fields] OR "recombination, genetic"[MeSH Terms] OR ("recombination"[All Fields] AND "genetic"[All Fields]) OR "genetic recombination"[All Fields] OR "recombination"[All Fields] OR "recombinations"[All Fields] OR "recombinational"[All Fields] OR "recombinative"[All Fields] OR "recombine"[All Fields] OR "recombined"[All Fields] OR "recombineered"[All Fields] OR "recombineering"[All Fields] OR "recombines"[All Fields] OR "recombining"[All Fields])) OR "catridecacog"[All Fields] | | | | | |
| drug265 | "ethinyl oestradiol"[All Fields] OR "ethinyl estradiol"[MeSH Terms] OR ("ethinyl"[All Fields] AND "estradiol"[All Fields]) OR "ethinyl estradiol"[All Fields] OR "segesterone acetate and ethinyl estradiol vaginal system"[Supplementary Concept] OR "segesterone acetate and ethinyl estradiol vaginal system"[All Fields] OR "annovera"[All Fields] OR "segesterone"[All Fields] OR ("segesterone acetate and ethinyl estradiol vaginal system"[Supplementary Concept] OR "segesterone acetate and ethinyl estradiol vaginal system"[All Fields]) OR (("st 1435"[Supplementary Concept] OR "st 1435"[All Fields] OR "nestorone"[All Fields]) AND ("ethinyl estradiol"[MeSH Terms] OR ("ethinyl"[All Fields] AND "estradiol"[All Fields]) OR "ethinyl estradiol"[All Fields] OR "ethinylestradiol"[All Fields] OR "ethinyloestradiol"[All Fields]) AND ("contraceptive devices, female"[MeSH Terms] OR ("contraceptive"[All Fields] AND "devices"[All Fields] AND "female"[All Fields]) OR "female contraceptive devices"[All Fields] | 114 | (progesterone receptor[MeSH Terms]) AND (1980:2020 [pdat]) | Annovera | Segesterone acetate and ethinyl estradiol vaginal system | 2018 |

© 2023 Zhou EW et al. *JAMA Health Forum.*

| | | | | | | |
|---|---|---|---|---|---|---|
| | OR ("vaginal"[All Fields] AND "ring"[All Fields]) OR "vaginal ring"[All Fields]) AND ("populate"[All Fields] OR "populated"[All Fields] OR "populates"[All Fields] OR "populating"[All Fields] OR "population"[MeSH Terms] OR "population"[All Fields] OR "population groups"[MeSH Terms] OR ("population"[All Fields] AND "groups"[All Fields]) OR "population groups"[All Fields] OR "populations"[All Fields] OR "population s"[All Fields] OR "populational"[All Fields] OR "populous"[All Fields]) AND ("council"[All Fields] OR "council s"[All Fields] OR "councils"[All Fields])) | | | | | |

© 2023 Zhou EW et al. *JAMA Health Forum.*

| | | | | | | |
|---|---|---|---|---|---|---|
| drug266 | "calaspargase pegol"[Supplementary Concept] OR "calaspargase pegol"[All Fields] OR "asparlas"[All Fields] OR "calaspargase pegol"[Supplementary Concept] OR "calaspargase pegol"[All Fields] OR "calaspargase pegol"[Supplementary Concept] OR "calaspargase pegol"[All Fields] | 173 | ("asparagine"[MeSH Terms]) AND (1980:2020 [pdat]) | Asparlas | Calaspargase pegol | 2018 |
| drug267 | "racivir"[Supplementary Concept] OR "racivir"[All Fields] OR "emtricitabine"[All Fields] OR "emtricitabine"[MeSH Terms] OR "bictegravir"[Supplementary Concept] OR "bictegravir"[All Fields] OR "bictegravir embitcitabine tenofovir alafenamide drug combination"[Supplementary Concept] OR "bictegravir embitcitabine tenofovir alafenamide drug combination"[All Fields] OR "biktarvy"[All Fields] OR "emtricitabin"[All Fields] OR "tenofovir alafenamide"[All Fields] | 183 | (HIV integrase) AND (1980:2020 [pdat]) | Biktarvy | Bictegravir, emtricitabine, tenofovir alafenamide | 2018 |

© 2023 Zhou EW et al. *JAMA Health Forum.*

| | | | | | | |
|---|---|---|---|---|---|---|
| | OR (("bictegravir"[Supplementary Concept] OR "bictegravir"[All Fields]) AND ("racivir"[Supplementary Concept] OR "racivir"[All Fields] OR "emtricitabine"[All Fields] OR "emtricitabine"[MeSH Terms] OR "emtricitabin"[All Fields]) AND ("tenofovir"[MeSH Terms] OR "tenofovir"[All Fields]) AND "alafenamide"[All Fields]) OR (("bictegravir"[Supplementary Concept] OR "bictegravir"[All Fields]) AND "emtricitibine"[All Fields] AND ("tenofovir"[MeSH Terms] OR "tenofovir"[All Fields]) AND "alafenamide"[All Fields] AND ("gilead"[All Fields] OR "gilead s"[All Fields]) AND ("science"[MeSH Terms] OR "science"[All Fields] OR "sciences"[All Fields] OR "science s"[All Fields] OR "sciencing"[All Fields])) | | | | | |
| drug268 | "encorafenib"[Supplementary Concept] OR "encorafenib"[All Fields] OR "braftovi"[All Fields] OR "encorafenib"[Supplementary Concept] OR "encorafenib"[All Fields] OR "encorafenib"[Supplementary Concept] OR "encorafenib"[All Fields] | 32 | (braf) AND (1980:2020 [pdat]) | Braftovi | Encorafenib | 2018 |

© 2023 Zhou EW et al. *JAMA Health Forum.*

| | | | | | | |
|---|---|---|---|---|---|---|
| drug269 | "duvelisib"[Supplementary Concept] OR "duvelisib"[All Fields] OR "copiktra"[All Fields] OR "duvelisib"[Supplementary Concept] OR "duvelisib"[All Fields] OR "duvelisib"[Supplementary Concept] OR "duvelisib"[All Fields] | 109 | (Phosphoinositide 3-kinase OR PI-3 kinase) AND (1980:2020 [pdat]) | Copiktra | Duvelisib | 2018 |
| drug27 | "bezlotoxumab"[Supplementary Concept] OR "bezlotoxumab"[All Fields] OR "zinplava"[All Fields] OR "bezlotoxumab"[Supplementary Concept] OR "bezlotoxumab"[All Fields] OR "bezlotoxumab"[Supplementary Concept] OR "bezlotoxumab"[All Fields] | 47 | (clostridium difficile toxin b) AND (1980:2020 [pdat]) | Zinplava | Bezlotoxumab | 2016 |
| drug270 | "burosumab"[Supplementary Concept] OR "burosumab"[All Fields] OR "crysvita"[All Fields] OR "burosumab"[Supplementary Concept] OR "burosumab"[All Fields] OR "burosumab"[Supplementary Concept] OR "burosumab"[All Fields] | 181 | (Fibroblast growth factor 23 OR FGF23) AND (1980:2020 [pdat]) | Crysvita | Burosumab | 2018 |
| drug271 | "glasdegib"[Supplementary Concept] OR "glasdegib"[All Fields] OR "daurismo"[All Fields] OR "glasdegib"[Supplementary Concept] OR "glasdegib"[All Fields] OR "glasdegib"[Supplementary Concept] OR "glasdegib"[All Fields] | 76 | (hedgehog signaling OR (hedgehog AND Drosophila)) AND (1980:2020 [pdat]) | Daurismo | Glasdegib | 2018 |
| drug272 | "stiripentol"[Supplementary Concept] OR "stiripentol"[All Fields] OR "diacomit"[All Fields] OR "stiripentol"[Supplementary Concept] OR "stiripentol"[All Fields] OR "stiripentol"[Supplementary Concept] OR "stiripentol"[All Fields] | 190 | (GABA-A receptor) AND (1980:2020 [pdat]) | Diacomit | Stiripentol | 2018 |
| drug273 | "avatrombopag"[Supplementary Concept] OR "avatrombopag"[All Fields] OR "doptelet"[All Fields] OR "avatrombopag"[Supplementary Concept] OR "avatrombopag"[All Fields] OR "avatrombopag"[Supplementary Concept] OR "avatrombopag"[All Fields] | 188 | (Thrombopoietin receptor) AND (1980:2020 [pdat]) | Doptelet | Avatrombopag | 2018 |

© 2023 Zhou EW et al. *JAMA Health Forum.*

| | | | | | | |
|---|---|---|---|---|---|---|
| drug274 | "tagraxofusp"[Supplementary Concept] OR "tagraxofusp"[All Fields] OR "elzonris"[All Fields] OR "tagraxofusp"[Supplementary Concept] OR "tagraxofusp"[All Fields] OR "tagraxofusp"[Supplementary Concept] OR "tagraxofusp"[All Fields] | 174 | (CD123) AND (1980:2020 [pdat]) | Elzonris | Tagraxofusp | 2018 |
| drug275 | "erenumab"[Supplementary Concept] OR "erenumab"[All Fields] OR "emgality"[All Fields] OR "galcanezumab"[Supplementary Concept] OR "galcanezumab"[All Fields] OR "galcanezumab"[Supplementary Concept] OR "galcanezumab"[All Fields] OR "galcanezumab"[Supplementary Concept] OR "galcanezumab"[All Fields] | 178 | (calcitonin gene-related peptide) AND (1980:2020 [pdat]) | Emgality | Galcanezumab | 2018 |
| drug276 | "cannabidiol"[MeSH Terms] OR "cannabidiol"[All Fields] OR "epidiolex"[All Fields] OR "cannabidiolic"[All Fields] OR ("cannabidiol"[MeSH Terms] OR "cannabidiol"[All Fields] OR "cannabidiolic"[All Fields]) OR (("cannabidiol"[MeSH Terms] OR "cannabidiol"[All Fields] OR "cannabidiolic"[All Fields]) AND ("ground water"[Journal] OR "gw"[All Fields]) AND ("biopharmaceutics"[MeSH Terms] OR "biopharmaceutics"[All Fields] OR "pharmaceutic"[All Fields] OR "pharmaceutics"[All Fields] OR "pharmaceutical preparations"[MeSH Terms] OR ("pharmaceutical"[All Fields] AND "preparations"[All Fields]) OR "pharmaceutical preparations"[All Fields] OR "pharmaceutical"[All Fields] OR "pharmaceuticals"[All Fields] OR "pharmaceutical s"[All Fields] OR "pharmaceutically"[All Fields])) | 0 | | Epidiolex | cannabidiol | 2018 |

© 2023 Zhou EW et al. *JAMA Health Forum.*

| | | | | | | |
|---|---|---|---|---|---|---|
| drug277 | "apalutamide"[Supplementary Concept] OR "apalutamide"[All Fields] OR "erleada"[All Fields] OR "apalutamide"[Supplementary Concept] OR "apalutamide"[All Fields] OR "apalutamide"[Supplementary Concept] OR "apalutamide"[All Fields] | 17 | (androgen receptor) AND (1980:2020 [pdat]) | Erleada | Apalutamide | 2018 |
| drug278 | "amifampridine"[MeSH Terms] OR "amifampridine"[All Fields] OR "firdapse"[All Fields] OR ("amifampridine"[MeSH Terms] OR "amifampridine"[All Fields]) OR ("amifampridine"[MeSH Terms] OR "amifampridine"[All Fields] OR ("amifampridine"[All Fields] AND "phosphate"[All Fields]) OR "amifampridine phosphate"[All Fields]) | 112 | (potassium channel) AND (1980:2020 [pdat]) | Firdapse | Amifampridine | 2018 |
| drug279 | "migalastat"[Supplementary Concept] OR "migalastat"[All Fields] OR "galafold"[All Fields] OR "migalastat"[Supplementary Concept] OR "migalastat"[All Fields] OR "migalastat"[Supplementary Concept] OR "migalastat"[All Fields] OR "migalastat hydrochloride"[All Fields] | 194 | (alpha-galactosidase) AND (1980:2020 [pdat]) | Galafold | Migalastat | 2018 |
| drug28 | "blinatumomab"[Supplementary Concept] OR "blinatumomab"[All Fields] OR "blincyto"[All Fields] OR "blinatumomab"[Supplementary Concept] OR "blinatumomab"[All Fields] OR "blinatumomab"[Supplementary Concept] OR "blinatumomab"[All Fields] | 38 | (CD3) AND (1980:2020 [pdat]) | Blincyto | Blinatumomab | 2014 |

© 2023 Zhou EW et al. *JAMA Health Forum.*

| | | | | | | |
|---|---|---|---|---|---|---|
| drug280 | "emapalumab"[Supplementary Concept] OR "emapalumab"[All Fields] OR "emapalumab"[All Fields] OR "gamifant"[All Fields] OR "emapalumab"[Supplementary Concept] OR "emapalumab"[All Fields] OR "emapalumab"[All Fields] OR "emapalumab"[Supplementary Concept] OR "emapalumab"[All Fields] OR "emapalumab"[All Fields] | 179 | (interferon gamma) AND (1980:2020 [pdat]) | Gamifant | Emapalumab | 2018 |
| drug281 | "tildrakizumab"[Supplementary Concept] OR "tildrakizumab"[All Fields] OR "ilumya"[All Fields] OR "tildrakizumab"[Supplementary Concept] OR "tildrakizumab"[All Fields] OR "tildrakizumab"[Supplementary Concept] OR "tildrakizumab"[All Fields] | 158 | (interleukin 23) AND (1980:2020 [pdat]) | Ilumya | Tildrakizumab | 2018 |
| drug282 | "tafenoquine"[Supplementary Concept] OR "tafenoquine"[All Fields] OR "krintafel"[All Fields] OR "tafenoquine"[Supplementary Concept] OR "tafenoquine"[All Fields] OR "tafenoquine"[Supplementary Concept] OR "tafenoquine"[All Fields] | 0 | | Krintafel | Tafenoquine | 2018 |
| drug283 | "cemiplimab"[Supplementary Concept] OR "cemiplimab"[All Fields] OR "libtayo"[All Fields] OR "cemiplimab"[Supplementary Concept] OR "cemiplimab"[All Fields] OR "cemiplimab"[Supplementary Concept] OR "cemiplimab"[All Fields] | 115 | ("programmed cell death 1") AND (1980:2020 [pdat]) | Libtayo | Cemiplimab | 2018 |
| drug284 | "factor viii"[MeSH Terms] OR ("factor"[All Fields] AND "viii"[All Fields]) OR "factor viii"[All Fields] OR "nuwiq"[All Fields] OR ("Simoctocog"[All Fields] AND "alfa"[All Fields]) OR ("Simoctocog"[All Fields] AND "alfa"[All Fields]) | 237 | (potassium AND hyperkalemia) AND (1980:2020 [pdat]) | Lokelma | Sodium zirconium cyclosilicate | 2018 |
| drug285 | "lorlatinib"[Supplementary Concept] OR "lorlatinib"[All Fields] OR "lorbrena"[All Fields] OR "lorlatinib"[Supplementary Concept] OR "lorlatinib"[All Fields] OR "lorlatinib"[Supplementary Concept] OR "lorlatinib"[All Fields] | 185 | (ALK tyrosine kinase receptorÃ‚Â ) AND (1980:2020 [pdat]) | Lorbrena | Lorlatinib | 2018 |

© 2023 Zhou EW et al. *JAMA Health Forum*.

| | | | | | | |
|---|---|---|---|---|---|---|
| drug286 | "lofexidine"[Supplementary Concept] OR "lofexidine"[All Fields] OR "lucemyra"[All Fields] OR "lofexidine"[Supplementary Concept] OR "lofexidine"[All Fields] OR "lofexidine"[Supplementary Concept] OR "lofexidine"[All Fields] | 191 | (alpha-2-adrenergic receptor) AND (1980:2020 [pdat]) | Lucemyra | Lofexidine | 2018 |
| drug287 | "immunotoxin ha22"[Supplementary Concept] OR "immunotoxin ha22"[All Fields] OR "lumoxiti"[All Fields] OR "moxetumomab pasudotox"[All Fields] OR "immunotoxin ha22"[Supplementary Concept] OR "immunotoxin ha22"[All Fields] OR "moxetumomab pasudotox"[All Fields] OR "immunotoxin ha22"[Supplementary Concept] OR "immunotoxin ha22"[All Fields] OR "moxetumomab pasudotox"[All Fields] | 156 | (CD22) AND (1980:2020 [pdat]) | Lumoxiti | Moxetumomab pasudotox | 2018 |
| drug288 | "binimetinib"[Supplementary Concept] OR "binimetinib"[All Fields] OR "mektovi"[All Fields] OR "binimetinib"[Supplementary Concept] OR "binimetinib"[All Fields] OR "binimetinib"[Supplementary Concept] OR "binimetinib"[All Fields] | 96 | (mitogen-activated protein kinase kinases[MeSH Terms]) AND (1980:2020 [pdat]) | Mektovi | Binimetinib | 2018 |
| drug289 | "prucalopride"[Supplementary Concept] OR "prucalopride"[All Fields] OR "motegrity"[All Fields] OR ("prucalopride"[Supplementary Concept] OR "prucalopride"[All Fields]) OR (("prucalopride"[Supplementary Concept] OR "prucalopride"[All Fields]) AND ("succinates"[MeSH Terms] OR "succinates"[All Fields] OR "succination"[All Fields] OR "succinic acid"[MeSH Terms] OR ("succinic"[All Fields] AND "acid"[All Fields]) OR "succinic acid"[All Fields] OR "succinate"[All Fields] OR "succinic"[All Fields])) | 195 | (5-HT4) AND (1980:2020 [pdat]) | Motegrity | Prucalopride | 2018 |

© 2023 Zhou EW et al. *JAMA Health Forum.*

| | | | | | | |
|---|---|---|---|---|---|---|
| drug29 | "n 3 amino 1 cyclobutylmethyl 2 3 dioxopropyl 3 2 1 1 dimethylethyl amino carbonyl amino 3 3 dimethyl 1 oxobutyl 6 6 dimethyl 3 azabicyclo 3 1 0 hexan 2 carboxamide"[Supplementary Concept] OR "n 3 amino 1 cyclobutylmethyl 2 3 dioxopropyl 3 2 1 1 dimethylethyl amino carbonyl amino 3 3 dimethyl 1 oxobutyl 6 6 dimethyl 3 azabicyclo 3 1 0 hexan 2 carboxamide"[All Fields] OR "boceprevir"[All Fields] OR "victrelis"[All Fields] OR "n 3 amino 1 cyclobutylmethyl 2 3 dioxopropyl 3 2 1 1 dimethylethyl amino carbonyl amino 3 3 dimethyl 1 oxobutyl 6 6 dimethyl 3 azabicyclo 3 1 0 hexan 2 carboxamide"[Supplementary Concept] OR "n 3 amino 1 cyclobutylmethyl 2 3 dioxopropyl 3 2 1 1 dimethylethyl amino carbonyl amino 3 3 dimethyl 1 oxobutyl 6 6 dimethyl 3 azabicyclo 3 1 0 hexan 2 carboxamide"[All Fields] OR "boceprevir"[All Fields] OR "n 3 amino 1 cyclobutylmethyl 2 3 dioxopropyl 3 2 1 1 dimethylethyl amino carbonyl amino 3 3 dimethyl 1 oxobutyl 6 6 dimethyl 3 azabicyclo 3 1 0 hexan 2 carboxamide"[Supplementary Concept] OR "n 3 amino 1 cyclobutylmethyl 2 3 dioxopropyl 3 2 1 1 dimethylethyl amino carbonyl amino 3 3 dimethyl 1 oxobutyl 6 6 dimethyl 3 azabicyclo 3 1 0 hexan 2 carboxamide"[All Fields] OR "boceprevir"[All Fields] | 72 | (HCV NS3) AND (1980:2020 [pdat]) | Victrelis | Boceprevir | 2011 |

© 2023 Zhou EW et al. *JAMA Health Forum.*

| | | | | | | |
|---|---|---|---|---|---|---|
| drug290 | "moxidectin"[Supplementary Concept] OR "moxidectin"[All Fields] OR "moxidectin"[Supplementary Concept] OR "moxidectin"[All Fields] OR "moxidectin"[Supplementary Concept] OR "moxidectin"[All Fields] | 45 | ("chloride channels"[MeSH Terms]) AND (1980:2020 [pdat]) | Moxidectin | Moxidectin | 2018 |
| drug291 | "lusutrombopag"[Supplementary Concept] OR "lusutrombopag"[All Fields] OR "mulpleta"[All Fields] OR "lusutrombopag"[Supplementary Concept] OR "lusutrombopag"[All Fields] OR "lusutrombopag"[Supplementary Concept] OR "lusutrombopag"[All Fields] | 188 | (Thrombopoietin receptor) AND (1980:2020 [pdat]) | Mulpleta | Lusutrombopag | 2018 |
| drug292 | "prt064445"[Supplementary Concept] OR "prt064445"[All Fields] OR "andexanet alfa"[All Fields] OR "andexxa"[All Fields] OR ((("factor xa"[MeSH Terms] OR ("factor"[All Fields] AND "xa"[All Fields]) OR "factor xa"[All Fields] OR ("coagulation"[All Fields] AND "factor"[All Fields] AND "xa"[All Fields]) OR "coagulation factor xa"[All Fields]) AND ("f8 protein human"[Supplementary Concept] OR "f8 protein human"[All Fields] OR | 250 | (30S ribosomal) AND (1980:2020 [pdat]) | Nuzyra | Omadacycline | 2018 |

© 2023 Zhou EW et al. *JAMA Health Forum.*

| | | | | | | |
|---|---|---|---|---|---|---|
| | "recombinate"[All Fields] OR "recombinant"[All Fields] OR "recombinants"[All Fields] OR "recombinated"[All Fields] OR "recombinates"[All Fields] OR "recombination, genetic"[MeSH Terms] OR ("recombination"[All Fields] AND "genetic"[All Fields]) OR "genetic recombination"[All Fields] OR "recombination"[All Fields] OR "recombinations"[All Fields] OR "recombinational"[All Fields] OR "recombinative"[All Fields] OR "recombine"[All Fields] OR "recombined"[All Fields] OR "recombineered"[All Fields] OR "recombineering"[All Fields] OR "recombines"[All Fields] OR "recombining"[All Fields])) AND ("inactivate"[All Fields] OR "inactivated"[All Fields] OR "inactivates"[All Fields] OR "inactivating"[All Fields] OR "inactivation"[All Fields] OR "inactivations"[All Fields] OR "inactivator"[All Fields] OR "inactivators"[All Fields] OR "inactived"[All Fields])) OR ("prt064445"[Supplementary Concept] OR "prt064445"[All Fields] OR "andexanet alfa"[All Fields]) | | | | | |

© 2023 Zhou EW et al. *JAMA Health Forum.*

| | | | | | | |
|---|---|---|---|---|---|---|
| drug293 | "Hyqvia"[All Fields] OR ((("human s"[All Fields] OR "humans"[MeSH Terms] OR "humans"[All Fields] OR "human"[All Fields]) AND ("immunoglobulin g"[MeSH Terms] OR "immunoglobulin g"[All Fields])) AND ("immunoglobulins, intravenous"[MeSH Terms] OR ("immunoglobulins"[All Fields] AND "intravenous"[All Fields]) OR "intravenous immunoglobulins"[All Fields] OR "ivig"[All Fields])) OR (("immunoglobulin s"[All Fields] OR "immunoglobuline"[All Fields] OR "immunoglobulines"[All Fields] OR "immunoglobulins"[MeSH Terms] OR "immunoglobulins"[All Fields] OR "immunoglobulin"[All Fields]) AND "Baxter-2"[All Fields] AND "Enhanze"[All Fields]) | 85 | (janus kinases[MeSH Terms]) AND (1980:2020 [pdat]) | Olumiant | Baricitinib | 2018 |
| drug295 | "patisiran"[Supplementary Concept] OR "patisiran"[All Fields] OR "onpattro"[All Fields] OR "patisiran"[Supplementary Concept] OR "patisiran"[All Fields] OR "patisiran"[Supplementary Concept] OR "patisiran"[All Fields] | 192 | (Transthyretin) AND (1980:2020 [pdat]) | Onpattro | Patisiran | 2018 |

© 2023 Zhou EW et al. *JAMA Health Forum.*

| | | | | | | |
|---|---|---|---|---|---|---|
| drug296 | "elagolix"[Supplementary Concept] OR "elagolix"[All Fields] OR "orilissa"[All Fields] OR "elagolix"[Supplementary Concept] OR "elagolix"[All Fields] OR "elagolix sodium"[All Fields] OR "elagolix"[Supplementary Concept] OR "elagolix"[All Fields] | 193 | (Gonadotropin-releasing hormone receptor) AND (1980:2020 [pdat]) | Orilissa | Elagolix sodium | 2018 |
| drug297 | "cenegermin"[Supplementary Concept] OR "cenegermin"[All Fields] OR "oxervate"[All Fields] OR "cenegermin"[Supplementary Concept] OR "cenegermin"[All Fields] OR "cenegermin"[Supplementary Concept] OR "cenegermin"[All Fields] | 223 | ("nerve growth factor") AND (1980:2020 [pdat]) | Oxervate | Cenegermin | 2018 |
| drug298 | "pegvaliase"[Supplementary Concept] OR "pegvaliase"[All Fields] OR "palynziq"[All Fields] OR "pegvaliase"[Supplementary Concept] OR "pegvaliase"[All Fields] OR "pegvaliase"[Supplementary Concept] OR "pegvaliase"[All Fields] | 182 | (phenylalanine ammonia) AND (1980:2020 [pdat]) | Palynziq | Pegvaliase | 2018 |
| drug299 | "doravirine"[Supplementary Concept] OR "doravirine"[All Fields] OR "pifeltro"[All Fields] OR "doravirine"[Supplementary Concept] OR "doravirine"[All Fields] OR "doravirine"[Supplementary Concept] OR "doravirine"[All Fields] | 66 | (HIV reverse transcriptase) AND (1980:2020 [pdat]) | Pifeltro | Doravirine | 2018 |
| drug3 | "ado trastuzumab emtansine"[MeSH Terms] OR ("ado trastuzumab"[All Fields] AND "emtansine"[All Fields]) OR "ado trastuzumab emtansine"[All Fields] OR "kadcyla"[All Fields] OR ("ado"[All Fields] AND "trastuzumab"[All Fields] AND "emtansine"[All Fields]) OR "ado trastuzumab emtansine"[All Fields] OR "trastuzumab"[MeSH Terms] OR "trastuzumab"[All Fields] OR "trastuzumab s"[All Fields] OR ("ado trastuzumab emtansine"[MeSH Terms] OR ("ado trastuzumab"[All Fields] AND "emtansine"[All Fields]) OR "ado trastuzumab emtansine"[All Fields] OR ("ado"[All Fields] AND "trastuzumab"[All Fields] AND "emtansine"[All Fields]) OR "ado trastuzumab emtansine"[All | 77 | (HER2 OR eErb2 OR p185) AND (1980:2020 [pdat]) | Kadcyla | Ado-trastuzumab emtansine | 2013 |

© 2023 Zhou EW et al. *JAMA Health Forum.*

| | | | | | | |
|---|---|---|---|---|---|---|
| | Fields]) OR ("ado trastuzumab emtansine"[MeSH Terms] OR ("ado trastuzumab"[All Fields] AND "emtansine"[All Fields]) OR "ado trastuzumab emtansine"[All Fields] OR ("trastuzumab"[All Fields] AND "emtansine"[All Fields]) OR "trastuzumab emtansine"[All Fields]) | | | | | |
| drug30 | "bosulif"[All Fields] OR "bosutinib"[Supplementary Concept] OR "bosutinib"[All Fields] OR "bosutinib"[Supplementary Concept] OR "bosutinib"[All Fields] OR "bosutinib"[Supplementary Concept] OR "bosutinib"[All Fields] | 138 | (src kinase) AND (1980:2020 [pdat]) | Bosulif | Bosutinib | 2012 |
| drug300 | "diroximel fumarate"[All Fields] OR "vumerity"[All Fields] OR ("diroximel"[All Fields] AND ("fumarates"[MeSH Terms] OR "fumarates"[All Fields] OR "fumarate"[All Fields] OR "fumaric"[All Fields])) OR ("diroximel"[All Fields] AND ("fumarates"[MeSH Terms] OR "fumarates"[All Fields] OR "fumarate"[All Fields] OR "fumaric"[All Fields])) | 175 | (CCR4) AND (1980:2020 [pdat]) | Poteligeo | Mogamulizumab | 2018 |
| drug301 | "t 91825"[Supplementary Concept] OR "t 91825"[All Fields] OR "ceftaroline"[All Fields] OR "ceftaroline fosamil"[Supplementary Concept] OR "ceftaroline fosamil"[All Fields] OR "teflaro"[All Fields] OR ("ceftaroline fosamil"[Supplementary Concept] OR "ceftaroline fosamil"[All Fields]) OR (("t 91825"[Supplementary Concept] OR "t | 251 | ("adenosine deaminase") AND (1980:2020 [pdat]) | Revcovi | Elapegademase | 2018 |

© 2023 Zhou EW et al. *JAMA Health Forum.*

| | | | | | | |
|---|---|---|---|---|---|---|
| | 91825"[All Fields] OR "ceftaroline"[All Fields]) AND ("acetalization"[All Fields] OR "acetalizations"[All Fields] OR "acetalized"[All Fields] OR "acetals"[MeSH Terms] OR "acetals"[All Fields] OR "acetal"[All Fields] OR "acetates"[MeSH Terms] OR "acetates"[All Fields] OR "acetate"[All Fields] OR "acetic"[All Fields])) | | | | | |
| drug302 | "sarecycline"[Supplementary Concept] OR "sarecycline"[All Fields] OR "seysara"[All Fields] OR "sarecycline"[Supplementary Concept] OR "sarecycline"[All Fields] OR "sarecycline"[Supplementary Concept] OR "sarecycline"[All Fields] | 0 | | Seysara | Sarecycline | 2018 |
| drug303 | "tezacaftor ivacaftor drug combination"[Supplementary Concept] OR "tezacaftor ivacaftor drug combination"[All Fields] OR "symdeko"[All Fields] OR ("tezacaftor ivacaftor drug combination"[Supplementary Concept] OR "tezacaftor ivacaftor drug combination"[All Fields] OR "tezacaftor and ivacaftor"[All Fields]) OR (("ivacaftor"[Supplementary Concept] OR "ivacaftor"[All Fields]) AND ("tezacaftor"[Supplementary Concept] OR "tezacaftor"[All Fields]) AND ("vertex"[All Fields] OR "vertexes"[All Fields])) | 44 | (CFTR) AND (1980:2020 [pdat]) | Symdeko | Tezacaftor and ivacaftor | 2018 |

© 2023 Zhou EW et al. *JAMA Health Forum.*

| | | | | | | |
|---|---|---|---|---|---|---|
| drug304 | "lanadelumab"[Supplementary Concept] OR "lanadelumab"[All Fields] OR "takhzyro"[All Fields] OR "lanadelumab"[Supplementary Concept] OR "lanadelumab"[All Fields] OR "lanadelumab"[Supplementary Concept] OR "lanadelumab"[All Fields] | 176 | (plasma kallikrein) AND (1980:2020 [pdat]) | Takhzyro | Lanadelumab | 2018 |
| drug305 | "talazoparib"[Supplementary Concept] OR "talazoparib"[All Fields] OR "talzenna"[All Fields] OR "talazoparib"[Supplementary Concept] OR "talazoparib"[All Fields] OR "talazoparib"[Supplementary Concept] OR "talazoparib"[All Fields] | 105 | (PARP) AND (1980:2020 [pdat]) | Talzenna | Talazoparib | 2018 |
| drug306 | "fostamatinib"[Supplementary Concept] OR "fostamatinib"[All Fields] OR "tavalisse"[All Fields] OR ("fostamatinib"[Supplementary Concept] OR "fostamatinib"[All Fields]) OR (("fostamatinib"[Supplementary Concept] OR "fostamatinib"[All Fields]) AND "disodium"[All Fields]) | 189 | (spleen tyrosine kinase) AND (1980:2020 [pdat]) | Tavalisse | Fostamatinib | 2018 |
| drug307 | "inotersen"[Supplementary Concept] OR "inotersen"[All Fields] OR "inotersen"[All Fields] OR "tegsedi"[All Fields] OR "inotersen"[Supplementary Concept] OR "inotersen"[All Fields] OR "inotersen"[All Fields] OR "inotersen"[Supplementary Concept] OR "inotersen"[All Fields] OR "inotersen"[All Fields] | 192 | (Transthyretin) AND (1980:2020 [pdat]) | Tegsedi | Inotersen | 2018 |
| drug308 | "ivosidenib"[Supplementary Concept] OR "ivosidenib"[All Fields] OR "tibsovo"[All Fields] OR "ivosidenib"[Supplementary Concept] OR "ivosidenib"[All Fields] OR "ivosidenib"[Supplementary Concept] OR "ivosidenib"[All Fields] | 163 | (isocitrate dehydrogenase) AND (1980:2020 [pdat]) | Tibsovo | Ivosidenib | 2018 |

© 2023 Zhou EW et al. *JAMA Health Forum.*

| | | | | | | |
|---|---|---|---|---|---|---|
| drug309 | "tecovirimat"[Supplementary Concept] OR "tecovirimat"[All Fields] OR "tecovirimat"[All Fields] OR "tpoxx"[All Fields] OR "tecovirimat"[Supplementary Concept] OR "tecovirimat"[All Fields] OR "tecovirimat"[All Fields] OR "tecovirimat"[Supplementary Concept] OR "tecovirimat"[All Fields] OR "tecovirimat"[All Fields] | 184 | ("orthopoxvirus envelope") AND (1980:2020 [pdat]) | TPOXX | Tecovirimat | 2018 |
| drug31 | "brentuximab vedotin"[MeSH Terms] OR ("brentuximab"[All Fields] AND "vedotin"[All Fields]) OR "brentuximab vedotin"[All Fields] OR "adcetris"[All Fields] OR ("brentuximab vedotin"[MeSH Terms] OR ("brentuximab"[All Fields] AND "vedotin"[All Fields]) OR "brentuximab vedotin"[All Fields]) OR ("brentuximab vedotin"[MeSH Terms] OR ("brentuximab"[All Fields] AND "vedotin"[All Fields]) OR "brentuximab vedotin"[All Fields]) | 39 | (CD30) AND (1980:2020 [pdat]) | Adcetris | Brentuximab vedotin | 2011 |
| drug310 | "ibalizumab"[Supplementary Concept] OR "ibalizumab"[All Fields] OR "trogarzo"[All Fields] OR "ibalizumab"[Supplementary Concept] OR "ibalizumab"[All Fields] OR "ibalizumab"[Supplementary Concept] OR "ibalizumab"[All Fields] | 172 | (CD4) AND (1980:2020 [pdat]) | Trogarzo | Ibalizumab | 2018 |
| drug311 | "ravulizumab"[Supplementary Concept] OR "ravulizumab"[All Fields] OR "ultomiris"[All Fields] OR "ravulizumab"[Supplementary Concept] OR "ravulizumab"[All Fields] OR "ravulizumab"[Supplementary Concept] OR "ravulizumab"[All Fields] | 180 | ("complement C5 protein") AND (1980:2020 [pdat]) | Ultomiris | Ravulizumab | 2018 |
| drug312 | "larotrectinib"[Supplementary Concept] OR "larotrectinib"[All Fields] OR "vitrakvi"[All Fields] OR "larotrectinib"[Supplementary Concept] OR "larotrectinib"[All Fields] OR "larotrectinib"[Supplementary Concept] OR "larotrectinib"[All Fields] | 186 | (tropomyosin receptor kinases) AND (1980:2020 [pdat]) | Vitrakvi | Larotrectinib | 2018 |

© 2023 Zhou EW et al. *JAMA Health Forum.*

| | | | | | | |
|---|---|---|---|---|---|---|
| drug313 | "dacomitinib"[Supplementary Concept] OR "dacomitinib"[All Fields] OR "vizimpro"[All Fields] OR "dacomitinib"[Supplementary Concept] OR "dacomitinib"[All Fields] OR "dacomitinib"[Supplementary Concept] OR "dacomitinib"[All Fields] | 187 | (Epidermal growth factor receptor) AND (1980:2020 [pdat]) | Vizimpro | Dacomitinib | 2018 |
| drug314 | "ceftolozane"[Supplementary Concept] OR "ceftolozane"[All Fields] OR "ceftolozane tazobactam drug combination"[Supplementary Concept] OR "ceftolozane tazobactam drug combination"[All Fields] OR "zerbaxa"[All Fields] OR "tazobactam"[MeSH Terms] OR "tazobactam"[All Fields] OR (("ceftolozane"[Supplementary Concept] OR "ceftolozane"[All Fields]) AND ("tazobactam"[MeSH Terms] OR "tazobactam"[All Fields])) OR (("ceftolozane"[Supplementary Concept] OR "ceftolozane"[All Fields] OR "ceftolozane sulfate"[All Fields]) AND ("tazobactam"[MeSH Terms] OR "tazobactam"[All Fields])) | 0 | | Xerava | Eravacycline | 2018 |
| drug315 | "baloxavir"[Supplementary Concept] OR "baloxavir"[All Fields] OR "baloxavir marboxil"[All Fields] OR "xofluza"[All Fields] OR "baloxavir"[Supplementary Concept] OR "baloxavir"[All Fields] OR "baloxavir marboxil"[All Fields] OR "baloxavir"[Supplementary Concept] OR "baloxavir"[All Fields] OR "baloxavir marboxil"[All Fields] | 224 | (Polymerase Acidic Endonuclease) AND (1980:2020 [pdat]) | Xofluza | Baloxavir marboxil | 2018 |
| drug316 | "gilteritinib"[Supplementary Concept] OR "gilteritinib"[All Fields] OR "xospata"[All Fields] OR "gilteritinib"[Supplementary Concept] OR "gilteritinib"[All Fields] OR "gilteritinib"[Supplementary Concept] OR "gilteritinib"[All Fields] | 123 | (receptor tyrosine kinase) AND (1980:2020 [pdat]) | Xospata | Gilteritinib | 2018 |

© 2023 Zhou EW et al. *JAMA Health Forum.*

| | | | | | | |
|---|---|---|---|---|---|---|
| drug317 | "revefenacin"[Supplementary Concept] OR "revefenacin"[All Fields] OR "yupelri"[All Fields] OR "revefenacin"[Supplementary Concept] OR "revefenacin"[All Fields] OR "revefenacin"[Supplementary Concept] OR "revefenacin"[All Fields] | 226 | (muscarinic receptor) AND (1980:2020 [pdat]) | Yupelri | Revefenacin | 2018 |
| drug318 | "efinaconazole"[Supplementary Concept] OR "efinaconazole"[All Fields] OR "jublia"[All Fields] OR "efinaconazole"[Supplementary Concept] OR "efinaconazole"[All Fields] OR "efinaconazole"[Supplementary Concept] OR "efinaconazole"[All Fields] | 250 | (30S ribosomal) AND (1980:2020 [pdat]) | Zemdri | Plazomicin | 2018 |
| drug319 | "finafloxacin"[Supplementary Concept] OR "finafloxacin"[All Fields] OR "xtoro"[All Fields] OR "finafloxacin"[Supplementary Concept] OR "finafloxacin"[All Fields] OR "finafloxacin"[Supplementary Concept] OR "finafloxacin"[All Fields] | 0 | | Egaten | Triclabendazole | 2019 |
| drug32 | "brexpiprazole"[Supplementary Concept] OR "brexpiprazole"[All Fields] OR "rexulti"[All Fields] OR "brexpiprazole"[Supplementary Concept] OR "brexpiprazole"[All Fields] OR "brexpiprazole"[Supplementary Concept] OR "brexpiprazole"[All Fields] | 3 | ((dopamine receptor) OR serotonin receptor) AND (1980:2020 [pdat]) | Rexulti | Brexpiprazole | 2015 |
| drug320 | "brexanolone"[Supplementary Concept] OR "brexanolone"[All Fields] OR "zulresso"[All Fields] OR "brexanolone"[Supplementary Concept] OR "brexanolone"[All Fields] OR "brexanolone"[Supplementary Concept] OR "brexanolone"[All Fields] | 190 | (GABA-A receptor) AND (1980:2020 [pdat]) | Zulresso | Brexanolone | 2019 |
| drug321 | "solriamfetol"[Supplementary Concept] OR "solriamfetol"[All Fields] OR "sunosi"[All Fields] OR "solriamfetol"[Supplementary Concept] OR "solriamfetol"[All Fields] OR "solriamfetol"[Supplementary Concept] OR "solriamfetol"[All Fields] | 234 | ((noradrenaline reuptake) OR (dopamine reuptake)) AND (1980:2020 [pdat]) | Sunosi | Solriamfetol | 2019 |

© 2023 Zhou EW et al. *JAMA Health Forum.*

| | | | | | | |
|---|---|---|---|---|---|---|
| drug322 | "siponimod"[Supplementary Concept] OR "siponimod"[All Fields] OR "mayzent"[All Fields] OR "siponimod"[Supplementary Concept] OR "siponimod"[All Fields] OR "siponimod"[Supplementary Concept] OR "siponimod"[All Fields] | 207 | (sphingosine 1-phosphate) AND (1980:2020 [pdat]) | Mayzent | Siponimod | 2019 |
| drug323 | "erdafitinib"[Supplementary Concept] OR "erdafitinib"[All Fields] OR "balversa"[All Fields] OR "erdafitinib"[Supplementary Concept] OR "erdafitinib"[All Fields] OR "erdafitinib"[Supplementary Concept] OR "erdafitinib"[All Fields] | 204 | (fibroblast growth factor receptor) AND (1980:2020 [pdat]) | Balversa | Erdafitinib | 2019 |
| drug324 | "tafamidis"[Supplementary Concept] OR "tafamidis"[All Fields] OR "vyndaqel"[All Fields] OR "tafamidis"[Supplementary Concept] OR "tafamidis"[All Fields] OR "tafamidis meglumine"[All Fields] OR "tafamidis"[Supplementary Concept] OR "tafamidis"[All Fields] | 192 | (Transthyretin) AND (1980:2020 [pdat]) | Vyndaqel | Tafamidis meglumine | 2019 |
| drug325 | "alpelisib"[Supplementary Concept] OR "alpelisib"[All Fields] OR "alpelisib"[All Fields] OR "piqray"[All Fields] OR "alpelisib"[Supplementary Concept] OR "alpelisib"[All Fields] OR "alpelisib"[All Fields] OR "alpelisib"[Supplementary Concept] OR "alpelisib"[All Fields] OR "alpelisib"[All Fields] | 109 | (Phosphoinositide 3-kinase OR PI-3 kinase) AND (1980:2020 [pdat]) | Piqray | Alpelisib | 2019 |
| drug326 | "bremelanotide"[Supplementary Concept] OR "bremelanotide"[All Fields] OR "vyleesi"[All Fields] OR ("bremelanotide"[Supplementary Concept] OR "bremelanotide"[All Fields]) OR (("bremelanotide"[Supplementary Concept] OR "bremelanotide"[All Fields]) AND ("acetalization"[All Fields] OR "acetalizations"[All Fields] OR "acetalized"[All Fields] OR "acetals"[MeSH Terms] OR "acetals"[All Fields] OR "acetal"[All Fields] OR "acetates"[MeSH Terms] OR "acetates"[All Fields] OR "acetate"[All Fields] OR "acetic"[All Fields])) | 208 | (melanocortin receptor) AND (1980:2020 [pdat]) | Vyleesi | Bremelanotide | 2019 |

© 2023 Zhou EW et al. *JAMA Health Forum.*

| | | | | | | |
|---|---|---|---|---|---|---|
| drug327 | "selinexor"[Supplementary Concept] OR "selinexor"[All Fields] OR "xpovio"[All Fields] OR "selinexor"[Supplementary Concept] OR "selinexor"[All Fields] OR "selinexor"[Supplementary Concept] OR "selinexor"[All Fields] | 205 | (exportin) AND (1980:2020 [pdat]) | Xpovio | Selinexor | 2019 |
| drug329 | "darolutamide"[Supplementary Concept] OR "darolutamide"[All Fields] OR "nubeqa"[All Fields] OR "darolutamide"[Supplementary Concept] OR "darolutamide"[All Fields] OR "darolutamide"[Supplementary Concept] OR "darolutamide"[All Fields] | 17 | (androgen receptor) AND (1980:2020 [pdat]) | Nubeqa | Darolutamide | 2019 |
| drug33 | "brivaracetam"[Supplementary Concept] OR "brivaracetam"[All Fields] OR "briviact"[All Fields] OR "brivaracetam"[Supplementary Concept] OR "brivaracetam"[All Fields] OR "brivaracetam"[Supplementary Concept] OR "brivaracetam"[All Fields] | 0 | | Briviact | Brivaracetam | 2016 |
| drug330 | "pexidartinib"[Supplementary Concept] OR "pexidartinib"[All Fields] OR "turalio"[All Fields] OR "pexidartinib"[Supplementary Concept] OR "pexidartinib"[All Fields] OR "pexidartinib"[Supplementary Concept] OR "pexidartinib"[All Fields] | 232 | (cd117 OR cd115) AND (1980:2020 [pdat]) | Turalio | Pexidartinib hydrochloride | 2019 |
| drug331 | "pitolisant"[Supplementary Concept] OR "pitolisant"[All Fields] OR "wakix"[All Fields] OR (("pitolisant"[Supplementary Concept] OR "pitolisant"[All Fields]) AND ("hydrochlorid"[All Fields] OR "hydrochloride"[All Fields] OR "hydrochlorides"[All Fields])) OR ("pitolisant"[Supplementary Concept] OR "pitolisant"[All Fields]) | 209 | (histamine H3 receptor) AND (1980:2020 [pdat]) | Wakix | Pitolisant hydrochloride | 2019 |

© 2023 Zhou EW et al. *JAMA Health Forum.*

| | | | | | | |
|---|---|---|---|---|---|---|
| drug332 | "pretomanid"[Supplementary Concept] OR "pretomanid"[All Fields] OR "pretomanid"[Supplementary Concept] OR "pretomanid"[All Fields] OR "pretomanid"[Supplementary Concept] OR "pretomanid"[All Fields] | 0 | | Pretomanid | Pretomanid | 2019 |
| drug333 | "entrectinib"[Supplementary Concept] OR "entrectinib"[All Fields] OR "rozlytrek"[All Fields] OR "entrectinib"[Supplementary Concept] OR "entrectinib"[All Fields] OR "entrectinib"[Supplementary Concept] OR "entrectinib"[All Fields] | 123 | (receptor tyrosine kinase) AND (1980:2020 [pdat]) | Rozlytrek | Entrectinib | 2019 |
| drug334 | "upadacitinib"[Supplementary Concept] OR "upadacitinib"[All Fields] OR "rinvoq"[All Fields] OR "upadacitinib"[Supplementary Concept] OR "upadacitinib"[All Fields] OR "upadacitinib"[Supplementary Concept] OR "upadacitinib"[All Fields] | 85 | (janus kinases[MeSH Terms]) AND (1980:2020 [pdat]) | Rinvoq | Upadacitinib | 2019 |
| drug335 | "fedratinib"[Supplementary Concept] OR "fedratinib"[All Fields] OR "fedratinib"[All Fields] OR "inrebic"[All Fields] OR "fedratinib"[Supplementary Concept] OR "fedratinib"[All Fields] OR "fedratinib hydrochloride"[All Fields] OR "fedratinib"[Supplementary Concept] OR "fedratinib"[All Fields] OR "fedratinib"[All Fields] | 123 | (receptor tyrosine kinase) AND (1980:2020 [pdat]) | Inrebic | Fedratinib hydrochloride | 2019 |
| drug336 | "gaa protein human"[Supplementary Concept] OR "gaa protein human"[All Fields] OR "alglucosidase alfa"[All Fields] OR "lumizyme"[All Fields] OR "gaa protein human"[Supplementary Concept] OR "gaa protein human"[All Fields] OR "alglucosidase alfa"[All Fields] OR "gaa protein human"[Supplementary Concept] OR "gaa protein human"[All Fields] OR "alglucosidase alfa"[All Fields] | 249 | (50s ribosomal) AND (1980:2020 [pdat]) | Xenleta | Lefamulin | 2019 |

© 2023 Zhou EW et al. *JAMA Health Forum.*

| | | | | | | |
|---|---|---|---|---|---|---|
| drug337 | "istradefylline"[Supplementary Concept] OR "istradefylline"[All Fields] OR "nourianz"[All Fields] OR "istradefylline"[Supplementary Concept] OR "istradefylline"[All Fields] OR "istradefylline"[Supplementary Concept] OR "istradefylline"[All Fields] | 211 | ("adenosine receptor") AND (1980:2020 [pdat]) | Nourianz | Istradefylline | 2019 |
| drug338 | "ibsrela"[All Fields] OR "tenapanor"[Supplementary Concept] OR "tenapanor"[All Fields] OR ("tenapanor"[Supplementary Concept] OR "tenapanor"[All Fields]) OR (("tenapanor"[Supplementary Concept] OR "tenapanor"[All Fields]) AND ("hydrochlorid"[All Fields] OR "hydrochloride"[All Fields] OR "hydrochlorides"[All Fields])) | 210 | (sodium hydrogen exchanger) AND (1980:2020 [pdat]) | Ibsrela | Tenapanor | 2019 |
| drug339 | "aklief"[All Fields] OR "trifarotene"[Supplementary Concept] OR "trifarotene"[All Fields] OR "trifarotene"[Supplementary Concept] OR "trifarotene"[All Fields] OR "trifarotene"[Supplementary Concept] OR "trifarotene"[All Fields] | 212 | ("retinoic acid receptor") AND (1980:2020 [pdat]) | Aklief | Trifarotene | 2019 |
| drug34 | "cabazitaxel"[Supplementary Concept] OR "cabazitaxel"[All Fields] OR "jevtana"[All Fields] OR "cabazitaxel"[Supplementary Concept] OR "cabazitaxel"[All Fields] OR "cabazitaxel"[Supplementary Concept] OR "cabazitaxel"[All Fields] | 94 | (microtubule assembly) AND (1980:2020 [pdat]) | Jevtana | Cabazitaxel | 2010 |
| drug340 | "afamelanotide"[Supplementary Concept] OR "afamelanotide"[All Fields] OR "scenesse"[All Fields] OR "afamelanotide"[Supplementary Concept] OR "afamelanotide"[All Fields] OR "afamelanotide"[Supplementary Concept] OR "afamelanotide"[All Fields] | 208 | (melanocortin receptor) AND (1980:2020 [pdat]) | Scenesse | Afamelanotide | 2019 |

© 2023 Zhou EW et al. *JAMA Health Forum.*

| | | | | | | |
|---|---|---|---|---|---|---|
| drug341 | "lasmiditan"[Supplementary Concept] OR "lasmiditan"[All Fields] OR "reyvow"[All Fields] OR (("lasmiditan"[Supplementary Concept] OR "lasmiditan"[All Fields]) AND ("succinates"[MeSH Terms] OR "succinates"[All Fields] OR "succination"[All Fields] OR "succinic acid"[MeSH Terms] OR ("succinic"[All Fields] AND "acid"[All Fields]) OR "succinic acid"[All Fields] OR "succinate"[All Fields] OR "succinic"[All Fields])) OR ("lasmiditan"[Supplementary Concept] OR "lasmiditan"[All Fields]) | 233 | (5-HT1 receptor) AND (1980:2020 [pdat]) | Reyvow | Lasmiditan succinate | 2019 |
| drug342 | "elexcaftor ivacaftor tezacaftor drug combination"[Supplementary Concept] OR "elexcaftor ivacaftor tezacaftor drug combination"[All Fields] OR "trikafta"[All Fields] OR (("elexacaftor"[Supplementary Concept] OR "elexacaftor"[All Fields]) AND ("ivacaftor"[Supplementary Concept] OR "ivacaftor"[All Fields]) AND ("tezacaftor"[Supplementary Concept] OR "tezacaftor"[All Fields])) OR (("ivacaftor"[Supplementary Concept] OR "ivacaftor"[All Fields]) AND ("elexacaftor"[Supplementary Concept] OR "elexacaftor"[All Fields]) AND ("vertex"[All Fields] OR "vertexes"[All Fields])) | 44 | (CFTR) AND (1980:2020 [pdat]) | Trikafta | Elexacaftor, ivacaftor, tezacaftor | 2019 |
| drug343 | "botulinum toxins, type a"[MeSH Terms] OR "type a botulinum toxins"[All Fields] OR "botulinum toxin type a"[All Fields] OR "incobotulinumtoxin a"[All Fields] OR "incobotulinumtoxina"[Supplementary Concept] OR "incobotulinumtoxina"[All Fields] OR "xeomin"[All Fields] OR "incobotulinumtoxina"[Supplementary Concept] OR "incobotulinumtoxina"[All Fields] OR "incobotulinumtoxina"[All Fields] OR "incobotulinumtoxina"[Supplementary Concept] OR "incobotulinumtoxina"[All Fields] OR "incobotulinumtoxina"[All Fields] | 238 | (penicillin-binding proteins) AND (1980:2020 [pdat]) | Fetroja | Cefiderocol sulfate tosylate | 2019 |

© 2023 Zhou EW et al. *JAMA Health Forum.*

| | | | | | | |
|---|---|---|---|---|---|---|
| drug344 | "zanubrutinib"[Supplementary Concept] OR "zanubrutinib"[All Fields] OR "brukinsa"[All Fields] OR "zanubrutinib"[Supplementary Concept] OR "zanubrutinib"[All Fields] OR "zanubrutinib"[Supplementary Concept] OR "zanubrutinib"[All Fields] | 33 | (bruton's tyrosine kinase) AND (1980:2020 [pdat]) | Brukinsa | Zanubrutinib | 2019 |
| drug345 | "givosiran"[Supplementary Concept] OR "givosiran"[All Fields] OR "givlaari"[All Fields] OR (("givosiran"[Supplementary Concept] OR "givosiran"[All Fields]) AND ("sodium, dietary"[MeSH Terms] OR ("sodium"[All Fields] AND "dietary"[All Fields]) OR "dietary sodium"[All Fields] OR "sodium"[All Fields] OR "sodium"[MeSH Terms] OR "sodiums"[All Fields])) OR ("givosiran"[Supplementary Concept] OR "givosiran"[All Fields]) | 213 | (aminolevulinate synthase) AND (1980:2020 [pdat]) | Givlaari | Givosiran sodium | 2019 |
| drug346 | "cenobamate"[Supplementary Concept] OR "cenobamate"[All Fields] OR "cenobamate"[All Fields] OR "xcopri"[All Fields] OR "cenobamate"[Supplementary Concept] OR "cenobamate"[All Fields] OR "cenobamate"[All Fields] OR "cenobamate"[Supplementary Concept] OR "cenobamate"[All Fields] OR "cenobamate"[All Fields] | 152 | (voltage gated sodium channel) AND (1980:2020 [pdat]) | Xcopri | Cenobamate | 2019 |
| drug347 | "voxelotor"[Supplementary Concept] OR "voxelotor"[All Fields] OR "oxbryta"[All Fields] OR "voxelotor"[Supplementary Concept] OR "voxelotor"[All Fields] OR "voxelotor"[Supplementary Concept] OR "voxelotor"[All Fields] | 206 | (hemoglobin S) AND (1980:2020 [pdat]) | Oxbryta | Voxelotor | 2019 |
| drug348 | "isavuconazole"[Supplementary Concept] OR "isavuconazole"[All Fields] OR "cresemba"[All Fields] OR "Isavuconazonium"[All Fields] OR "Isavuconazonium"[All Fields] OR ("Isavuconazonium"[All Fields] AND | 246 | (beta lactamases) AND (1980:2020 [pdat]) | Recarbrio | Cilastatin sodium, imipenem, relebactam | 2019 |

© 2023 Zhou EW et al. *JAMA Health Forum.*

| | | | | | | |
|---|---|---|---|---|---|---|
| | ("chlorid"[All Fields] OR "chlorides"[MeSH Terms] OR "chlorides"[All Fields] OR "chloride"[All Fields])) | | | | | |
| drug349 | "caplyta"[All Fields] OR "lumateperone"[Supplementary Concept] OR "lumateperone"[All Fields] OR "lumateperone"[Supplementary Concept] OR "lumateperone"[All Fields] OR "lumateperone"[Supplementary Concept] OR "lumateperone"[All Fields] OR "lumateperone tosylate"[All Fields] | 3 | ((dopamine receptor) OR serotonin receptor) AND (1980:2020 [pdat]) | Caplyta | Lumateperone | 2019 |
| drug35 | "cabozantinib"[Supplementary Concept] OR "cabozantinib"[All Fields] OR "cometriq"[All Fields] OR "cabozantinib"[Supplementary Concept] OR "cabozantinib"[All Fields] OR "cabozantinib"[Supplementary Concept] OR "cabozantinib"[All Fields] | 34 | (c-Met or hepatocyte growth factor) AND (1980:2020 [pdat]) | Cometriq | Cabozantinib | 2012 |
| drug350 | "lemborexant"[Supplementary Concept] OR "lemborexant"[All Fields] OR "dayvigo"[All Fields] OR "lemborexant"[Supplementary Concept] OR "lemborexant"[All Fields] OR "lemborexant"[Supplementary Concept] OR "lemborexant"[All Fields] | 101 | (orexin receptor OR hypocretin receptor) AND (1980:2020 [pdat]) | Dayvigo | Lemborexant | 2019 |
| drug351 | "ubrogepant"[Supplementary Concept] OR "ubrogepant"[All Fields] OR "ubrelvy"[All Fields] OR "ubrogepant"[Supplementary Concept] OR "ubrogepant"[All Fields] OR "ubrogepant"[Supplementary Concept] OR "ubrogepant"[All Fields] | 177 | (calcitonin gene-related peptide receptor) AND (1980:2020 [pdat]) | Ubrelvy | Ubrogepant | 2019 |
| drug352 | "caplacizumab"[Supplementary Concept] OR "caplacizumab"[All Fields] OR "cablivi"[All Fields] OR "caplacizumab"[Supplementary Concept] OR "caplacizumab"[All Fields] OR "caplacizumab yhdp"[All Fields] OR "caplacizumab"[Supplementary Concept] OR "caplacizumab"[All Fields] | 200 | (von willebrand factor) AND (1980:2020 [pdat]) | Cablivi | caplacizumab-yhdp | 2019 |

© 2023 Zhou EW et al. *JAMA Health Forum.*

| | | | | | | |
|---|---|---|---|---|---|---|
| drug353 | "ivabradine"[MeSH Terms] OR "ivabradine"[All Fields] OR "corlanor"[All Fields] OR "ivabradin"[All Fields] OR "ivabradine s"[All Fields] OR "ivabradine"[MeSH Terms] OR "ivabradine"[All Fields] OR "ivabradin"[All Fields] OR "ivabradine s"[All Fields] OR "ivabradine"[MeSH Terms] OR "ivabradine"[All Fields] OR "ivabradin"[All Fields] OR "ivabradine s"[All Fields] | 202 | (SNAP25 OR SNAP-25) AND (1980:2020 [pdat]) | Jeuveau | Prabotulinumtoxina-XVFS | 2019 |
| drug354 | "romosozumab"[Supplementary Concept] OR "romosozumab"[All Fields] OR "evenity"[All Fields] OR "romosozumab"[Supplementary Concept] OR "romosozumab"[All Fields] OR "romosozumab"[Supplementary Concept] OR "romosozumab"[All Fields] | 196 | (sclerostin) AND (1980:2020 [pdat]) | Evenity | Romosozumab | 2019 |
| drug355 | "risankizumab"[Supplementary Concept] OR "risankizumab"[All Fields] OR "skyrizi"[All Fields] OR "risankizumab"[Supplementary Concept] OR "risankizumab"[All Fields] OR "risankizumab rzaa"[All Fields] OR "risankizumab"[Supplementary Concept] OR "risankizumab"[All Fields] | 158 | (interleukin 23) AND (1980:2020 [pdat]) | Skyrizi | risankizumab-rzaa | 2019 |
| drug356 | "polatuzumab vedotin"[Supplementary Concept] OR "polatuzumab vedotin"[All Fields] OR "polivy"[All Fields] OR "polatuzumab vedotin"[Supplementary Concept] OR "polatuzumab vedotin"[All Fields] OR "polatuzumab vedotin piiq"[All Fields] OR "polatuzumab vedotin"[Supplementary Concept] OR "polatuzumab vedotin"[All Fields] | 197 | (CD79b) AND (1980:2020 [pdat]) | Polivy | Polatuzumab vedotin-PIIQ | 2019 |
| drug357 | "beovu"[All Fields] OR "brolucizumab"[Supplementary Concept] OR "brolucizumab"[All Fields] OR "brolucizumab"[Supplementary Concept] OR "brolucizumab"[All Fields] | 203 | (vascular endothelial growth factor A) AND (1980:2020 [pdat]) | Beovu | Brolucizumab-DBII | 2019 |
| drug358 | "luspatercept"[Supplementary Concept] OR "luspatercept"[All Fields] OR "reblozyl"[All Fields] OR "luspatercept"[Supplementary | 198 | (tgf beta) AND (1980:2020 [pdat]) | Reblozyl | Luspatercept-AAMT | 2019 |

© 2023 Zhou EW et al. *JAMA Health Forum.*

| | | | | | | |
|---|---|---|---|---|---|---|
| | Concept] OR "luspatercept"[All Fields] OR "luspatercept aamt"[All Fields] OR "luspatercept"[Supplementary Concept] OR "luspatercept"[All Fields] | | | | | |
| drug359 | "crizanlizumab"[Supplementary Concept] OR "crizanlizumab"[All Fields] OR "adakveo"[All Fields] OR "Crizanlizumab-TMCA"[All Fields] OR "crizanlizumab"[Supplementary Concept] OR "crizanlizumab"[All Fields] | 201 | (p-selectin) AND (1980:2020 [pdat]) | Adakveo | Crizanlizumab-TMCA | 2019 |
| drug36 | "canagliflozin"[MeSH Terms] OR "canagliflozin"[All Fields] OR "invokana"[All Fields] OR "canagliflozin"[MeSH Terms] OR "canagliflozin"[All Fields] OR "canagliflozin"[MeSH Terms] OR "canagliflozin"[All Fields] | 135 | (sodium glucose transporter) AND (1980:2020 [pdat]) | Invokana | Canagliflozin | 2013 |
| drug361 | "Enfortumab"[All Fields] OR "padcev"[All Fields] OR ("Enfortumab"[All Fields] AND "vedotin-EJFV"[All Fields]) OR ("enfortumab vedotin"[Supplementary Concept] OR "enfortumab vedotin"[All Fields]) | 199 | (Nectin) AND (1980:2020 [pdat]) | Padcev | Enfortumab vedotin-EJFV | 2019 |
| drug362 | "enhertu"[All Fields] OR "trastuzumab"[MeSH Terms] OR "trastuzumab"[All Fields] OR "trastuzumab s"[All Fields] OR ("Fam-Trastuzumab"[All Fields] AND "Deruxtecan-NXKI"[All Fields]) OR ("trastuzumab deruxtecan"[Supplementary Concept] OR "trastuzumab deruxtecan"[All Fields]) | 229 | (HER2 OR eErb2 OR p185 or topoisomerase i) AND (1980:2020 [pdat]) | Enhertu | Fam-Trastuzumab Deruxtecan-NXKI | 2019 |
| drug363 | "sipuleucel t"[Supplementary Concept] OR "sipuleucel t"[All Fields] OR "provenge"[All Fields] OR "sipuleucel t"[All Fields] OR "sipuleucel t"[Supplementary Concept] OR "sipuleucel t"[All Fields] OR "sipuleucel t"[All Fields] OR "sipuleucel t"[Supplementary Concept] OR "sipuleucel t"[All Fields] OR "sipuleucel t"[All Fields] | 214 | (Prostatic acid phosphatase) AND (1980:2020 [pdat]) | Provenge | Sipuleucel-T | 2010 |

© 2023 Zhou EW et al. *JAMA Health Forum.*

| | | | | | | |
|---|---|---|---|---|---|---|
| drug364 | "alpha 1 antitrypsin"[MeSH Terms] OR ("alpha"[All Fields] AND "1 antitrypsin"[All Fields]) OR "alpha 1 antitrypsin"[All Fields] OR "alpha 1 antitrypsin"[All Fields] OR "glassia"[All Fields] OR ("alpha 1 antitrypsin"[MeSH Terms] OR ("alpha"[All Fields] AND "1 antitrypsin"[All Fields]) OR "alpha 1 antitrypsin"[All Fields] OR "alpha 1 proteinase inhibitor"[All Fields]) OR (("alpha 1 antitrypsin"[MeSH Terms] OR ("alpha"[All Fields] AND "1 antitrypsin"[All Fields]) OR "alpha 1 antitrypsin"[All Fields] OR "alpha 1 antitrypsin"[All Fields]) AND "iv"[All Fields] AND ("kamada"[All Fields] OR "kamada s"[All Fields])) | 215 | (Alpha-1-antitrypsin) AND (1980:2020 [pdat]) | Glassia | Alpha-1-proteinase inhibitor | 2010 |
| drug365 | "factor viii"[MeSH Terms] OR ("factor"[All Fields] AND "viii"[All Fields]) OR "factor viii"[All Fields] OR "novoeight"[All Fields] OR ("recombinant factor viii n8"[Supplementary Concept] OR "recombinant factor viii n8"[All Fields] OR "turoctocog alfa"[All Fields]) OR ("recombinant factor viii n8"[Supplementary Concept] OR "recombinant factor viii n8"[All Fields] OR "turoctocog alfa"[All Fields]) | 216 | (coagulation "factor VIII") AND (1980:2020 [pdat]) | Novoeight | Turoctocog alfa | 2013 |
| drug366 | "factor ix"[MeSH Terms] OR ("factor"[All Fields] AND "ix"[All Fields]) OR "factor ix"[All Fields] OR "rixubis"[All Fields] OR (("factor ix"[MeSH Terms] OR ("factor"[All Fields] AND "ix"[All Fields]) OR "factor ix"[All Fields] OR ("coagulation"[All Fields] AND "factor"[All Fields] AND "ix"[All Fields]) OR "coagulation factor ix"[All Fields]) AND ("f8 protein human"[Supplementary Concept] OR "f8 protein human"[All Fields] OR "recombinate"[All Fields] OR "recombinant"[All Fields] OR "recombinants"[All Fields] OR "recombinated"[All Fields] OR "recombinates"[All Fields] OR "recombination, genetic"[MeSH Terms] OR ("recombination"[All | 217 | (coagulation factor IX) AND (1980:2020 [pdat]) | Rixubis | Coagulation Factor IX (Recombinant) | 2013 |

© 2023 Zhou EW et al. *JAMA Health Forum.*

| | | | | | | |
|---|---|---|---|---|---|---|
| | Fields] AND "genetic"[All Fields]) OR "genetic recombination"[All Fields] OR "recombination"[All Fields] OR "recombinations"[All Fields] OR "recombinational"[All Fields] OR "recombinative"[All Fields] OR "recombine"[All Fields] OR "recombined"[All Fields] OR "recombineered"[All Fields] OR "recombineering"[All Fields] OR "recombines"[All Fields] OR "recombining"[All Fields])) OR ("nonacog"[All Fields] AND ("gamma rays"[MeSH Terms] OR ("gamma"[All Fields] AND "rays"[All Fields]) OR "gamma rays"[All Fields] OR "gamma"[All Fields] OR "gamma s"[All Fields] OR "gammae"[All Fields] OR "gammas"[All Fields])) | | | | | |
| drug367 | "delafloxacin"[Supplementary Concept] OR "delafloxacin"[All Fields] OR "baxdela"[All Fields] OR "delafloxacin"[Supplementary Concept] OR "delafloxacin"[All Fields] OR "delafloxacin"[Supplementary Concept] OR "delafloxacin"[All Fields] | 218 | (Coagulation Factor XIII) AND (1980:2020 [pdat]) | Tretten | Coagulation Factor XIII A Subunit (Recombinant) | 2013 |

© 2023 Zhou EW et al. *JAMA Health Forum.*

| | | | | | | |
|---|---|---|---|---|---|---|
| drug368 | "factor viii"[MeSH Terms] OR ("factor"[All Fields] AND "viii"[All Fields]) OR "factor viii"[All Fields] OR "obizur"[All Fields] OR ("Susoctocog"[All Fields] AND "alfa"[All Fields]) OR ("Susoctocog"[All Fields] AND "alfa"[All Fields]) | 216 | (coagulation "factor VIII") AND (1980:2020 [pdat]) | Obizur | Susoctocog alfa | 2014 |
| drug369 | "complement c1 inhibitor protein"[MeSH Terms] OR ("complement"[All Fields] AND "c1"[All Fields] AND "inhibitor"[All Fields] AND "protein"[All Fields]) OR "complement c1 inhibitor protein"[All Fields] OR ("c1"[All Fields] AND "esterase"[All Fields] AND "inhibitor"[All Fields]) OR "c1 esterase inhibitor"[All Fields] OR "conestat alfa"[Supplementary Concept] OR "conestat alfa"[All Fields] OR "ruconest"[All Fields] OR ("conestat alfa"[Supplementary Concept] OR "conestat alfa"[All Fields]) OR ("conestat alfa"[Supplementary Concept] OR "conestat alfa"[All Fields]) | 219 | (C1 esterase) AND (1980:2020 [pdat]) | Ruconest | Conestat alfa | 2014 |
| drug37 | "cangrelor"[Supplementary Concept] OR "cangrelor"[All Fields] OR "kengreal"[All Fields] OR "cangrelor"[Supplementary Concept] OR "cangrelor"[All Fields] OR "cangrelor"[Supplementary Concept] OR "cangrelor"[All Fields] OR "cangrelor tetrasodium"[All Fields] | 103 | (p2y receptor) AND (1980:2020 [pdat]) | Kengreal | Cangrelor | 2015 |
| drug370 | "efmoroctocog alfa"[All Fields] OR "factor viii fc fusion protein"[Supplementary Concept] OR "factor viii fc fusion protein"[All Fields] OR "eloctate"[All Fields] OR ("Efmoroctocog"[All Fields] AND "alfa"[All Fields]) OR ("efraloctocog"[All Fields] AND "alfa"[All Fields]) | 216 | (coagulation "factor VIII") AND (1980:2020 [pdat]) | Eloctate | Efmoroctocog alfa | 2014 |

© 2023 Zhou EW et al. *JAMA Health Forum.*

| | | | | | | |
|---|---|---|---|---|---|---|
| drug371 | "factor ix fc fusion protein"[Supplementary Concept] OR "factor ix fc fusion protein"[All Fields] OR "alprolix"[All Fields] OR "factor ix fc fusion protein"[Supplementary Concept] OR "factor ix fc fusion protein"[All Fields] OR "eftrenonacog alfa"[All Fields] OR "factor ix fc fusion protein"[Supplementary Concept] OR "factor ix fc fusion protein"[All Fields] OR "eftrenonacog alfa"[All Fields] | 217 | (coagulation factor IX) AND (1980:2020 [pdat]) | Alprolix | Eftrenonacog alfa | 2014 |
| drug372 | "bax 855"[Supplementary Concept] OR "bax 855"[All Fields] OR "adynovate"[All Fields] OR "factor viii"[MeSH Terms] OR ("factor"[All Fields] AND "viii"[All Fields]) OR "factor viii"[All Fields] OR ((("antihaemophilic factor"[All Fields] OR "factor viii"[MeSH Terms] OR ("factor"[All Fields] AND "viii"[All Fields]) OR "factor viii"[All Fields] OR ("antihemophilic"[All Fields] AND "factor"[All Fields]) OR "antihemophilic factor"[All Fields]) AND ("f8 protein human"[Supplementary Concept] OR "f8 protein human"[All Fields] OR "recombinate"[All Fields] OR "recombinant"[All Fields] OR "recombinants"[All Fields] OR "recombinated"[All Fields] OR "recombinates"[All Fields] OR "recombination, genetic"[MeSH Terms] OR ("recombination"[All Fields] AND "genetic"[All Fields]) OR "genetic recombination"[All Fields] OR "recombination"[All Fields] OR "recombinations"[All Fields] OR "recombinational"[All Fields] OR "recombinative"[All Fields] OR "recombine"[All Fields] OR "recombined"[All Fields] OR "recombineered"[All Fields] OR "recombineering"[All Fields] OR "recombines"[All Fields] OR "recombining"[All Fields])) AND ("pegylate"[All Fields] OR "pegylated"[All Fields] OR "pegylates"[All Fields] OR "pegylating"[All Fields] OR "pegylation"[All Fields] OR "pegylations"[All | 216 | (coagulation "factor VIII") AND (1980:2020 [pdat]) | Adynovate | Antihemophilic Factor (Recombinant), PEGylated | 2015 |

© 2023 Zhou EW et al. *JAMA Health Forum.*

| | | | | | | |
|---|---|---|---|---|---|---|
| | Fields])) OR (("bax 855"[Supplementary Concept] OR "bax 855"[All Fields] OR "rurioctocog alfa pegol"[All Fields]) AND ("shire"[All Fields] OR "shires"[All Fields])) | | | | | |
| drug373 | "factor ix"[MeSH Terms] OR ("factor"[All Fields] AND "ix"[All Fields]) OR "factor ix"[All Fields] OR "ixinity"[All Fields] OR (("f8 protein human"[Supplementary Concept] OR "f8 protein human"[All Fields] OR "recombinate"[All Fields] OR "recombinant"[All Fields] OR "recombinants"[All Fields] OR "recombinated"[All Fields] OR "recombinates"[All Fields] OR "recombination, genetic"[MeSH Terms] OR ("recombination"[All Fields] AND "genetic"[All Fields]) OR "genetic recombination"[All Fields] OR "recombination"[All Fields] OR "recombinations"[All Fields] OR "recombinational"[All Fields] OR "recombinative"[All Fields] OR "recombine"[All Fields] OR "recombined"[All Fields] OR "recombineered"[All Fields] OR "recombineering"[All Fields] OR "recombines"[All Fields] OR "recombining"[All Fields]) AND ("factor ix"[MeSH Terms] OR ("factor"[All Fields] AND "ix"[All Fields]) OR "factor ix"[All Fields] OR ("coagulation"[All Fields] AND "factor"[All Fields] AND "ix"[All Fields]) OR "coagulation factor ix"[All Fields])) OR ("trenonacog"[All Fields] AND "alfa"[All Fields]) | 217 | (coagulation factor IX) AND (1980:2020 [pdat]) | Ixinity | Recombinant Coagulation factor IX | 2015 |

© 2023 Zhou EW et al. *JAMA Health Forum.*

| | | | | | | |
|---|---|---|---|---|---|---|
| drug374 | "mogamulizumab"[Supplementary Concept] OR "mogamulizumab"[All Fields] OR "poteligeo"[All Fields] OR "mogamulizumab"[Supplementary Concept] OR "mogamulizumab"[All Fields] OR "mogamulizumab"[Supplementary Concept] OR "mogamulizumab"[All Fields] OR "mogamulizumab kpkc"[All Fields] | 216 | (coagulation "factor VIII") AND (1980:2020 [pdat]) | Nuwiq | Simoctocog alfa | 2015 |
| drug375 | "herpesvirus 1, human"[MeSH Terms] OR "human herpesvirus 1"[All Fields] OR "herpesvirus 1 human"[All Fields] OR "imlygic"[All Fields] OR "talimogene laherparepvec"[Supplementary Concept] OR "talimogene laherparepvec"[All Fields] OR "talimogene laherparepvec"[Supplementary Concept] OR "talimogene laherparepvec"[All Fields] | 220 | (Granulocyte-macrophage colony-stimulating factor) AND (1980:2020 [pdat]) | Imlygic | Talimogene laherparepvec | 2015 |
| drug376 | "vonicog alfa"[All Fields] OR "vonvendi"[All Fields] OR (("von willebrand factor"[MeSH Terms] OR ("von"[All Fields] AND "willebrand"[All Fields] AND "factor"[All Fields]) OR "von willebrand factor"[All Fields]) AND ("f8 protein human"[Supplementary Concept] OR "f8 protein human"[All Fields] OR "recombinate"[All Fields] OR "recombinant"[All Fields] OR "recombinants"[All Fields] OR "recombinated"[All Fields] OR "recombinates"[All Fields] OR "recombination, | 200 | (von willebrand factor) AND (1980:2020 [pdat]) | Vonvendi | von Willebrand factor (Recombinant) | 2015 |

© 2023 Zhou EW et al. *JAMA Health Forum.*

| | | | | | | |
|---|---|---|---|---|---|---|
| | genetic"[MeSH Terms] OR ("recombination"[All Fields] AND "genetic"[All Fields]) OR "genetic recombination"[All Fields] OR "recombination"[All Fields] OR "recombinations"[All Fields] OR "recombinational"[All Fields] OR "recombinative"[All Fields] OR "recombine"[All Fields] OR "recombined"[All Fields] OR "recombineered"[All Fields] OR "recombineering"[All Fields] OR "recombines"[All Fields] OR "recombining"[All Fields])) OR ("vonicog"[All Fields] AND "alfa"[All Fields]) | | | | | |
| drug377 | "albutrepenonacog alfa"[Supplementary Concept] OR "albutrepenonacog alfa"[All Fields] OR "idelvion"[All Fields] OR "factor ix"[MeSH Terms] OR ("factor"[All Fields] AND "ix"[All Fields]) OR "factor ix"[All Fields] OR ("albutrepenonacog alfa"[Supplementary Concept] OR "albutrepenonacog alfa"[All Fields]) OR ("albutrepenonacog alfa"[Supplementary Concept] OR "albutrepenonacog alfa"[All Fields]) | 217 | (coagulation factor IX) AND (1980:2020 [pdat]) | Idelvion | Albutrepenonacog alfa | 2016 |
| drug378 | (Kovaltry)or(Recombinant Antihemophilic Factor VIII (Human))or(Factor VIII (recombinant) and Bayer-2) | 216 | (coagulation "factor VIII") AND (1980:2020 [pdat]) | Kovaltry | Recombinant Antihemophilic Factor VIII (Human) | 2016 |

© 2023 Zhou EW et al. *JAMA Health Forum.*

| | | | | | | |
|---|---|---|---|---|---|---|
| drug379 | "afstyla"[All Fields] OR "factor viii"[MeSH Terms] OR ("factor"[All Fields] AND "viii"[All Fields]) OR "factor viii"[All Fields] OR ("Lonoctocog"[All Fields] AND "alfa"[All Fields]) OR ("Lonoctocog"[All Fields] AND "alfa"[All Fields]) | 216 | (coagulation "factor VIII") AND (1980:2020 [pdat]) | Afstyla | Lonoctocog alfa | 2016 |
| drug38 | "carfilzomib"[Supplementary Concept] OR "carfilzomib"[All Fields] OR "kyprolis"[All Fields] OR "carfilzomib"[Supplementary Concept] OR "carfilzomib"[All Fields] OR "carfilzomib"[Supplementary Concept] OR "carfilzomib"[All Fields] | 120 | (proteasome endopeptidase complex[MeSH Terms]) AND (1980:2020 [pdat]) | Kyprolis | Carfilzomib | 2012 |
| drug380 | "factor ix"[MeSH Terms] OR ("factor"[All Fields] AND "ix"[All Fields]) OR "factor ix"[All Fields] OR "rebinyn"[All Fields] OR ("nonacog beta pegol"[Supplementary Concept] OR "nonacog beta pegol"[All Fields]) OR ("nonacog beta pegol"[Supplementary Concept] OR "nonacog beta pegol"[All Fields]) | 217 | (coagulation factor IX) AND (1980:2020 [pdat]) | Rebinyn | Nonacog beta pegol | 2017 |
| drug381 | "tisagenlecleucel"[Supplementary Concept] OR "tisagenlecleucel"[All Fields] OR "kymriah"[All Fields] OR "tisagenlecleucel"[Supplementary Concept] OR "tisagenlecleucel"[All Fields] OR "tisagenlecleucel-t"[All Fields] | 221 | (CD19) AND (1980:2020 [pdat]) | Kymriah | Tisagenlecleucel | 2017 |
| drug382 | "axicabtagene ciloleucel"[Supplementary Concept] OR "axicabtagene ciloleucel"[All Fields] OR "yescarta"[All Fields] OR "axicabtagene ciloleucel"[Supplementary Concept] OR "axicabtagene ciloleucel"[All Fields] OR "axicabtagene ciloleucel"[Supplementary Concept] OR "axicabtagene ciloleucel"[All Fields] | 221 | (CD19) AND (1980:2020 [pdat]) | Yescarta | Axicabtagene ciloleucel | 2017 |
| drug383 | "luxturna"[All Fields] OR "voretigene neparvovec"[All Fields] OR ("voretigene"[All Fields] AND "neparvovec"[All Fields]) OR ("voretigene"[All Fields] AND "neparvovec"[All Fields]) | 222 | (retinoid isomerohydrolase OR RPE65) AND (1980:2020 [pdat]) | Luxturna | Voretigene neparvovec | 2017 |

© 2023 Zhou EW et al. *JAMA Health Forum.*

| | | | | | | |
|---|---|---|---|---|---|---|
| drug384 | "damoctocog alfa pegol"[All Fields] OR "jivi"[All Fields] OR ("Damoctocog"[All Fields] AND "alfa"[All Fields] AND "pegol"[All Fields]) OR ("Damoctocog"[All Fields] AND "alfa"[All Fields] AND "pegol"[All Fields]) | 216 | (coagulation "factor VIII") AND (1980:2020 [pdat]) | Jivi | Damoctocog alfa pegol | 2018 |
| drug385 | "fidaxomicin"[MeSH Terms] OR "fidaxomicin"[All Fields] OR "dificid"[All Fields] OR "fidaxomicin"[MeSH Terms] OR "fidaxomicin"[All Fields] OR "fidaxomicin"[MeSH Terms] OR "fidaxomicin"[All Fields] | 48 | (coagulation factor X) AND (1980:2020 [pdat]) | Andexxa | Coagulation Factor Xa (recombinant), inactivated | 2018 |
| drug386 | "zolgensma"[Supplementary Concept] OR "zolgensma"[All Fields] OR "zolgensma"[All Fields] OR "onasemnogene abeparvovec"[All Fields] OR ("zolgensma"[Supplementary Concept] OR "zolgensma"[All Fields] OR "onasemnogene abeparvovec xioi"[All Fields]) OR ("onasemnogene"[All Fields] AND "abeparvovec"[All Fields]) | 2 | ((((survival motor neuron protein) OR smn) OR smn1 smn2) OR survival of motor neuron 2 protein[MeSH Terms]) AND (1980:2020 [pdat]) | Zolgensma | onasemnogene abeparvovec-xioi | 2019 |
| drug387 | (Esperoct)or(Antihemophilic Factor (recombinant) and GlycoPEGylated-exei)or(turoctocog alfa pegol) | 216 | (coagulation "factor VIII") AND (1980:2020 [pdat]) | Esperoct | Antihemophilic Factor (recombinant), GlycoPEGylated-exei | 2019 |
| drug388 | "rifamycin sv"[Supplementary Concept] OR "rifamycin sv"[All Fields] OR "aemcolo"[All Fields] OR "rifamycin sv"[All Fields] OR "rifamycins"[MeSH Terms] OR "rifamycins"[All Fields] OR ("rifamycin"[All Fields] AND "sv"[All Fields]) OR ("rifamycins"[MeSH Terms] OR "rifamycins"[All Fields] OR "rifamycin"[All Fields]) OR (("rifamycin sv"[Supplementary Concept] OR "rifamycin sv"[All Fields] OR "rifamycin sv"[All Fields] OR "rifamycins"[MeSH Terms] OR "rifamycins"[All Fields] OR ("rifamycin"[All Fields] AND "sv"[All Fields])) AND "Cosmo"[All Fields]) | 0 | | Hyqvia | human immunoglobulin G (IVIg) | 2014 |

© 2023 Zhou EW et al. *JAMA Health Forum.*

| | | | | | | |
|---|---|---|---|---|---|---|
| drug39 | "carglumic acid"[Supplementary Concept] OR "carglumic acid"[All Fields] OR "carbaglu"[All Fields] OR ("carglumic acid"[Supplementary Concept] OR "carglumic acid"[All Fields]) OR (("carglumic acid"[Supplementary Concept] OR "carglumic acid"[All Fields]) AND ("child, orphaned"[MeSH Terms] OR ("child"[All Fields] AND "orphaned"[All Fields]) OR "orphaned child"[All Fields] OR "orphan"[All Fields] OR "orphaned"[All Fields] OR "orphans"[All Fields] OR "orphaning"[All Fields]) AND ("europe"[MeSH Terms] OR "europe"[All Fields] OR "europe s"[All Fields] OR "europes"[All Fields])) | 35 | (carbamoyl-phosphate synthase) AND (1980:2020 [pdat]) | Carbaglu | Carglumic acid | 2010 |
| drug391 | "golodirsen"[Supplementary Concept] OR "golodirsen"[All Fields] OR "vyondys 53"[All Fields] OR "golodirsen"[Supplementary Concept] OR "golodirsen"[All Fields] OR "golodirsen"[Supplementary Concept] OR "golodirsen"[All Fields] | 56 | (dystrophin) AND (1980:2020 [pdat]) | Vyondys 53 | golodirsen | 2019 |
| drug393 | "benznidazol"[All Fields] OR "benzonidazole"[Supplementary Concept] OR "benzonidazole"[All Fields] OR "benznidazole"[All Fields] OR "benznidazol"[All Fields] OR "benzonidazole"[Supplementary Concept] OR "benzonidazole"[All Fields] OR "benznidazole"[All Fields] OR "benznidazol"[All Fields] OR "benzonidazole"[Supplementary Concept] OR "benzonidazole"[All Fields] OR "benznidazole"[All Fields] | 0 | | Vumerity | diroximel fumarate | 2013 |

© 2023 Zhou EW et al. *JAMA Health Forum.*

| | | | | | | |
|---|---|---|---|---|---|---|
| drug4 | "afatinib"[MeSH Terms] OR "afatinib"[All Fields] OR "gilotrif"[All Fields] OR "afatinib"[MeSH Terms] OR "afatinib"[All Fields] OR "afatinib"[MeSH Terms] OR "afatinib"[All Fields] | 59 | (erbb1 OR erbb2) AND (1980:2020 [pdat]) | Gilotrif | Afatinib | 2013 |
| drug40 | "cariprazine"[Supplementary Concept] OR "cariprazine"[All Fields] OR "vraylar"[All Fields] OR "cariprazine"[Supplementary Concept] OR "cariprazine"[All Fields] OR "cariprazine"[Supplementary Concept] OR "cariprazine"[All Fields] | 55 | (dopamine receptor) AND (1980:2020 [pdat]) | Vraylar | Cariprazine | 2015 |
| drug41 | "homoharringtonine"[MeSH Terms] OR "homoharringtonine"[All Fields] OR "synribo"[All Fields] OR ("omacetaxine"[All Fields] AND "mepesuccinate"[All Fields]) OR "omacetaxine mepesuccinate"[All Fields] OR ("homoharringtonine"[MeSH Terms] OR "homoharringtonine"[All Fields] OR ("omacetaxine"[All Fields] AND "mepesuccinate"[All Fields]) OR "omacetaxine mepesuccinate"[All Fields]) OR ("homoharringtonine"[MeSH Terms] OR "homoharringtonine"[All Fields] OR ("omacetaxine"[All Fields] AND "mepesuccinate"[All Fields]) OR "omacetaxine mepesuccinate"[All Fields]) | 239 | (penicillin binding proteins) AND (1980:2020 [pdat]) | Teflaro | Ceftaroline fosamil | 2010 |
| drug42 | (("netupitant"[Supplementary Concept] OR "netupitant"[All Fields] OR "netupitant palosentron drug combination"[Supplementary Concept] OR "netupitant palosentron drug combination"[All Fields] OR "akynzeo"[All Fields] OR "palonosetron"[MeSH Terms] OR "palonosetron"[All Fields] OR "palonosetron s"[All Fields]) AND "IV"[All Fields]) OR (("palonosetron"[MeSH Terms] OR "palonosetron"[All Fields] OR "palonosetron | 239 | (penicillin binding proteins) AND (1980:2020 [pdat]) | Zerbaxa | Ceftolozane plus tazobactam | 2014 |

© 2023 Zhou EW et al. *JAMA Health Forum.*

| | | | | | | |
|---|---|---|---|---|---|---|
| | s"[All Fields]) AND "fosnetupitant"[All Fields]) OR "fosnetupitant"[All Fields] | | | | | |
| drug43 | "ceritinib"[Supplementary Concept] OR "ceritinib"[All Fields] OR "zykadia"[All Fields] OR "ceritinib"[Supplementary Concept] OR "ceritinib"[All Fields] OR "ceritinib"[Supplementary Concept] OR "ceritinib"[All Fields] | 16 | (anaplastic lymphoma kinase) AND (1980:2020 [pdat]) | Zykadia | Ceritinib | 2014 |
| drug44 | "cholic acid"[MeSH Terms] OR ("cholic"[All Fields] AND "acid"[All Fields]) OR "cholic acid"[All Fields] OR "cholbam"[All Fields] OR ("cholic acid"[MeSH Terms] OR ("cholic"[All Fields] AND "acid"[All Fields]) OR "cholic acid"[All Fields]) OR (("cholic acid"[MeSH Terms] OR ("cholic"[All Fields] AND "acid"[All Fields]) OR "cholic acid"[All Fields]) AND "Asklepion"[All Fields]) | 46 | (cholic acid[MeSH Terms]) AND (1980:2020 [pdat]) | Cholbam | Cholic acid | 2015 |
| drug45 | "cobimetinib"[Supplementary Concept] OR "cobimetinib"[All Fields] OR "cotellic"[All Fields] OR "cobimetinib"[Supplementary Concept] OR "cobimetinib"[All Fields] OR "cobimetinib"[Supplementary Concept] OR "cobimetinib"[All Fields] | 96 | (mitogen-activated protein kinase kinases[MeSH Terms]) AND (1980:2020 [pdat]) | Cotellic | Cobimetinib | 2015 |

© 2023 Zhou EW et al. *JAMA Health Forum.*

| | | | | | | |
|---|---|---|---|---|---|---|
| drug46 | "microbial collagenase"[MeSH Terms] OR ("microbial"[All Fields] AND "collagenase"[All Fields]) OR "microbial collagenase"[All Fields] OR ("collagenase"[All Fields] AND "clostridium"[All Fields] AND "histolyticum"[All Fields]) OR "collagenase clostridium histolyticum"[All Fields] OR "xiaflex"[All Fields] OR ("microbial collagenase"[MeSH Terms] OR ("microbial"[All Fields] AND "collagenase"[All Fields]) OR "microbial collagenase"[All Fields] OR ("collagenase"[All Fields] AND "clostridium"[All Fields] AND "histolyticum"[All Fields]) OR "collagenase clostridium histolyticum"[All Fields]) OR ("microbial collagenase"[MeSH Terms] OR ("microbial"[All Fields] AND "collagenase"[All Fields]) OR "microbial collagenase"[All Fields] OR ("collagenase"[All Fields] AND "clostridium"[All Fields] AND "histolyticum"[All Fields]) OR "collagenase clostridium histolyticum"[All Fields]) | 92 | ("microbial collagenase"[MeSH Terms]) AND (1980:2020 [pdat]) | Xiaflex | Collagenase clostridium histolyticum | 2010 |
| drug47 | "crisaborole"[Supplementary Concept] OR "crisaborole"[All Fields] OR "eucrisa"[All Fields] OR "crisaborole"[Supplementary Concept] OR "crisaborole"[All Fields] OR "crisaborole"[Supplementary Concept] OR "crisaborole"[All Fields] | 107 | (phosphodiesterase 4) AND (1980:2020 [pdat]) | Eucrisa | Crisaborole | 2016 |

© 2023 Zhou EW et al. *JAMA Health Forum.*

| | | | | | | |
|---|---|---|---|---|---|---|
| drug48 | "crizotinib"[MeSH Terms] OR "crizotinib"[All Fields] OR "xalkori"[All Fields] OR "crizotinib s"[All Fields] OR "crizotinib"[MeSH Terms] OR "crizotinib"[All Fields] OR "crizotinib s"[All Fields] OR "crizotinib"[MeSH Terms] OR "crizotinib"[All Fields] OR "crizotinib s"[All Fields] | 16 | (anaplastic lymphoma kinase) AND (1980:2020 [pdat]) | Xalkori | Crizotinib | 2011 |
| drug49 | "crofelemer"[Supplementary Concept] OR "crofelemer"[All Fields] OR "fulyzaq"[All Fields] OR ("crofelemer"[Supplementary Concept] OR "crofelemer"[All Fields]) OR (("crofelemer"[Supplementary Concept] OR "crofelemer"[All Fields]) AND "Napo"[All Fields]) | 45 | ("chloride channels"[MeSH Terms]) AND (1980:2020 [pdat]) | Fulyzaq | Crofelemer | 2012 |
| drug5 | "aflibercept"[Supplementary Concept] OR "aflibercept"[All Fields] OR "eylea"[All Fields] OR ("aflibercept"[Supplementary Concept] OR "aflibercept"[All Fields]) OR (("aflibercept"[Supplementary Concept] OR "aflibercept"[All Fields]) AND ("ophthalmic solutions"[Pharmacological Action] OR "ophthalmic solutions"[MeSH Terms] OR ("ophthalmic"[All Fields] AND "solutions"[All Fields]) OR "ophthalmic solutions"[All Fields] OR ("ophthalmic"[All Fields] AND "solution"[All Fields]) OR "ophthalmic solution"[All Fields])) | 153 | (receptors, vascular endothelial growth factor[MeSH Terms]) AND (1980:2020 [pdat]) | Eylea | Aflibercept | 2011 |
| drug50 | "dabigatran"[MeSH Terms] OR "dabigatran"[All Fields] OR "pradaxa"[All Fields] OR ("dabigatran"[All Fields] AND "etexilate"[All Fields]) OR "dabigatran etexilate"[All Fields] OR ("dabigatran"[MeSH Terms] OR "dabigatran"[All Fields] OR "dabigatran s"[All Fields]) OR ("dabigatran"[MeSH Terms] OR "dabigatran"[All Fields] OR ("dabigatran"[All Fields] AND "etexilate"[All Fields]) OR "dabigatran etexilate"[All Fields]) | 141 | (thrombin[MeSH Terms]) AND (1980:2020 [pdat]) | Pradaxa | Dabigatran | 2010 |

© 2023 Zhou EW et al. *JAMA Health Forum.*

| | | | | | | |
|---|---|---|---|---|---|---|
| drug51 | "dabrafenib"[Supplementary Concept] OR "dabrafenib"[All Fields] OR "tafinlar"[All Fields] OR "dabrafenib"[Supplementary Concept] OR "dabrafenib"[All Fields] OR "dabrafenib"[Supplementary Concept] OR "dabrafenib"[All Fields] | 32 | (braf) AND (1980:2020 [pdat]) | Tafinlar | Dabrafenib | 2013 |
| drug52 | "daclatasvir"[Supplementary Concept] OR "daclatasvir"[All Fields] OR "daklinza"[All Fields] OR "daclatasvir"[Supplementary Concept] OR "daclatasvir"[All Fields] OR "daclatasvir"[Supplementary Concept] OR "daclatasvir"[All Fields] | 74 | (hcv ns5a) AND (1980:2020 [pdat]) | Daklinza | Daclatasvir | 2015 |
| drug53 | "dalbavancin"[Supplementary Concept] OR "dalbavancin"[All Fields] OR "dalvance"[All Fields] OR "dalbavancin"[Supplementary Concept] OR "dalbavancin"[All Fields] OR "dalbavancin"[Supplementary Concept] OR "dalbavancin"[All Fields] | 0 | | Dalvance | Dalbavancin | 2014 |
| drug54 | "4 aminopyridine"[MeSH Terms] OR "4 aminopyridine"[All Fields] OR "dalfampridine"[All Fields] OR "ampyra"[All Fields] OR "4 aminopyridine"[MeSH Terms] OR "4 aminopyridine"[All Fields] OR "dalfampridine"[All Fields] OR "4 aminopyridine"[MeSH Terms] OR "4 aminopyridine"[All Fields] OR "dalfampridine"[All Fields] | 112 | (potassium channel) AND (1980:2020 [pdat]) | Ampyra | Dalfampridine | 2010 |
| drug55 | "dapagliflozin propanediol"[All Fields] OR "dapagliflozin"[Supplementary Concept] OR "dapagliflozin"[All Fields] OR "farxiga"[All Fields] OR "dapagliflozin s"[All Fields] OR "dapagliflozin"[Supplementary Concept] OR "dapagliflozin"[All Fields] OR "dapagliflozin s"[All Fields] OR "dapagliflozin"[Supplementary Concept] OR "dapagliflozin"[All Fields] OR "dapagliflozin s"[All Fields] | 135 | (sodium glucose transporter) AND (1980:2020 [pdat]) | Farxiga | Dapagliflozin | 2014 |

© 2023 Zhou EW et al. *JAMA Health Forum.*

| | | | | | | |
|---|---|---|---|---|---|---|
| drug56 | "daratumumab"[Supplementary Concept] OR "daratumumab"[All Fields] OR "darzalex"[All Fields] OR "daratumumab"[Supplementary Concept] OR "daratumumab"[All Fields] OR "daratumumab"[Supplementary Concept] OR "daratumumab"[All Fields] | 40 | (CD38) AND (1980:2020 [pdat]) | Darzalex | Daratumumab | 2015 |
| drug57 | "defibrotide"[Supplementary Concept] OR "defibrotide"[All Fields] OR "defitelio"[All Fields] OR "defibrotide"[Supplementary Concept] OR "defibrotide"[All Fields] OR "defibrotide sodium"[All Fields] OR "defibrotide"[Supplementary Concept] OR "defibrotide"[All Fields] | 0 | | Defitelio | Defibrotide sodium | 2016 |
| drug58 | "denosumab"[MeSH Terms] OR "denosumab"[All Fields] OR "prolia"[All Fields] OR "denosumab s"[All Fields] OR "denosumab"[MeSH Terms] OR "denosumab"[All Fields] OR "denosumab s"[All Fields] OR "denosumab"[MeSH Terms] OR "denosumab"[All Fields] OR "denosumab s"[All Fields] | 144 | (tnfsf11) AND (1980:2020 [pdat]) | Prolia | Denosumab | 2010 |
| drug59 | "deoxycholates"[All Fields] OR "deoxycholic acid"[MeSH Terms] OR ("deoxycholic"[All Fields] AND "acid"[All Fields]) OR "deoxycholic acid"[All Fields] OR "deoxycholate"[All Fields] OR "kybella"[All Fields] OR "deoxycholic"[All Fields] OR ("deoxycholic acid"[MeSH Terms] OR ("deoxycholic"[All Fields] AND "acid"[All Fields]) OR "deoxycholic acid"[All Fields]) OR ("deoxycholic acid"[MeSH Terms] OR ("deoxycholic"[All Fields] AND "acid"[All Fields]) OR "deoxycholic acid"[All Fields]) | 0 | | Kybella | Deoxycholic acid | 2015 |

© 2023 Zhou EW et al. *JAMA Health Forum.*

| | | | | | | |
|---|---|---|---|---|---|---|
| drug6 | "rglp 1 protein"[Supplementary Concept] OR "rglp 1 protein"[All Fields] OR "albiglutide"[All Fields] OR "tanzeum"[All Fields] OR "rglp 1 protein"[Supplementary Concept] OR "rglp 1 protein"[All Fields] OR "albiglutide"[All Fields] OR "rglp 1 protein"[Supplementary Concept] OR "rglp 1 protein"[All Fields] OR "albiglutide"[All Fields] | 67 | (glucagon-like peptide 1) AND (1980:2020 [pdat]) | Tanzeum | Albiglutide | 2014 |
| drug60 | "Natazia"[All Fields] OR (("estradiol"[MeSH Terms] OR "estradiol"[All Fields] OR ("oestradiol"[All Fields] AND "valerate"[All Fields]) OR "oestradiol valerate"[All Fields]) AND ("dienogest"[Supplementary Concept] OR "dienogest"[All Fields])) OR (("dienogest"[Supplementary Concept] OR "dienogest"[All Fields]) AND ("estradiol"[MeSH Terms] OR "estradiol"[All Fields] OR ("estradiol"[All Fields] AND "valerate"[All Fields]) OR "estradiol valerate"[All Fields]) AND ("sequential"[All Fields] OR "sequentially"[All Fields] OR "sequentials"[All Fields]) AND "Bayer"[All Fields] AND "Schering"[All Fields]) | 62 | (estrogen receptor) AND (1980:2020 [pdat]) | Natazia | Oestradiol valerate, dienogest | 2010 |
| drug61 | "dimethyl fumarate"[MeSH Terms] OR ("dimethyl"[All Fields] AND "fumarate"[All Fields]) OR "dimethyl fumarate"[All Fields] OR "tecfidera"[All Fields] OR ("dimethyl fumarate"[MeSH Terms] OR ("dimethyl"[All Fields] AND "fumarate"[All Fields]) OR "dimethyl fumarate"[All Fields]) OR ("dimethyl fumarate"[MeSH Terms] OR ("dimethyl"[All Fields] AND "fumarate"[All Fields]) OR "dimethyl fumarate"[All Fields]) | 100 | (nrf2 OR NFE2l2) AND (1980:2020 [pdat]) | Tecfidera | Dimethyl fumarate | 2013 |

© 2023 Zhou EW et al. *JAMA Health Forum.*

| | | | | | | |
|---|---|---|---|---|---|---|
| drug62 | "dinutuximab"[Supplementary Concept] OR "dinutuximab"[All Fields] OR "unituxin"[All Fields] OR "dinutuximab"[Supplementary Concept] OR "dinutuximab"[All Fields] OR "dinutuximab"[Supplementary Concept] OR "dinutuximab"[All Fields] | 6 | ((glycolipid gd2) OR disialoganglioside gd2) AND (1980:2020 [pdat]) | Unituxin | Dinutuximab | 2015 |
| drug63 | "dolutegravir"[Supplementary Concept] OR "dolutegravir"[All Fields] OR "tivicay"[All Fields] OR "dolutegravir"[Supplementary Concept] OR "dolutegravir"[All Fields] OR "dolutegravir"[Supplementary Concept] OR "dolutegravir"[All Fields] | 183 | (HIV integrase) AND (1980:2020 [pdat]) | Tivicay | Dolutegravir | 2013 |
| drug64 | "droxidopa"[MeSH Terms] OR "droxidopa"[All Fields] OR "northera"[All Fields] OR "droxidopa"[MeSH Terms] OR "droxidopa"[All Fields] OR "droxidopa"[MeSH Terms] OR "droxidopa"[All Fields] | 11 | (adrenergic receptor) AND (1980:2020 [pdat]) | Northera | Droxidopa | 2014 |
| drug65 | "dulaglutide"[Supplementary Concept] OR "dulaglutide"[All Fields] OR "trulicity"[All Fields] OR "dulaglutide"[Supplementary Concept] OR "dulaglutide"[All Fields] OR "dulaglutide"[Supplementary Concept] OR "dulaglutide"[All Fields] | 67 | (glucagon-like peptide 1) AND (1980:2020 [pdat]) | Trulicity | Dulaglutide | 2014 |
| drug66 | "edoxaban"[Supplementary Concept] OR "edoxaban"[All Fields] OR "savaysa"[All Fields] OR "edoxaban"[Supplementary Concept] OR "edoxaban"[All Fields] OR "edoxaban"[Supplementary Concept] OR "edoxaban"[All Fields] | 48 | (coagulation factor X) AND (1980:2020 [pdat]) | Savaysa | Edoxaban | 2015 |
| drug67 | "sodium zirconium cyclosilicate"[Supplementary Concept] OR "sodium zirconium cyclosilicate"[All Fields] OR "lokelma"[All Fields] OR "sodium zirconium cyclosilicate"[Supplementary Concept] OR "sodium zirconium cyclosilicate"[All Fields] OR "sodium zirconium cyclosilicate"[Supplementary Concept] OR "sodium zirconium cyclosilicate"[All Fields] | 236 | (sterol 14-demethylase) AND (1980:2020 [pdat]) | Jublia | Efinaconazole | 2014 |

© 2023 Zhou EW et al. *JAMA Health Forum.*

| | | | | | | |
|---|---|---|---|---|---|---|
| drug68 | "2 pyrrolidin 2 yl 5 2 4 5 pyrrolidin 2 yl 1h imidazol 2 yl phenyl benzofuran 5 yl 1h imidazole"[Supplementary Concept] OR "2 pyrrolidin 2 yl 5 2 4 5 pyrrolidin 2 yl 1h imidazol 2 yl phenyl benzofuran 5 yl 1h imidazole"[All Fields] OR "elbasvir"[All Fields] OR "elbasvir grazoprevir drug combination"[Supplementary Concept] OR "elbasvir grazoprevir drug combination"[All Fields] OR "zepatier"[All Fields] OR "grazoprevir"[Supplementary Concept] OR "grazoprevir"[All Fields] OR (("2 pyrrolidin 2 yl 5 2 4 5 pyrrolidin 2 yl 1h imidazol 2 yl phenyl benzofuran 5 yl 1h imidazole"[Supplementary Concept] OR "2 pyrrolidin 2 yl 5 2 4 5 pyrrolidin 2 yl 1h imidazol 2 yl phenyl benzofuran 5 yl 1h imidazole"[All Fields] OR "elbasvir"[All Fields]) AND ("grazoprevir"[Supplementary Concept] OR "grazoprevir"[All Fields])) OR (("2 pyrrolidin 2 yl 5 2 4 5 pyrrolidin 2 yl 1h imidazol 2 yl phenyl benzofuran 5 yl 1h imidazole"[Supplementary Concept] OR "2 pyrrolidin 2 yl 5 2 4 5 pyrrolidin 2 yl 1h imidazol 2 yl phenyl benzofuran 5 yl 1h imidazole"[All Fields] OR "elbasvir"[All Fields]) AND ("grazoprevir"[Supplementary Concept] OR "grazoprevir"[All Fields])) | 71 | (hcv ns3 OR hcv ns4a) AND (1980:2020 [pdat]) | Zepatier | Elbasvir, grazoprevir | 2016 |

© 2023 Zhou EW et al. *JAMA Health Forum.*

| | | | | | | |
|---|---|---|---|---|---|---|
| drug69 | "eliglustat"[Supplementary Concept] OR "eliglustat"[All Fields] OR "cerdelga"[All Fields] OR "eliglustat"[Supplementary Concept] OR "eliglustat"[All Fields] OR "eliglustat"[Supplementary Concept] OR "eliglustat"[All Fields] | 43 | (Ceramide glucosyltransferase OR glucosylceramide synthase) AND (1980:2020 [pdat]) | Cerdelga | Eliglustat | 2014 |
| drug7 | "alcaftadine"[Supplementary Concept] OR "alcaftadine"[All Fields] OR "lastacaft"[All Fields] OR "alcaftadine"[Supplementary Concept] OR "alcaftadine"[All Fields] OR "alcaftadine"[Supplementary Concept] OR "alcaftadine"[All Fields] | 70 | (H1 histamine receptor[MeSH Terms]) AND (1980:2020 [pdat]) | Lastacaft | Alcaftadine | 2010 |
| drug70 | "galns protein human"[Supplementary Concept] OR "galns protein human"[All Fields] OR "elosulfase alfa"[All Fields] OR "vimizim"[All Fields] OR "galns protein human"[Supplementary Concept] OR "galns protein human"[All Fields] OR "elosulfase alfa"[All Fields] OR "galns protein human"[Supplementary Concept] OR "galns protein human"[All Fields] OR "elosulfase alfa"[All Fields] | 97 | (N Acetylgalactosamine 6 sulfatase) AND (1980:2020 [pdat]) | Vimizim | Elosulfase alfa | 2014 |

© 2023 Zhou EW et al. *JAMA Health Forum.*

| | | | | | | |
|---|---|---|---|---|---|---|
| drug71 | "elotuzumab"[Supplementary Concept] OR "elotuzumab"[All Fields] OR "empliciti"[All Fields] OR "elotuzumab"[Supplementary Concept] OR "elotuzumab"[All Fields] OR "elotuzumab"[Supplementary Concept] OR "elotuzumab"[All Fields] | 133 | (slamf7) AND (1980:2020 [pdat]) | Empliciti | Elotuzumab | 2015 |
| drug72 | "eluxadoline"[Supplementary Concept] OR "eluxadoline"[All Fields] OR "viberzi"[All Fields] OR "eluxadoline"[Supplementary Concept] OR "eluxadoline"[All Fields] OR "eluxadoline"[Supplementary Concept] OR "eluxadoline"[All Fields] | 128 | (receptors, opioid[MeSH Terms]) AND (1980:2020 [pdat]) | Viberzi | Eluxadoline | 2015 |
| drug73 | "jtk 303"[Supplementary Concept] OR "jtk 303"[All Fields] OR "elvitegravir"[All Fields] OR "racivir"[Supplementary Concept] OR "racivir"[All Fields] OR "emtricitabine"[All Fields] OR "emtricitabine"[MeSH Terms] OR "cobicistat"[MeSH Terms] OR "cobicistat"[All Fields] OR "elvitegravir, cobicistat, emtricitabine, tenofovir disoproxil fumarate drug combination"[MeSH Terms] OR ("elvitegravir"[All Fields] AND "cobicistat"[All Fields] AND "emtricitabine"[All Fields] AND "tenofovir"[All Fields] AND "disoproxil"[All Fields] AND "fumarate"[All Fields] AND "drug"[All Fields] AND "combination"[All Fields]) OR "tenofovir disoproxil fumarate drug combination emtricitabine cobicistat elvitegravir"[All Fields] OR "genvoya"[All Fields] OR "emtricitabin"[All Fields] OR "tenofovir alafenamide"[All Fields] OR (("jtk 303"[Supplementary Concept] OR "jtk 303"[All Fields] OR "elvitegravir"[All Fields]) AND ("cobicistat"[MeSH Terms] OR "cobicistat"[All Fields]) AND ("racivir"[Supplementary Concept] OR "racivir"[All Fields] OR "emtricitabine"[All Fields] OR "emtricitabine"[MeSH Terms] OR | 228 | (HIV integrase or CYP3A) AND (1980:2020 [pdat]) | Genvoya | Elvitegravir, cobicistat, emtricitabine, tenofovir | 2015 |

© 2023 Zhou EW et al. *JAMA Health Forum.*

| | | | | | | |
|---|---|---|---|---|---|---|
| | "emtricitabin"[All Fields]) AND ("tenofovir"[MeSH Terms] OR "tenofovir"[All Fields])) | | | | | |
| drug74 | "empagliflozin"[Supplementary Concept] OR "empagliflozin"[All Fields] OR "jardiance"[All Fields] OR "empagliflozin"[Supplementary Concept] OR "empagliflozin"[All Fields] OR "empagliflozin"[Supplementary Concept] OR "empagliflozin"[All Fields] | 135 | (sodium glucose transporter) AND (1980:2020 [pdat]) | Jardiance | Empagliflozin | 2014 |
| drug75 | "enzalutamide"[Supplementary Concept] OR "enzalutamide"[All Fields] OR "xtandi"[All Fields] OR "enzalutamide"[Supplementary Concept] OR "enzalutamide"[All Fields] OR "enzalutamide"[Supplementary Concept] OR "enzalutamide"[All Fields] | 17 | (androgen receptor) AND (1980:2020 [pdat]) | Xtandi | Enzalutamide | 2012 |
| drug76 | "eribulin"[Supplementary Concept] OR "eribulin"[All Fields] OR "halaven"[All Fields] OR "eribulin"[Supplementary Concept] OR "eribulin"[All Fields] OR "eribulin"[Supplementary Concept] OR "eribulin"[All Fields] OR "eribulin mesylate"[All Fields] | 94 | (microtubule assembly) AND (1980:2020 [pdat]) | Halaven | Eribulin | 2010 |

© 2023 Zhou EW et al. *JAMA Health Forum.*

| | | | | | | |
|---|---|---|---|---|---|---|
| drug77 | "eslicarbazepine acetate"[Supplementary Concept] OR "eslicarbazepine acetate"[All Fields] OR "aptiom"[All Fields] OR "eslicarbazepine"[Supplementary Concept] OR "eslicarbazepine"[All Fields] OR "eslicarbazepine acetate"[Supplementary Concept] OR "eslicarbazepine acetate"[All Fields] OR "eslicarbazepine acetate"[Supplementary Concept] OR "eslicarbazepine acetate"[All Fields] | 152 | (voltage gated sodium channel) AND (1980:2020 [pdat]) | Aptiom | Eslicarbazepine acetate | 2013 |
| drug78 | "eteplirsen"[Supplementary Concept] OR "eteplirsen"[All Fields] OR "exondys 51"[All Fields] OR "eteplirsen"[Supplementary Concept] OR "eteplirsen"[All Fields] OR "eteplirsen"[Supplementary Concept] OR "eteplirsen"[All Fields] | 56 | (dystrophin) AND (1980:2020 [pdat]) | Exondys 51 | Eteplirsen | 2016 |
| drug79 | "eicosapentaenoic acid ethyl ester"[Supplementary Concept] OR "eicosapentaenoic acid ethyl ester"[All Fields] OR "icosapent ethyl"[All Fields] OR "vascepa"[All Fields] OR ("eicosapentaenoic acid ethyl ester"[Supplementary Concept] OR "eicosapentaenoic acid ethyl ester"[All Fields] OR "ethyl eicosapentaenoic acid"[All Fields]) OR (("ethyl"[All Fields] OR "ethylate"[All Fields] OR "ethylated"[All Fields] OR "ethylates"[All Fields] OR "ethylating"[All Fields] OR "ethylation"[All Fields] OR "ethylations"[All Fields] OR "ethyls"[All Fields]) AND "icosapentate"[All Fields] AND "Amarin"[All Fields]) | 0 | | Vascepa | Ethyl eicosapentaenoic acid | 2012 |

© 2023 Zhou EW et al. *JAMA Health Forum.*

| | | | | | | |
|---|---|---|---|---|---|---|
| drug8 | "alectinib"[Supplementary Concept] OR "alectinib"[All Fields] OR "alecensa"[All Fields] OR ("alectinib"[Supplementary Concept] OR "alectinib"[All Fields]) OR (("alectinib"[Supplementary Concept] OR "alectinib"[All Fields]) AND ("hydrochlorid"[All Fields] OR "hydrochloride"[All Fields] OR "hydrochlorides"[All Fields])) | 16 | (anaplastic lymphoma kinase) AND (1980:2020 [pdat]) | Alecensa | Alectinib | 2015 |
| drug80 | "evolocumab"[Supplementary Concept] OR "evolocumab"[All Fields] OR "repatha"[All Fields] OR "evolocumab"[Supplementary Concept] OR "evolocumab"[All Fields] OR "evolocumab"[Supplementary Concept] OR "evolocumab"[All Fields] | 117 | (proprotein convertase subtilisin kexin type 9) AND (1980:2020 [pdat]) | Repatha | Evolocumab | 2015 |
| drug81 | "ezogabine"[Supplementary Concept] OR "ezogabine"[All Fields] OR "potiga"[All Fields] OR "ezogabine"[Supplementary Concept] OR "ezogabine"[All Fields] OR "ezogabine"[Supplementary Concept] OR "ezogabine"[All Fields] | 113 | (potassium voltage-gated channel) AND (1980:2020 [pdat]) | Potiga | Ezogabine | 2011 |
| drug82 | "tavaborole"[Supplementary Concept] OR "tavaborole"[All Fields] OR "kerydin"[All Fields] OR "tavaborole"[Supplementary Concept] OR "tavaborole"[All Fields] OR "tavaborole"[Supplementary Concept] OR "tavaborole"[All Fields] | 247 | (bacterial rna polymerase) AND (1980:2020 [pdat]) | Dificid | Fidaxomicin | 2011 |
| drug83 | "omadacycline"[Supplementary Concept] OR "omadacycline"[All Fields] OR "nuzyra"[All Fields] OR "omadacycline"[Supplementary Concept] OR "omadacycline"[All Fields] OR "omadacycline"[Supplementary Concept] OR "omadacycline"[All Fields] | 244 | (DNA gyrase OR "topoisomerase IV") AND (1980:2020 [pdat]) | Xtoro | Finafloxacin | 2014 |

© 2023 Zhou EW et al. *JAMA Health Forum.*

| | | | | | | |
|---|---|---|---|---|---|---|
| drug84 | "fingolimod hydrochloride"[MeSH Terms] OR ("fingolimod"[All Fields] AND "hydrochloride"[All Fields]) OR "fingolimod hydrochloride"[All Fields] OR "fingolimod"[All Fields] OR "gilenya"[All Fields] OR "fingolimod s"[All Fields] OR ("fingolimod hydrochloride"[MeSH Terms] OR ("fingolimod"[All Fields] AND "hydrochloride"[All Fields]) OR "fingolimod hydrochloride"[All Fields] OR "fingolimod"[All Fields] OR "fingolimod s"[All Fields]) OR ("fingolimod hydrochloride"[MeSH Terms] OR ("fingolimod"[All Fields] AND "hydrochloride"[All Fields]) OR "fingolimod hydrochloride"[All Fields]) | 126 | (receptors, lysosphingolipid[MeSH Terms]) AND (1980:2020 [pdat]) | Gilenya | Fingolimod | 2010 |
| drug85 | "flibanserin"[Supplementary Concept] OR "flibanserin"[All Fields] OR "addyi"[All Fields] OR "flibanserin"[Supplementary Concept] OR "flibanserin"[All Fields] OR "flibanserin"[Supplementary Concept] OR "flibanserin"[All Fields] | 3 | ((dopamine receptor) OR serotonin receptor) AND (1980:2020 [pdat]) | Addyi | Flibanserin | 2015 |
| drug86 | "gabapentin"[MeSH Terms] OR "gabapentin"[All Fields] OR "gabapentine"[All Fields] OR "gabapentin s"[All Fields] OR "horizant"[All Fields] OR "1 alpha isobutanoyloxyethoxy carbonyl aminomethyl 1 cyclohexaneacetic acid"[Supplementary Concept] OR "1 alpha isobutanoyloxyethoxy carbonyl aminomethyl 1 cyclohexaneacetic acid"[All Fields] OR "gabapentin enacarbil"[All Fields] OR "1 alpha isobutanoyloxyethoxy carbonyl aminomethyl 1 cyclohexaneacetic acid"[Supplementary Concept] OR "1 alpha isobutanoyloxyethoxy carbonyl aminomethyl 1 | 151 | (voltage gated calcium channel) AND (1980:2020 [pdat]) | Horizant | Gabapentin enacarbil | 2011 |

© 2023 Zhou EW et al. *JAMA Health Forum.*

| | | | | | | |
|---|---|---|---|---|---|---|
| | cyclohexaneacetic acid"[All Fields] OR "gabapentin enacarbil"[All Fields] | | | | | |
| drug87 | "glucarpidase"[Supplementary Concept] OR "glucarpidase"[All Fields] OR "gamma glutamyl hydrolase"[MeSH Terms] OR ("gamma glutamyl"[All Fields] AND "hydrolase"[All Fields]) OR "gamma glutamyl hydrolase"[All Fields] OR "voraxaze"[All Fields] OR ("glucarpidase"[Supplementary Concept] OR "glucarpidase"[All Fields] OR "gamma glutamyl hydrolase"[MeSH Terms] OR ("gamma glutamyl"[All Fields] AND "hydrolase"[All Fields]) OR "gamma glutamyl hydrolase"[All Fields]) OR (("glucarpidase"[Supplementary Concept] OR "glucarpidase"[All Fields] OR "gamma glutamyl hydrolase"[MeSH Terms] OR ("gamma glutamyl"[All Fields] AND "hydrolase"[All Fields]) OR "gamma glutamyl hydrolase"[All Fields]) AND "HPA"[All Fields]) | 36 | (carboxypeptidase G) AND (1980:2020 [pdat]) | Voraxaze | Glucarpidase | 2012 |
| drug88 | "ibrutinib"[Supplementary Concept] OR "ibrutinib"[All Fields] OR "imbruvica"[All Fields] OR "ibrutinib s"[All Fields] OR "ibrutinib"[Supplementary Concept] OR "ibrutinib"[All Fields] OR "ibrutinib s"[All Fields] OR "ibrutinib"[Supplementary Concept] OR "ibrutinib"[All Fields] OR "ibrutinib s"[All Fields] | 33 | (bruton's tyrosine kinase) AND (1980:2020 [pdat]) | Imbruvica | Ibrutinib | 2013 |
| drug89 | "firazyr"[All Fields] OR "icatibant"[Supplementary Concept] OR "icatibant"[All Fields] OR "icatibant"[Supplementary Concept] OR "icatibant"[All Fields] OR | 31 | (bradykinin receptor B2) AND (1980:2020 [pdat]) | Firazyr | Icatibant | 2011 |

© 2023 Zhou EW et al. *JAMA Health Forum.*

| | | | | | | |
|---|---|---|---|---|---|---|
| | "icatibant"[Supplementary Concept] OR "icatibant"[All Fields] | | | | | |
| drug9 | "oritavancin"[Supplementary Concept] OR "oritavancin"[All Fields] OR "oritavancin s"[All Fields] OR "oritavancin"[Supplementary Concept] OR "oritavancin"[All Fields] OR "oritavancin s"[All Fields] | 13 | (alpha glucosidase FAILED TIME) AND (1980:2020 [pdat]) | Lumizyme | Alglucosidase alfa | 2010 |
| drug90 | "idarucizumab"[Supplementary Concept] OR "idarucizumab"[All Fields] OR "praxbind"[All Fields] OR "idarucizumab"[Supplementary Concept] OR "idarucizumab"[All Fields] OR "idarucizumab"[Supplementary Concept] OR "idarucizumab"[All Fields] | 51 | (Dabigatran) AND (1980:2020 [pdat]) | Praxbind | Idarucizumab | 2015 |
| drug91 | "idelalisib"[Supplementary Concept] OR "idelalisib"[All Fields] OR "zydelig"[All Fields] OR "idelalisib"[Supplementary Concept] OR "idelalisib"[All Fields] OR "idelalisib"[Supplementary Concept] OR "idelalisib"[All Fields] | 109 | (Phosphoinositide 3-kinase OR PI-3 kinase) AND (1980:2020 [pdat]) | Zydelig | Idelalisib | 2014 |
| drug92 | "raxibacumab"[Supplementary Concept] OR "raxibacumab"[All Fields] OR "raxibacumab"[Supplementary Concept] OR "raxibacumab"[All Fields] OR "raxibacumab"[Supplementary Concept] OR "raxibacumab"[All Fields] | 30 | (botulinum toxin A) AND (1980:2020 [pdat]) | Xeomin | IncobotulinumtoxinA | 2010 |
| drug93 | "indacaterol"[Supplementary Concept] OR "indacaterol"[All Fields] OR "arcapta neohaler"[All Fields] OR ("indacaterol"[Supplementary Concept] OR "indacaterol"[All Fields]) OR (("indacaterol"[Supplementary Concept] OR "indacaterol"[All Fields]) AND ("maleat"[All Fields] OR "maleates"[MeSH Terms] OR "maleates"[All Fields] OR "maleic acid"[Supplementary Concept] OR "maleic acid"[All Fields] OR "maleate"[All Fields])) | 28 | (beta-2-adrenergic receptor) AND (1980:2020 [pdat]) | Arcapta neohaler | Indacaterol | 2011 |

© 2023 Zhou EW et al. *JAMA Health Forum.*

| | | | | | | |
|---|---|---|---|---|---|---|
| drug94 | "3 ingenyl angelate"[Supplementary Concept] OR "3 ingenyl angelate"[All Fields] OR "ingenol mebutate"[All Fields] OR "picato"[All Fields] OR "3 ingenyl angelate"[Supplementary Concept] OR "3 ingenyl angelate"[All Fields] OR "ingenol mebutate"[All Fields] OR "3 ingenyl angelate"[Supplementary Concept] OR "3 ingenyl angelate"[All Fields] OR "ingenol mebutate"[All Fields] | 122 | (protein kinase c-delta[MeSH Terms]) AND (1980:2020 [pdat]) | Picato | Ingenol mebutate | 2012 |
| drug95 | "insulin degludec"[Supplementary Concept] OR "insulin degludec"[All Fields] OR "tresiba"[All Fields] OR "insulin degludec"[Supplementary Concept] OR "insulin degludec"[All Fields] OR "insulin degludec"[Supplementary Concept] OR "insulin degludec"[All Fields] | 80 | (insulin receptor) AND (1980:2020 [pdat]) | Tresiba | Insulin degludec | 2015 |
| drug96 | "ipilimumab"[MeSH Terms] OR "ipilimumab"[All Fields] OR "yervoy"[All Fields] OR "ipilimumab"[MeSH Terms] OR "ipilimumab"[All Fields] OR "ipilimumab"[MeSH Terms] OR "ipilimumab"[All Fields] | 50 | (cytotoxic T-lymphocyte-associated protein 4 OR CTLA-4) AND (1980:2020 [pdat]) | Yervoy | Ipilimumab | 2011 |
| drug97 | "elapegademase"[Supplementary Concept] OR "elapegademase"[All Fields] OR "revcovi"[All Fields] OR "elapegademase"[Supplementary Concept] OR "elapegademase"[All Fields] OR "elapegademase"[Supplementary Concept] OR "elapegademase"[All Fields] | 236 | (sterol 14-demethylase) AND (1980:2020 [pdat]) | Cresemba | Isavuconazonium | 2015 |
| drug98 | "eravacycline"[Supplementary Concept] OR "eravacycline"[All Fields] OR "xerava"[All Fields] OR "eravacycline"[Supplementary Concept] OR "eravacycline"[All Fields] OR "eravacycline"[Supplementary Concept] OR "eravacycline"[All Fields] | 79 | (hyperpolarization-activated cyclic nucleotide-gated channels) AND (1980:2020 [pdat]) | Corlanor | Ivabradine | 2015 |

© 2023 Zhou EW et al. *JAMA Health Forum.*

| | | | | | | |
|---|---|---|---|---|---|---|
| drug99 | "ivacaftor"[Supplementary Concept] OR "ivacaftor"[All Fields] OR "kalydeco"[All Fields] OR "ivacaftor"[Supplementary Concept] OR "ivacaftor"[All Fields] OR "ivacaftor"[Supplementary Concept] OR "ivacaftor"[All Fields] | 44 | (CFTR) AND (1980:2020 [pdat]) | Kalydeco | Ivacaftor | 2012 |

© 2023 Zhou EW et al. *JAMA Health Forum.*

## eTable 2. Estimated Sensitivity and Specificity of Discovered Developmental and Clinical PMIDs

| | | |
|---|---|---|
| | True Positive<br>184 | False Positive<br>16 |
| | False Negative<br>187 | True Negative<br>13 |
| **A. Phased development research PMIDs** | | |
| Sensitivity | 93.4% | TP/(TP+FN) |
| Specificity | 92.1% | TN/(FP+TN) |
| Precision | 92.0% | TP/(TP+FP) |
| Accuracy | 93.5% | (TP+TN)/(TP+TN+FP+FN) |
| **B. Overall clinical trial PMIDs** | | |
| Sensitivity | 85.7% | TP/(TP+FN) |
| Specificity | 99.6% | TN/(FP+TN) |
| Precision | 91.0% | TP/(TP+FP) |
| Accuracy | 99.1% | (TP+TN)/(TP+TN+FP+FN) |

A. Sensitivity and specificity were calculated by manual validation on a 400 randomized PMID sample set by two independent reviewers. B. Overall Clinical sensitivity and specificity were extrapolated by manual validation results.

© 2023 Zhou EW et al. *JAMA Health Forum.*

## eTable 3. Overall NIH Per-Phase Average Investment Comparison With Industry

| | NIH (240 set unique Project Year costs in millions 2018 USD)[a] | Wouters[b] | NIH/Wouters | DiMasi (2018 USD - Approved drugs only)[c] | NIH/DiMasi |
|---|---|---|---|---|---|
| **P1** | $13.9 | $54.9 | 25.3% | $56.6 | 24.6% |
| **P2** | $22.2 | $103.6 | 21.4% | $95.4 | 23.2% |
| **P3** | $12.9 | $298.3 | 4.3% | $346.9 | 3.7% |
| **Total P1-3** | $49.0 | $456.7 | 10.7% | $498.9 | 9.8% |

a - Average per phase cost of all 240 NIH development research funded drugs; b - Data of 60 matched drug set from Wouters et al[26] as shown in their eTable 4 inflation-adjusted to 2018 USD; c - Data from 19 drugs with approval from Table B.2 of DiMasi et al[22]

© 2023 Zhou EW et al. *JAMA Health Forum.*

**eTable 4. Total NIH Development Research Investment for Each Drug by Phase.** Total number of drugs (n=240). Phase 1 (n=105), Phase 2 (n=158), Phase 3 (n=198), Phase 4 (n=55), NCT (others) (n=22).

| Drug ID | Brand | Phase 1 | Phase 2 | Phase 3 | Phase 4 | NCT (others) | Total Phase 1-3 | Total NIH development research investment |
|---|---|---|---|---|---|---|---|---|
| drug192 | Actemra | $8,594,440 | $41,445,112 | $40,796,071 | $8,499,181 | $4,620,343 | $90,835,623 | $103,955,147 |
| drug359 | Adakveo | | $1,565,840 | | | | $1,565,840 | $1,565,840 |
| drug31 | Adcetris | $45,727,491 | $106,720,573 | $165,949,414 | | | $318,397,478 | $318,397,478 |
| drug85 | Addyi | | | $1,243,633 | | | $1,243,633 | $1,243,633 |
| drug164 | Adempas | | $128,079 | | | | $128,079 | $128,079 |
| drug110 | Adlyxin | | | $7,692,040 | $679,922 | | $7,692,040 | $8,371,962 |
| drug261 | Aemcolo | $95,158,539 | $32,733,598 | $348,792,328 | $3,186,589 | | $476,684,465 | $479,871,054 |
| drug8 | Alecensa | | $9,329,864 | $22,416,089 | | | $31,745,953 | $31,745,953 |
| drug216 | Aliqopa | $7,789,374 | | | | | $7,789,374 | $7,789,374 |
| drug54 | Ampyra | | | $802,159 | | | $802,159 | $802,159 |
| drug385 | Andexxa | | | $147,380 | | | $147,380 | $147,380 |
| drug265 | Annovera | | | $529,512 | | | $529,512 | $529,512 |
| drug93 | Arcapta neohaler | | | $259,257 | | | $259,257 | $259,257 |
| drug188 | Aubagio | | | $74,273 | | | $74,273 | $74,273 |
| drug218 | Austedo | | | $23,188,964 | | | $23,188,964 | $23,188,964 |
| drug19 | Avycaz | | | $2,943,714 | $9,710,879 | | $2,943,714 | $12,654,593 |
| drug323 | Balversa | | | $2,919,834 | | | $2,919,834 | $2,919,834 |
| drug219 | Bavencio | $7,860,138 | $26,246,024 | $43,410,288 | | | $77,516,450 | $77,516,450 |
| drug26 | Beleodaq | $11,091,458 | $77,965,210 | $29,204,120 | | | $118,260,788 | $118,260,788 |
| drug180 | Belsomra | $311,148 | | | | | $311,148 | $311,148 |
| drug112 | Belviq | $1,848,475 | $1,967,675 | | $2,470,140 | $1,211,702 | $3,816,150 | $7,497,992 |
| drug25 | Benlysta | $11,279,640 | $18,331,861 | $47,479,639 | | | $77,091,140 | $77,091,140 |
| drug221 | Benznidazole | | $2,505,252 | | | | $2,505,252 | $2,505,252 |

© 2023 Zhou EW et al. *JAMA Health Forum.*

| | | | | | | | | |
|---|---|---|---|---|---|---|---|---|
| drug222 | Besponsa | | $202,024 | $60,352,053 | | | $60,554,077 | $60,554,077 |
| drug28 | Blincyto | | $10,629,472 | $39,923,483 | | | $50,552,955 | $50,552,955 |
| drug30 | Bosulif | | $16,276,547 | $76,091,644 | | | $92,368,191 | $92,368,191 |
| drug268 | Braftovi | | $625,442 | $13,442,482 | | | $14,067,924 | $14,067,924 |
| drug190 | Brilinta | | | $12,410,917 | $7,868,565 | $16,932,283 | $12,410,917 | $37,211,765 |
| drug33 | Briviact | $9,092,722 | | $8,352,303 | | | $17,445,025 | $17,445,025 |
| drug225 | Calquence | | $32,523,506 | $43,091,212 | | | $75,614,718 | $75,614,718 |
| drug199 | Caprelsa | $38,529,389 | $84,095,598 | $109,598,406 | | | $232,223,393 | $232,223,393 |
| drug69 | Cerdelga | | | $11,810,285 | | | $11,810,285 | $11,810,285 |
| drug44 | Cholbam | | | $10,306,457 | | $12,491,087 | $10,306,457 | $22,797,544 |
| drug35 | Cometriq | $31,379,418 | $198,974,178 | $341,493,454 | | | $571,847,050 | $571,847,050 |
| drug269 | Copiktra | $1,884,265 | | $49,061,712 | | | $50,945,977 | $50,945,977 |
| drug98 | Corlanor | | $1,997,407 | | | | $1,997,407 | $1,997,407 |
| drug172 | Cosentyx | | $1,265,186 | | $4,111,614 | | $1,265,186 | $5,376,800 |
| drug45 | Cotellic | $7,449,385 | $7,711,295 | $23,610,004 | | | $38,770,684 | $38,770,684 |
| drug97 | Cresemba | | | $14,501,632 | | | $14,501,632 | $14,501,632 |
| drug270 | Crysvita | $7,479,804 | $1,822,386 | $22,692,745 | $5,479,598 | | $31,994,935 | $37,474,533 |
| drug159 | Cyramza | $11,666,722 | $6,071,146 | $60,231,413 | | | $77,969,281 | $77,969,281 |
| drug52 | Daklinza | | $18,434,281 | $3,655,676 | | | $22,089,957 | $22,089,957 |
| drug166 | Daliresp | | | $2,440,872 | | $3,009,428 | $2,440,872 | $5,450,300 |
| drug53 | Dalvance | $1,006,735 | $9,742,847 | | | | $10,749,582 | $10,749,582 |
| drug56 | Darzalex | $7,397,898 | $4,302,919 | $47,557,659 | | | $59,258,476 | $59,258,476 |
| drug271 | Daurismo | | $4,364,964 | $22,772,745 | | | $27,137,709 | $27,137,709 |
| drug57 | Defitelio | | $2,922,451 | $32,400,358 | | | $35,322,809 | $35,322,809 |
| drug82 | Dificid | | $4,565,998 | $2,686,049 | | | $7,252,047 | $7,252,047 |
| drug22 | Duavee | | | $2,762,218 | | | $2,762,218 | $2,762,218 |
| drug226 | Dupixent | | $859,576 | | | | $859,576 | $859,576 |
| drug163 | Edurant | $22,705,886 | $5,002,481 | $5,969,235 | $36,484,203 | | $33,677,602 | $70,161,805 |
| drug189 | Egrifta | | $10,954,470 | $3,613,527 | $85,002,319 | $5,720,385 | $14,567,997 | $105,290,701 |
| drug12 | Eliquis | $636,774 | $3,113,233 | $4,549,148 | $292,987 | $629,999 | $8,299,155 | $9,222,141 |
| drug196 | Ella | $4,375,249 | $7,713,933 | $37,988,318 | | | $50,077,500 | $50,077,500 |

© 2023 Zhou EW et al. *JAMA Health Forum.*

| | | | | | | | | |
|---|---|---|---|---|---|---|---|---|
| drug370 | Eloctate | | $20,945,902 | $13,829,120 | | | $34,775,022 | $34,775,022 |
| drug274 | Elzonris | | | $23,492,885 | | | $23,492,885 | $23,492,885 |
| drug227 | Emflaza | | $2,382,317 | | | | $2,382,317 | $2,382,317 |
| drug71 | Empliciti | $19,891,603 | $9,130,747 | $12,243,623 | | | $41,265,973 | $41,265,973 |
| drug362 | Enhertu | | | $13,940,024 | | | $13,940,024 | $13,940,024 |
| drug170 | Entresto | | | $22,675,812 | $21,922,896 | | $22,675,812 | $44,598,708 |
| drug200 | Entyvio | | | $2,338,301 | | | $2,338,301 | $2,338,301 |
| drug276 | Epidiolex | | $3,220,473 | $6,125,564 | | | $9,346,037 | $9,346,037 |
| drug207 | Erivedge | $21,102,658 | $52,969,292 | $45,929,568 | $12,433,760 | | $120,001,518 | $132,435,278 |
| drug277 | Erleada | $1,771,173 | $8,648,965 | $46,477,774 | | | $56,897,912 | $56,897,912 |
| drug16 | Erwinaze | | $7,984,055 | $5,633,724 | | | $13,617,779 | $13,617,779 |
| drug155 | Esbriet | $1,244,102 | $4,729,217 | | | | $5,973,319 | $5,973,319 |
| drug78 | Exondys 51 | | $11,774,824 | | | | $11,774,824 | $11,774,824 |
| drug5 | Eylea | $13,820,826 | $273,285,088 | $105,695,701 | $213,157 | | $392,801,615 | $393,014,772 |
| drug55 | Farxiga | | | $17,844,255 | $10,702,094 | | $17,844,255 | $28,546,349 |
| drug142 | Farydak | $35,257,709 | $54,619,018 | $86,644,292 | | | $176,521,019 | $176,521,019 |
| drug228 | Fasenra | $6,891,474 | $6,057,273 | | | | $12,948,747 | $12,948,747 |
| drug211 | Ferriprox | $2,907,052 | $16,500,814 | $13,051,304 | $28,139,795 | | $32,459,170 | $60,598,965 |
| drug89 | Firazyr | | | $26,389,605 | $12,908,752 | | $26,389,605 | $39,298,357 |
| drug278 | Firdapse | | $16,415,784 | | | | $16,415,784 | $16,415,784 |
| drug49 | Fulyzaq | | $2,544,882 | | | | $2,544,882 | $2,544,882 |
| drug279 | Galafold | | | $6,433,969 | | | $6,433,969 | $6,433,969 |
| drug186 | Gattex | | | $1,403,330 | | | $1,403,330 | $1,403,330 |
| drug131 | Gazyva | | $19,395,621 | $83,074,713 | | | $102,470,334 | $102,470,334 |
| drug73 | Genvoya | | | $13,756,907 | $3,421,337 | $2,239,860 | $13,756,907 | $19,418,104 |
| drug229 | Giapreza | $23,958,104 | $124,063,353 | $279,783,167 | $145,205,291 | $5,153,584 | $427,804,624 | $578,163,499 |
| drug84 | Gilenya | | $4,048,428 | $3,097,625 | | | $7,146,053 | $7,146,053 |
| drug4 | Gilotrif | $14,699,307 | $29,882,283 | $65,393,030 | $12,433,760 | | $109,974,620 | $122,408,380 |
| drug76 | Halaven | $30,323,919 | $98,735,963 | $64,716,960 | | | $193,776,842 | $193,776,842 |
| drug102 | Harvoni | $3,108,611 | $26,852,647 | $91,359,893 | $44,977,131 | | $121,321,151 | $166,298,282 |
| drug86 | Horizant | | | | $1,208,179 | | $0 | $1,208,179 |

© 2023 Zhou EW et al. *JAMA Health Forum.*

| | | | | | | | | |
|---|---|---|---|---|---|---|---|---|
| drug141 | Ibrance | | $103,871,803 | $134,434,168 | | | $238,305,971 | $238,305,971 |
| drug158 | Iclusig | $14,442,110 | $9,981,384 | $89,659,067 | | | $114,082,561 | $114,082,561 |
| drug231 | Idhifa | $2,537,278 | $453,709 | $38,797,543 | | | $41,788,530 | $41,788,530 |
| drug88 | Imbruvica | $51,522,601 | $136,556,464 | $394,415,741 | | | $582,494,806 | $582,494,806 |
| drug232 | Imfinzi | $21,892,509 | $56,146,238 | $105,759,443 | | | $183,798,190 | $183,798,190 |
| drug375 | Imlygic | | | $19,792,448 | $3,369,849 | | $19,792,448 | $23,162,297 |
| drug120 | Impavido | | | $1,781,373 | $1,132,780 | | $1,781,373 | $2,914,153 |
| drug187 | Incivek | $16,358,056 | $28,267,779 | $37,493,638 | $24,818,712 | | $82,119,473 | $106,938,185 |
| drug20 | Inlyta | $7,435,565 | $40,920,985 | $124,708,581 | | | $173,065,131 | $173,065,131 |
| drug335 | Inrebic | | $12,766,523 | $14,684,495 | | | $27,451,018 | $27,451,018 |
| drug36 | Invokana | $4,128,660 | $961,852 | $567,866 | $483,714 | | $5,658,378 | $6,142,092 |
| drug169 | Jakafi | $24,563,673 | $94,944,674 | $180,470,500 | | | $299,978,847 | $299,978,847 |
| drug74 | Jardiance | $1,966,717 | | $11,580,160 | $7,393,118 | | $13,546,877 | $20,939,995 |
| drug34 | Jevtana | $9,525,071 | $27,443,493 | $30,810,421 | | | $67,778,985 | $67,778,985 |
| drug111 | Juxtapid | | | $23,617,674 | | | $23,617,674 | $23,617,674 |
| drug3 | Kadcyla | $5,263,360 | $12,769,619 | $158,810,272 | | | $176,843,251 | $176,843,251 |
| drug99 | Kalydeco | | $184,728,975 | $226,627,749 | $28,139,795 | $1,541,127 | $411,356,724 | $441,037,646 |
| drug171 | Kanuma | | $6,518,215 | | $16,692,723 | | $6,518,215 | $23,210,938 |
| drug37 | Kengreal | | $4,416,096 | $3,904,723 | | | $8,320,819 | $8,320,819 |
| drug149 | Keytruda | $82,758,684 | $214,667,304 | $267,178,494 | | | $564,604,482 | $564,604,482 |
| drug235 | Kisqali | | | $33,528,326 | | | $33,528,326 | $33,528,326 |
| drug378 | Kovaltry | | $51,219,583 | $30,953,971 | | | $82,173,554 | $82,173,554 |
| drug282 | Krintafel | | | $1,537,408 | | | $1,537,408 | $1,537,408 |
| drug148 | Krystexxa | $207,078 | $964,130 | | | | $1,171,208 | $1,171,208 |
| drug59 | Kybella | $5,836,740 | $4,012,881 | $10,730,433 | | $15,259,480 | $20,580,054 | $35,839,534 |
| drug381 | Kymriah | $13,143,858 | $5,703,787 | | $8,499,181 | | $18,847,645 | $27,346,826 |
| drug121 | Kynamro | $8,977,152 | | | | | $8,977,152 | $8,977,152 |
| drug38 | Kyprolis | $10,672,880 | $76,184,148 | $232,588,394 | $2,423,026 | | $319,445,422 | $321,868,448 |
| drug134 | Lartruvo | | $4,483,853 | $29,971,959 | | | $34,455,812 | $34,455,812 |
| drug103 | Lenvima | $340,930 | $2,526,556 | $114,912,825 | | | $117,780,311 | $117,780,311 |
| drug283 | Libtayo | | | $29,015,418 | | | $29,015,418 | $29,015,418 |

© 2023 Zhou EW et al. *JAMA Health Forum.*

| | | | | | | | | |
|---|---|---|---|---|---|---|---|---|
| drug191 | Lonsurf | $4,913,789 | $7,072,180 | $21,204,381 | | | $33,190,350 | $33,190,350 |
| drug285 | Lorbrena | $419,107 | $650,862 | | | | $1,069,969 | $1,069,969 |
| drug286 | Lucemyra | $3,767,400 | | $8,751,467 | | | $12,518,867 | $12,518,867 |
| drug9 | Lumizyme | | $3,479,907 | | | | $3,479,907 | $3,479,907 |
| drug287 | Lumoxiti | $12,154,165 | $1,097,442 | $17,793,479 | $4,111,614 | | $31,045,086 | $35,156,700 |
| drug383 | Luxturna | | | $21,898,606 | | | $21,898,606 | $21,898,606 |
| drug133 | Lynparza | $10,726,788 | $23,524,314 | $94,912,943 | | $707,065 | $129,164,045 | $129,871,110 |
| drug236 | Mavyret | | $741,688 | $3,531,158 | | | $4,272,846 | $4,272,846 |
| drug195 | Mekinist | $16,654,620 | $184,647,581 | $212,310,915 | | | $413,613,116 | $413,613,116 |
| drug288 | Mektovi | | $2,051,421 | $59,736,382 | | | $61,787,803 | $61,787,803 |
| drug237 | Mepsevii | | | $21,772,607 | | | $21,772,607 | $21,772,607 |
| drug123 | Movantik | | | $1,311,274 | | | $1,311,274 | $1,311,274 |
| drug119 | Myalept | $1,072,887 | $24,670,533 | $636,307 | $35,719,579 | $591,359 | $26,379,727 | $62,690,665 |
| drug122 | Myrbetriq | $19,519,958 | $3,699,340 | | | | $23,219,298 | $23,219,298 |
| drug60 | Natazia | | | $968,751 | | | $968,751 | $968,751 |
| drug143 | Natpara | $47,896,567 | $198,021,727 | $154,344,553 | $93,355,693 | $77,618,306 | $400,262,847 | $571,236,846 |
| drug238 | Nerlynx | | $60,064,260 | $84,784,211 | | | $144,848,471 | $144,848,471 |
| drug11 | Nesina | | | $22,640,093 | | | $22,640,093 | $22,640,093 |
| drug213 | Neutroval | | $17,008,617 | | | | $17,008,617 | $17,008,617 |
| drug100 | Ninlaro | | $64,475,628 | $45,883,748 | | | $110,359,376 | $110,359,376 |
| drug64 | Northera | | | $235,690 | | | $235,690 | $235,690 |
| drug118 | Nucala | | $4,773,351 | $21,468,050 | $1,535,619 | | $26,241,401 | $27,777,020 |
| drug24 | Nulojix | | $1,826,103 | $740,885 | $787,147 | | $2,566,988 | $3,354,135 |
| drug154 | Nuplazid | | $1,936,240 | $198,768 | | | $2,135,008 | $2,135,008 |
| drug129 | Ocaliva | $4,301,763 | $110,697,400 | $85,308,343 | $22,815,311 | | $200,307,506 | $223,122,817 |
| drug239 | Ocrevus | | | $17,582,331 | | | $17,582,331 | $17,582,331 |
| drug177 | Odomzo | $2,109,533 | $7,268,478 | $29,685,514 | | | $39,063,525 | $39,063,525 |
| drug126 | Ofev | | $40,264,984 | $27,995,154 | | | $68,260,138 | $68,260,138 |
| drug175 | Olysio | | $4,548,350 | | | | $4,548,350 | $4,548,350 |
| drug212 | Onfi | | | $6,541,945 | | | $6,541,945 | $6,541,945 |
| drug127 | Opdivo | $89,013,521 | $261,300,798 | $386,135,458 | | | $736,449,777 | $736,449,777 |

© 2023 Zhou EW et al. *JAMA Health Forum.*

| | | | | | | | | |
|---|---|---|---|---|---|---|---|---|
| drug296 | Orilissa | | | $7,935,839 | | | $7,935,839 | $7,935,839 |
| drug115 | Orkambi | | $8,440,512 | $103,852,701 | | | $112,293,213 | $112,293,213 |
| drug140 | Osphena | $486,801 | | | | | $486,801 | $486,801 |
| drug13 | Otezla | | $11,013,227 | $3,480,764 | | | $14,493,991 | $14,493,991 |
| drug240 | Ozempic | | | $14,013,455 | | | $14,013,455 | $14,013,455 |
| drug361 | Padcev | | | $22,292,327 | | | $22,292,327 | $22,292,327 |
| drug298 | Palynziq | | | $6,590,216 | | | $6,590,216 | $6,590,216 |
| drug152 | Perjeta | $8,058,905 | $37,566,306 | $154,057,255 | | | $199,682,466 | $199,682,466 |
| drug325 | Piqray | $11,280,731 | $6,124,664 | $79,621,336 | | | $97,026,731 | $97,026,731 |
| drug356 | Polivy | | | $13,940,024 | | | $13,940,024 | $13,940,024 |
| drug157 | Pomalyst | $15,025,573 | $99,757,098 | $69,760,537 | $292,987 | | $184,543,208 | $184,836,195 |
| drug124 | Portrazza | | | $4,846,046 | | | $4,846,046 | $4,846,046 |
| drug300 | Poteligeo | | | $25,857,493 | | | $25,857,493 | $25,857,493 |
| drug50 | Pradaxa | $0 | | $123,541 | | $629,999 | $123,541 | $753,540 |
| drug10 | Praluent | $7,480,617 | | $2,330,380 | $2,423,026 | | $9,810,997 | $12,234,023 |
| drug332 | Pretomanid | $114,055,658 | | $46,017,172 | $12,433,760 | | $160,072,830 | $172,506,590 |
| drug242 | Prevymis | | | $23,207,166 | | | $23,207,166 | $23,207,166 |
| drug58 | Prolia | | $2,715,077 | $68,338,967 | $89,521,951 | $4,931,965 | $71,054,044 | $165,507,960 |
| drug363 | Provenge | | $18,299,286 | $16,195,236 | | | $34,494,522 | $34,494,522 |
| drug348 | Recarbrio | | | $983,353 | | | $983,353 | $983,353 |
| drug80 | Repatha | | $10,266,545 | $854,552 | | | $11,121,097 | $11,121,097 |
| drug32 | Rexulti | | | $2,183,091 | | | $2,183,091 | $2,183,091 |
| drug366 | Rixubis | $628,150 | | | | | $628,150 | $628,150 |
| drug333 | Rozlytrek | | $2,477,975 | $13,442,482 | | | $15,920,457 | $15,920,457 |
| drug168 | Rubraca | | $2,332,799 | $30,635,163 | | | $32,967,962 | $32,967,962 |
| drug245 | Rydapt | $16,337,445 | $21,488,290 | $84,474,145 | | | $122,299,880 | $122,299,880 |
| drug66 | Savaysa | | | $1,187,322 | | | $1,187,322 | $1,187,322 |
| drug340 | Scenesse | | | $2,419,650 | | | $2,419,650 | $2,419,650 |
| drug144 | Signifor | $8,152,673 | $4,130,833 | $68,946,970 | | | $81,230,476 | $81,230,476 |
| drug246 | Siliq | $155,597 | $2,745,793 | | | | $2,901,390 | $2,901,390 |
| drug23 | Sirturo | $139,479,337 | | | $12,433,760 | | $139,479,337 | $151,913,097 |

© 2023 Zhou EW et al. *JAMA Health Forum.*

| | | | | | | | | |
|---|---|---|---|---|---|---|---|---|
| drug176 | Sovaldi | $7,379,459 | $138,888,896 | $96,281,752 | $68,139,552 | | $242,550,107 | $310,689,659 |
| drug161 | Stivarga | | $26,997,098 | $29,828,224 | | | $56,825,322 | $56,825,322 |
| drug15 | Strensiq | | $777,657 | $21,772,607 | | | $22,550,264 | $22,550,264 |
| drug321 | Sunosi | | | $7,791,381 | | | $7,791,381 | $7,791,381 |
| drug174 | Sylvant | | $84,680,900 | $62,148,735 | | | $146,829,635 | $146,829,635 |
| drug303 | Symdeko | | $27,157,905 | $22,833,828 | | | $49,991,733 | $49,991,733 |
| drug136 | Synribo | | $5,400,618 | $46,801,158 | | | $52,201,776 | $52,201,776 |
| drug51 | Tafinlar | $14,125,957 | $134,691,908 | $158,538,187 | | | $307,356,052 | $307,356,052 |
| drug139 | Tagrisso | $1,258,771 | $2,562,174 | $11,415,037 | | | $15,235,982 | $15,235,982 |
| drug101 | Taltz | $8,536,118 | $6,289,343 | $2,943,714 | | | $17,769,175 | $17,769,175 |
| drug305 | Talzenna | | $41,299,240 | $66,378,588 | | | $107,677,828 | $107,677,828 |
| drug306 | Tavalisse | | $7,157,263 | | | | $7,157,263 | $7,157,263 |
| drug17 | Tecentriq | $11,748,611 | $37,189,604 | $101,883,970 | | | $150,822,185 | $150,822,185 |
| drug41 | Teflaro | $3,444,517 | | | | | $3,444,517 | $3,444,517 |
| drug308 | Tibsovo | $5,719,958 | | $41,154,789 | | | $46,874,747 | $46,874,747 |
| drug63 | Tivicay | $3,214,003 | $151,567,729 | $116,406,516 | $99,983,018 | | $271,188,248 | $371,171,266 |
| drug108 | Tradjenta | | | $11,080,111 | $13,403,980 | | $11,080,111 | $24,484,091 |
| drug250 | Tremfya | | | $1,493,229 | | | $1,493,229 | $1,493,229 |
| drug95 | Tresiba | | | $67,741,082 | $11,323,535 | | $67,741,082 | $79,064,617 |
| drug342 | Trikafta | | $14,113,408 | $15,564,300 | | | $29,677,708 | $29,677,708 |
| drug209 | Trintellix | | | | $9,625,428 | | $0 | $9,625,428 |
| drug65 | Trulicity | | | $25,779,884 | $5,634,223 | | $25,779,884 | $31,414,107 |
| drug330 | Turalio | | $4,626,653 | $58,184,078 | | | $62,810,731 | $62,810,731 |
| drug252 | Tymlos | | $182,670 | | | | $182,670 | $182,670 |
| drug62 | Unituxin | $87,209,945 | $113,303,095 | $75,569,576 | | | $276,082,616 | $276,082,616 |
| drug173 | Uptravi | | | $189,135 | | | $189,135 | $189,135 |
| drug79 | Vascepa | $5,585,344 | | | | $11,114,293 | $5,585,344 | $16,699,637 |
| drug145 | Veltassa | $151,165 | | | | | $151,165 | $151,165 |
| drug204 | Venclexta | $10,224,798 | $28,131,859 | $82,413,526 | | | $120,770,183 | $120,770,183 |
| drug254 | Verzenio | | | $24,388,357 | | | $24,388,357 | $24,388,357 |
| drug109 | Victoza | $1,973,491 | $31,609,912 | $158,823,196 | $11,879,306 | $5,455,629 | $192,406,599 | $209,741,534 |

© 2023 Zhou EW et al. *JAMA Health Forum.*

| | | | | | | | | |
|---|---|---|---|---|---|---|---|---|
| drug29 | Victrelis | $12,658,169 | $5,386,318 | $4,026,467 | $4,356,453 | $598,546 | $22,070,954 | $27,025,953 |
| drug137 | Viekira Pak | | $20,203,486 | $241,381,121 | | | $261,584,607 | $261,584,607 |
| drug70 | Vimizim | | | $43,671,892 | | | $43,671,892 | $43,671,892 |
| drug312 | Vitrakvi | $6,942,022 | $26,233,357 | $51,267,769 | | | $84,443,148 | $84,443,148 |
| drug313 | Vizimpro | | $833,770 | $44,121,922 | | | $44,955,692 | $44,955,692 |
| drug376 | Vonvendi | | $48,045,302 | $30,430,359 | | | $78,475,661 | $78,475,661 |
| drug255 | Vosevi | | $626 | | | | $626 | $626 |
| drug201 | Vpriv | | | $26,325,223 | | | $26,325,223 | $26,325,223 |
| drug324 | Vyndaqel | | $725,457 | $651,954 | | | $1,377,411 | $1,377,411 |
| drug48 | Xalkori | $61,970,128 | $88,858,249 | $62,835,534 | | | $213,663,911 | $213,663,911 |
| drug165 | Xarelto | | | $15,955,076 | | $896,250 | $15,955,076 | $16,851,326 |
| drug193 | Xeljanz | $140,994 | $857,067 | $1,206,544 | | | $2,204,605 | $2,204,605 |
| drug259 | Xermelo | | $5,348,979 | | $3,369,849 | | $5,348,979 | $8,718,828 |
| drug214 | Xofigo | $209,407 | $2,300,000 | $82,503,481 | | | $85,012,888 | $85,012,888 |
| drug316 | Xospata | | $6,584,504 | $2,413,609 | | | $8,998,113 | $8,998,113 |
| drug327 | Xpovio | $986,544 | $26,885,079 | $54,196,972 | | | $82,068,595 | $82,068,595 |
| drug75 | Xtandi | $2,359,238 | $78,270,290 | $153,041,928 | | $7,461,554 | $233,671,456 | $241,133,010 |
| drug198 | Xuriden | $2,915,745 | $46,313,800 | | | | $49,229,545 | $49,229,545 |
| drug96 | Yervoy | $78,074,612 | $278,005,695 | $415,211,835 | $3,369,849 | | $771,292,142 | $774,661,991 |
| drug382 | Yescarta | $6,882,366 | $3,193,648 | $29,516,391 | | | $39,592,405 | $39,592,405 |
| drug194 | Yondelis | $20,067,616 | $90,414,465 | $158,596,473 | $3,369,849 | | $269,078,554 | $272,448,403 |
| drug210 | Zaltrap | $13,820,826 | $273,285,088 | $105,695,701 | $213,157 | | $392,801,615 | $393,014,772 |
| drug260 | Zejula | | $7,011,794 | | | | $7,011,794 | $7,011,794 |
| drug203 | Zelboraf | $8,692,236 | $24,933,505 | $176,719,158 | | | $210,344,899 | $210,344,899 |
| drug215 | Zinbryta | $17,977,247 | $27,894,680 | | | | $45,871,927 | $45,871,927 |
| drug27 | Zinplava | | | $1,161,891 | | | $1,161,891 | $1,161,891 |
| drug208 | Zontivity | | $1,664,106 | | $1,075,253 | | $1,664,106 | $2,739,359 |
| drug104 | Zurampic | | | $2,163,270 | | | $2,163,270 | $2,163,270 |
| drug91 | Zydelig | $2,459,856 | $1,822,215 | $163,822,610 | | | $168,104,681 | $168,104,681 |
| drug43 | Zykadia | $383,971 | $385,097 | $19,681,178 | | | $20,450,246 | $20,450,246 |
| drug1 | Zytiga | $9,887,965 | $172,748,678 | $241,347,972 | | $7,461,554 | $423,984,615 | $431,446,169 |

© 2023 Zhou EW et al. *JAMA Health Forum.*

## eTable 5. Total Grant Type Project Years and NIH Funding Distribution for Basic, Applied, and Development Research

| A. Grant type Unique Project Years by phase | | | | | | | |
|---|---|---|---|---|---|---|---|
| | Basic and Applied Research | Development Research | | | | | |
| **Grant Type** | **Total Project Years** | **Phase 1** | **Phase 2** | **Phase 3** | **Phase 4** | **Total P1-3 Project Years** | **Total Phased Project Years** |
| Cooperative Agreements[a] | 17,824 (5.6%) | 102 | 487 | 295 | 42 | 884 | 960 (33.9%) |
| Intramural Programs | 19,084 (5.9%) | 111 | 242 | 109 | 24 | 462 | 510 (18.0%) |
| Research Projects | 207,897 (64.8%) | 58 | 122 | 48 | 29 | 228 | 281 (9.9%) |
| Fellowship/Training Programs | 47,232 (14.7%) | 100 | 179 | 23 | 5 | 302 | 316 (11.2%) |
| General Clinical Research Centers/ Research Program Projects and Centers | 25,110 (7.8%) | 173 | 340 | 187 | 23 | 700 | 751 (26.5%) |
| Others | 3,876 (1.2%) | 6 | 6 | 2 | 1 | 14 | 16 (0.6%) |
| Total APY | 321,023 | 550 | 1,376 | 664 | 124 | 2,590 | 2,834 |

| B. Grant type total funding by phase | | | | | | | |
|---|---|---|---|---|---|---|---|
| | Basic and Applied Research | Development Research | | | | | |
| **Grant Type** | **Total NIH funding (millions 2018 USD)** | **Phase 1** | **Phase 2** | **Phase 3** | **Phase 4** | **Total P1-3 Funding** | **Total Phased Funding** |
| Cooperative Agreements | $48,115.9 (19.5%) | $696.7 (47.7%) | $1,962.3 (56.0%) | $1,741.2 (68.1%) | $363.1 (85.1%) | $4,400.2 (58.5%) | $4,850.0 (59.9%) |
| Intramural Programs | $18,729.1 (7.6%) | $90.8 (6.2%) | $161.2 (4.6%) | $7.1 (0.3%) | $1.5 (0.4%) | $259.1 (3.5%) | $266.4 (3.3%) |
| Research Projects | $99,730.9 (40.3%) | $53.1 (3.6%) | $119.4 (3.4%) | $64.9 (2.5%) | $12.7 (3.0%) | $237.3 (3.2%) | $261.2 (3.2%) |
| Fellowship/Training Programs | $14,902.2 (6.0%) | $35.1 (2.4%) | $85.4 (2.4%) | $32.5 (1.3%) | $10.0 (2.4%) | $153.0 (2.0%) | $176.3 (2.2%) |
| General Clinical Research Centers/ Research Program Projects and Centers | $63,130.0 (25.5%) | $583.0 (39.9%) | $1,167.4 (33.3%) | $710.9 (27.8%) | $34.0 (7.9%) | $2,461.3 (32.7%) | $2,531.3 (31.2%) |
| Others | $2,716.4 (1.1%) | $0.8 (0.1%) | $6.1 (0.2%) | 0% | $5.4 (1.3%) | $6.9 (0.1%) | $18.0 (0.2%) |
| Total Funding | $247,324.6 | $1,459.5 | $3,501.6 | $2,556.5 | $426.8 | $7,517.7 | $8,103.2 |

a - Manual review of a random set of 300 Cooperative Agreements showed that 96.2% of these projects involved Clinical Translational Science Award, clinical trial networks, centers, or consortia involved in clinical research.

© 2023 Zhou EW et al. *JAMA Health Forum.*

## eTable 6. Average Per-Phase NIH Investment on Drugs With Grant Support Only, Similar to SWOG Analysis

| | Phase 1 | Phase 2 | Phase 3 | Phase 1-3[a] |
|---|---|---|---|---|
| Average per drug spending by phase (not including zeros)[b] | $34,799,889 | $56,682,319 | $33,730,099 | $125,212,308 |
| Standard deviation | $44,883,389 | $81,271,185 | $57,501,775 | $126,983,947 |
| Confidence level (95.0%) | $8,044,871 | $12,531,389 | $9,246,107 | $16,147,157 |
| Mean (95% CI) (millions)[c] | 34.8 (26.8-42.8) | 56.7 (69.2 - 44.2) | 33.7 (24.5-42.9) | 125.2 (109.1-141.4) |

a - Phase 1-3 average was taken as the sum of averages from phase 1,2,3 to accurately reflect analysis without zero funding data points. b - Analysis was done with highest phase rounded to unique project years after dropping data points with no NIH funding to match similar conditions from past works by the SWOG.[32] c - Drug costs reported by the SWOG ($123.6 million/drug).

© 2023 Zhou EW et al. *JAMA Health Forum.*
