# Supplementary material for: Spending on Phased Clinical Development of Approved Drugs by the US National Institutes of Health Compared With Industry
Source: JAMA Health Forum. 2023 Jul 14;4(7):e231921. doi: 10.1001/jamahealthforum.2023.1921 (PMC10349341; doi:10.1001/jamahealthforum.2023.1921)
Supplement: Supplement 2. — Data Sharing Statement [file jamahealthforum-e231921-s002.pdf]

Data Sharing Statement

Zhou. Spending on Phased Clinical Development of Approved Drugs by the US National Institutes of Health Compared With Industry. *JAMA Health Forum*. Published July 14, 2023. doi:10.1001/jamahealthforum.2023.1921

Data
Data available: Yes
Data types: Data (not involving human participants), Other (please specify)
Additional Information: Python code
How to access data: Python code: (https://github.com/BentleySciIndustry/NIH-Contribution-to-phased-clinical-development-of-drugs-approved-Supplemental-Data-Sharing.git). Quantitative data: in supplemental files.
When available: With publication

Supporting Documents
Document types: Statistical/analytic code
How to access documents: (https://github.com/BentleySciIndustry/NIH-Contribution-to-phased-clinical-development-of-drugs-approved-Supplemental-Data-Sharing.git)
When available: beginning date: 12-09-2022

Additional Information
Who can access the data: Unrestricted availability
Types of analyses: Unrestricted availability
Mechanisms of data availability: Online access without restriction
Any additional restrictions: Unrestricted availability
